# Mapping the Evidence on the Effectiveness of Telemedicine Interventions in Diabetes, Dyslipidemia, and Hypertension: An Umbrella Review of Systematic Reviews and Meta-Analyses

**DOI:** 10.2196/16791

**Published:** 2020-03-18

**Authors:** Patrick Timpel, Sarah Oswald, Peter E H Schwarz, Lorenz Harst

**Affiliations:** 1 Department for Prevention and Care of Diabetes Faculty of Medicine Carl Gustav Carus Technische Universität Dresden Dresden Germany; 2 Master Program Health Sciences / Public Health at the Institute and Policlinic of Occupational and Social Medicine Faculty of Medicine at the University Clinic Carl Gustav Carus Technische Universität Dresden Dresden Germany; 3 Paul Langerhans Institute Dresden Helmholtz Center Munich University Hospital and Faculty of Medicine, Technische Universität Dresden Dresden Germany; 4 German Center for Diabetes Research (DZD e V) Neuherberg Germany; 5 Research Association Public Health Saxony / Center for Evidence-Based Healthcare Faculty of Medicine Carl Gustav Carus Technische Universität Dresden Dresden Germany

**Keywords:** telemedicine, diabetes mellitus, hypertension, dyslipidemia, review, GRADE approach, treatment outcome

## Abstract

**Background:**

Telemedicine is defined by three characteristics: (1) using information and communication technologies, (2) covering a geographical distance, and (3) involving professionals who deliver care directly to a patient or a group of patients. It is said to improve chronic care management and self-management in patients with chronic diseases. However, currently available guidelines for the care of patients with diabetes, hypertension, or dyslipidemia do not include evidence-based guidance on which components of telemedicine are most effective for which patient populations.

**Objective:**

The primary aim of this study was to identify, synthesize, and critically appraise evidence on the effectiveness of telemedicine solutions and their components on clinical outcomes in patients with diabetes, hypertension, or dyslipidemia.

**Methods:**

We conducted an umbrella review of high-level evidence, including systematic reviews and meta-analyses of randomized controlled trials. On the basis of predefined eligibility criteria, extensive automated and manual searches of the databases PubMed, EMBASE, and Cochrane Library were conducted. Two authors independently screened the studies, extracted data, and carried out the quality assessments. Extracted data were presented according to intervention components and patient characteristics using defined thresholds of clinical relevance. Overall certainty of outcomes was assessed using the Grading of Recommendations, Assessment, Development, and Evaluation (GRADE) tool.

**Results:**

Overall, 3564 references were identified, of which 46 records were included after applying eligibility criteria. The majority of included studies were published after 2015. Significant and clinically relevant reduction rates for glycated hemoglobin (HbA_1c_; ≤−0.5%) were found in patients with diabetes. Higher reduction rates were found for recently diagnosed patients and those with higher baseline HbA_1c_ (>8%). Telemedicine was not found to have a significant and clinically meaningful impact on blood pressure. Only reviews or meta-analyses reporting lipid outcomes in patients with diabetes were found. GRADE assessment revealed that the overall quality of the evidence was low to very low.

**Conclusions:**

The results of this umbrella review indicate that telemedicine has the potential to improve clinical outcomes in patients with diabetes. Although subgroup-specific effectiveness rates favoring certain intervention and population characteristics were found, the low GRADE ratings indicate that evidence can be considered as limited. Future updates of clinical care and practice guidelines should carefully assess the methodological quality of studies and the overall certainty of subgroup-specific outcomes before recommending telemedicine interventions for certain patient populations.

## Introduction

### Background

Diabetes is affecting 463 million people worldwide (aged between 20 and 79 years) [1]. Hypertension and hyperlipidemia (or hypercholesterolemia) are common comorbidities in patients with type 2 diabetes (T2D), and also show an increasing coprevalence [2,3]. The risk of diabetes-associated complications can be reduced by continuous control of blood glucose [4], blood pressure (BP) lowering [5-8], and blood lipid profile [9,10]. Current guidelines of the American Diabetes Association (ADA) recommend that most adults with diabetes achieve glycated hemoglobin (HbA_1c_) <7.0%, BP<140/90 mmHg (<130/90 for patients with increased cardiovascular [CV] risk), and low-density lipoprotein cholesterol (LDL-c) <100 mg/dL [11]. Diabetes self-management education and support, defined as an interactive and continuous process intended to increase knowledge, skills, and abilities required for successful self-management of diabetes interventions [12], has proven to be effective [13,14]. Similarly, hypertensive patients may benefit from the combination of self-monitoring with education or counseling in terms of increased medication adherence and improved BP control [15].

The application of information and communication technologies (ICTs) in health care has been rapidly increasing worldwide. Telemedicine is defined by three characteristics: (1) using ICTs, (2) covering a geographical distance, and (3) involving professionals who deliver care directly to a patient or a group of patients [16,17]. Owing to the need for individualized and continuous monitoring and self-management support for patients, chronic diseases are considered the ideal target conditions for the development and implementation of telemedicine approaches [18,19].

However, detailed guidance is still lacking on how to choose and integrate tools for specific target groups in diabetes care [20,21]. Earlier systematic reviews of high-quality review articles already uncovered key elements for technology-enabled self-management, such as (1) communication between a health care provider and patient, (2) patient-generated health data, (3) education, and (4) feedback [22], or they simply underlined the promising nature of telemedicine [23]. However, the available overviews mainly focus on a specific target group, do not take into account the heterogeneity of telemedicine applications, or focus on a specific tool [24]. Heterogeneous applications of the term telemedicine [16] further limit the external validity of single studies. Owing to the differentiated phenotypes of applied telemedicine solutions, their components, and settings, as well as missing analyses of the quality of studies (and certainty of effects), evidence-based guidance on the best available digital intervention is challenging [25-27].

### Objective

Therefore, the primary objective of this umbrella review is to identify, synthesize, and critically appraise the evidence on the effectiveness of telemedicine solutions and their components on clinical outcomes—HbA_1c_, high-density lipoprotein (HDL), low-density lipoprotein (LDL), total cholesterol (TC), triglycerides (TGC), systolic BP (SBP), diastolic BP (DBP)—in patients with diabetes (type 1 diabetes [T1D] and T2D), hypertension, or dyslipidemia. Owing to the increasing number of available reviews and meta-analysis as well as the potential of addressing three prevalent chronic conditions with multiple digital interventions, the analysis was conducted as an umbrella review [28,29].

The research question is based on the Population, Intervention, Control, Outcome, and Time (PICOT) criteria: *In patients with diabetes, hypertension or dyslipidemia, what is the evidence for the effectiveness of telemedicine-supported chronic care on disease-specific clinical outcomes?*

## Methods

### Search Strategy and Eligibility Criteria

We conducted an umbrella review using extensive automated and manual searches of the databases PubMed, EMBASE, and the Cochrane Library to identify relevant evidence on the effectiveness of telemedicine interventions on the three target diseases. Umbrella reviews summarize and contrast evidence from existing systematic reviews and meta-analyses by looking at specific outcomes across included records [28].

The search was carried out in October 2018. PICOT-criteria (Table 1) for “population,” “intervention,” and “study design” were combined to develop the search strings (Multimedia Appendix 1)*.* No time limitation was applied.

**Table 1:** Population, Intervention, Control, Outcome, and Time and eligibility criteria.

Population, Intervention, Control, Outcome, and Time criteria	Eligibility	
	Inclusion	Exclusion
Population	Humans; only studies addressing at least one of the predetermined target diseases within their initial search	Studies addressing chronic diseases in general, other than the three diseases defined, or not addressing any disease at all; specific populations (pregnant women and ethnical minorities); and animals
Intervention	Primary studies applying telemedicine intervention specified as (1) using ICTs^a^, (2) covering distance, and (3) involving a health care provider for delivering care to the patient	Studies focusing solely on monitoring or data storage and exchange tools (such as electronic health records)
Control	Usual care	No control group available or not specified
Outcome	Effectiveness analyses allowing for quantitative comparisons between groups using clinical parameters (primary outcome HbA_1c_^b^, SBP^c^, DBP^d^, HDL-c^e^, LDL-c^f^, TC^g^, and TGC^h^)	Studies primarily investigating mortality, costs or cost-effectiveness, or feasibility; or efficacy
Time	Follow-up time of at least three months	No or shorter follow-up periods described
Study design	Study design being either a systematic review or meta-analysis of randomized controlled trials	Other, including a systematic review or meta-analysis of observational studies

^a^ICT: information and communication technology.

^b^HbA_1c_: glycated hemoglobin.

^c^SBP: systolic blood pressure.

^d^DBP: diastolic blood pressure.

^e^HDL-c: high-density lipoprotein cholesterol.

^f^LDL-c: low-density lipoprotein cholesterol.

^g^TC: total cholesterol.

^h^TGC: triglycerides.

Records that fulfilled the following eligibility criteria were included (Table 1): systematic reviews or meta-analyses of randomized controlled trials (RCTs; as this is regarded as highest level of evidence) [30] evaluating the effectiveness of telemedicine in at least one of the target diseases (diabetes, hypertension, and/or dyslipidemia) in adults. No restrictions were made with respect to the kind of participating medical providers. We included all eligible articles in English language and with full text available.

Relevant reviews or meta-analyses were excluded if their primary studies mainly assessed mortality, utilization of health services, the usability of the technology studied, or patients’ acceptance of or satisfaction with the telemedicine tools, or if no quantitative comparison based on clinical outcomes was reported. Studies evaluating interventions using automated feedback without involving a professional or those providing only monitoring of relevant parameters (without feedback) were excluded. In addition, studies evaluating telemedicine use of medical providers only or those in which the components of the intervention were not transparently described were excluded. Eligible records had to report a change in one of the specified clinical outcomes after a follow-up time of at least three months, as this period is in line with current treatment guidelines [15,31,32].

Conference abstracts or protocols were excluded as well. Research was excluded if it focused on specific countries or regions or targeted specified populations (eg, minorities and pregnant women with diabetes). We excluded those studies for which updates of the evidence—indicated by the same group of authors and/or application of identical search string—were available.

We further searched the reference lists of all relevant publications by hand, to identify any additional studies. After carrying out the title-abstract screening, we conducted a hand search in Google Scholar and the three most relevant journals in the field of digital health, as indicated by the highest number of potentially relevant publications (Multimedia Appendix 2).

### Data Extraction and Quality Assessment

Two authors (PT and LH) independently screened the records, extracted data, and carried out the quality assessments. The quality assessment of records was done using the Oxford Quality Assessment Questionnaire (OQAQ) to eliminate records of low quality before data extraction [33]. Any disagreement over the suitability of certain records was discussed among the raters and resolved by consensus.

As the Grading of Recommendations, Assessment, Development, and Evaluation (GRADE) is the established tool for assessing the overall certainty of evidence by analyzing its risk of bias, imprecision, inconsistency, indirectness, and publication bias, it was used to assess the quality of included records [34]. This assessment was performed by three independent researchers (PT, SO, and LH), using independent pairwise ratings. Disagreements were again resolved by discussion or, where not possible, by consulting the independent third coder [35].

The results of the included records were extracted using a piloted, standardized data extraction form. According to the methodological considerations for conducting umbrella or meta-reviews, the results were reported descriptively and in tabular form [28,29].

### Data Analysis

The presentation of data is descriptive; however, the results of meta-analyses and subgroup analyses were specifically analyzed to find effective components or modes of delivery (intensity and frequency) in subgroups or settings. In light of previous trials, a clinically relevant reduction of –0.5% in HbA_1c_ is considered a suitable threshold (Table 2) [36,37]*.* The definition of clinically relevant reduction rates (direction of arrows) and the statistical significance (green) were used to compare interventions’ effectiveness (Tables 3-5).

**Table 2:** Definition of clinically relevant differences in glycated hemoglobin.

Reduction rate in glycated hemoglobin (%)	*P* value	Guidance
≤−0.5	>.05	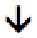 ^a^
>−0.5, <0	>.05	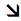 ^b^
>0	>.05	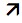 ^c^
>−0.5, <0	<.05	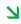 ^d^
≤−0.5	<.05	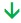 ^e^

^a^non-significant but clinically relevant change.

^b^non-significant and not clinically relevant change.

^c^non-significant and not clinically relevant change.

^d^significant but not clinically relevant change.

^e^significant and clinically relevant change.

In terms of BP control, a −10 mmHg reduction in SBP or a −5 mmHg reduction in DBP is considered as clinically relevant [38]. No exact clinical relevance margins for lipid profiles could be prespecified, as European guidelines recommend a risk-based approach with regard to the presence of CV risk or established CV disease [32].

To compare overall treatment effects between baseline and follow-up, meta-analyses reporting treatment effects as mean differences (MD), standardized mean difference (SMD), Cohen *d*, and Hedge *g* were included. For heterogeneity testing, results of I^2^ statistics (indicating variation across studies that is not due to chance) were used. A value of <40% indicates a low, 30%-60% a moderate, and >75% a substantial-to-high level of heterogeneity [39].

## Results

### Review Characteristics

**Figure 1:**
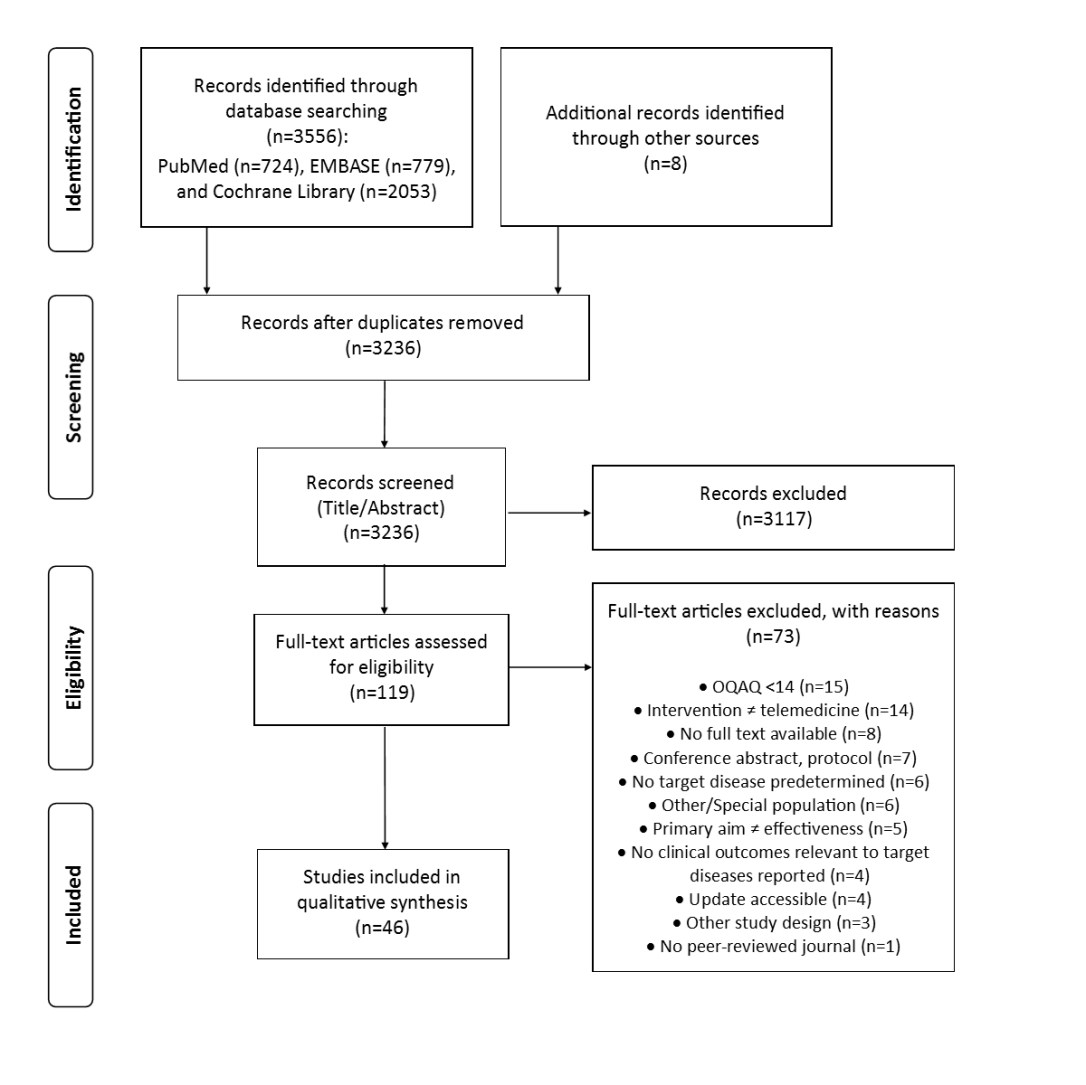
Preferred Reporting Items for Systematic Reviews and Meta-analyses flowchart of the study selection process. OQAQ: Overview Quality Assessment Questionnaire.

Overall, 3564 references were identified. After title-abstract screening, 119 records remained for further full-text analysis. Details of the extracted evidence are provided in the Multimedia Appendices 3-9. The most important reasons for exclusion were low quality (n=15) and applied interventions not matching the prespecified telemedicine definition (n=14; annex section V). Overall, 46 studies were included in this umbrella review (Figure 1). In Figure 1, the Preferred Reporting Items for Systematic Reviews and Meta-analyses flowchart shows the study selection process, covering the single steps of identification via a 2-step screening (title and abstract and full-text base) for eligibility and inclusion into the qualitative synthesis of this review.

### Study Characteristics

Study designs included 16 systematic reviews [40-55], 7 meta-analyses [56-62], 19 records conducting both a systematic review and meta-analysis [63-81], three systematic reviews and meta-analyses with meta-regression [82-84], and one systematic review and network meta-analysis [85]. The included meta-studies were published between 2009 and 2018, the majority was published after 2015 (Multimedia Appendix 5) and focused on diabetes. No high-quality reviews or meta-analyses were found targeting patients with dyslipidemia.

An analysis of primary studies revealed significant overlaps among the 26 meta-analyses (Multimedia Appendix 5). The majority of primary studies were published after 2010 (Multimedia Appendix 5).

On a scale of 0 to 18, the median OQAQ score of the 46 included studies was 16 (IQR 1), indicating that they were good quality systematic reviews and meta-analyses.

### Results of Included Systematic Reviews

Data from 16 systematic reviews were extracted (Multimedia Appendix 6) [40-54]. Diabetes was the chronic disease covered most often by the included reviews. A total of 5 systematic reviews dealt with T2D [41,43,46,49]; however, only one systematic review dealt with T1D [53]. Both types were studied together for a total of 4 times [42,45,48,51], four other systematic reviews did not specify which type of diabetes they focused on [44,50,52,54]. Among the other diseases studied, hypertension was the most common [40,44,50,55]. The results of the included systematic reviews were mixed, presenting a tendency for positive effects of telemedicine, in general, and digitally supported self-management using phones or apps on HbA_1c_ [42,44,54] and SBP/DBP [40,55]. In contrast, the majority of studies evaluating telemonitoring and self-monitoring interventions found no significant improvements in HbA_1c_, fasting plasma glucose, or BP [49-51].

### Results of Meta-Analyses

#### Effectiveness of Telemedicine in Patients With Diabetes

Digital self-management in diabetes (T1D and T2D) was analyzed by 13 meta-analyses, of which 4 meta-analyses evaluated the effectiveness of mobile health (mHealth) [63,65,84] and one meta-analysis evaluated the use of social network services (SNS) [78]. In digital self-management interventions, those including prescription (−0.75%, 95% CI −1.05 to −0.43; *P*=.013), teleconsultation (−0.62%; *P*<.001), and health information technologies on top of usual care (mostly based on tele-education; −0.57%, 95% CI −0.71 to −0.43; *P*<.005) showed significant clinically relevant mean reductions in HbA_1c_ [60,72,80]. Digital self-management interventions using mHealth showed significant clinically relevant reductions in HbA_1c_ if they provided remote access to usual care (−0.55%, 95% CI −0.72 to −0.38; *P*<.001), used one or two features (−0.52%, 95% CI −0.76 to −0.28; *P*<.001), used SMS-based feedback (−0.64%, 95% CI −1.09 to −0.19; *P*=.005), included a potential risk intervention (−0.61%, 95% CI −0.95 to −0.27; *P*<.001), included a structured display (−0.69%, 95% CI −0.32 to −1.06; *P*=.008), provided medication management (−0.56%, 95% CI −0.99 to −0.13; *P*<.001), and provided lifestyle modification management (−0.52%, 95% CI −0.84 to −0.20; *P*<.001) [63,65,80]. SNS applied in diabetes self-management interventions proved to be effective if they were Web-based (−0.51%, 95% CI −0.68 to −0.34; *P*<.001) or combined Web-based SNS with mobile technologies (−0.54%, 95% CI −0.72 to −0.37; *P*<.001) [78].

Overall mean reductions in HbA_1c_ of telemedicine interventions in patients with T1D ranged between −0.12% and −0.86% [60,61,63,70,72,78,82,84]. Overall mean reductions were mostly not significant. Highest mean reductions were observed for the combination of tele-education with teleconsultation (−0.91%, 95% CI −1.21 to −0.61), although data on statistical significance were not provided [70]. No significant clinically relevant reductions for population characteristics such as baseline HbA_1c_ or age were identified in patients with T1D.

Although varying in range (−0.01% to −1.13%), telemedicine significantly reduced HbA_1c_ in patients with T2D [59,60,64,66,68,72,73,75,76,78,79,81,85].

#### Effectiveness According to Intervention Duration

Table 3 summarizes the effectiveness of telemedicine in patients with diabetes by comparing intervention durations.

**Table 3:** Effectiveness of telemedicine on glycated hemoglobin in patients with diabetes, according to intervention duration.

Application category and type of diabetes		Intervention duration	Trials, n	Patients, n	Outcome	MD^a^ (95% CI) of percent change in HbA_1c_^b^	*P* value	I^2^ (%)	Grading of Recommendations, Assessment, Development, and Evaluation
**Digital health education** **[56]**									
	T1D^c^/T2D^d^	3 months	3	203	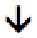 ^e^	−0.71 (−1.0 to −0.43)	.90	0	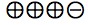
	T1D/T2D	6 months	2	562	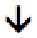 ^e^	−0.52 (−0.75 to −0.29)	.65	0	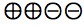
	T1D/T2D	12 months	6	1153	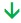 ^e,f^	−0.55 (−0.7 to −0.39)	<.001	78	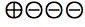
**Telemedicine** **[70,85]**									
	T1D	<6 months	7	NS^g^	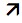 ^e^	0.07 (−0.16 to 0.31)	NS	NS	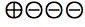
	T1D	≥6 months	21	NS	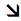 ^e^	−0.24 (−0.41 to −0.07)	NS	NS	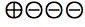
	T2D	≤3 months	17	1377	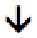 ^e^	−0.67 (−0.93 to −0.41)	NS	NS	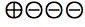
	T2D	4-6 months	36	4538	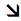 ^e^	−0.41 (−0.84 to 0.02)	NS	NS	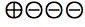
	T2D	7-11 months	4	659	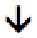 ^e^	−0.66 (−1.18 to −0.15)	NS	NS	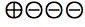
	T2D	≥12 months	36	10,237	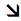 ^e^	−0.26 (−0.40 to −0.12)	NS	NS	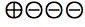
**Digital self-management** **[59,60,72,83]**									
	T2D	≤3 months	10	NS	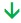 ^e,f^	−0.51 (−0.71 to −0.31)	<.001	41.8	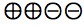
	T2D	>3 and ≤6 months	10	NS	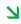 ^e,f^	−0.48 (−0.68 to −0.28)	<.001	34.5	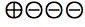
	T2D	3-4 months	11	1613	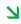 ^e,f^	−0.30 (−0.50 to −0.11)	<.001	89.1	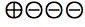
	T2D	>6 months	15	NS	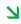 ^e,f^	−0.35 (−0.53 to −0.18)	<.001	70.5	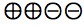
	T2D	6-8 months	14	2389	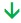 ^e,f^	−0.59 (−0.78 to −0.39)	<.001	84.8	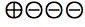
	T2D	9-12 months	7	1272	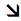 ^e^	−0.21 (−0.35 to −0.075)	.131	39.1	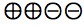
	T1D/T2D	≤ 6 months	30	NS	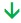 ^e,f^	−0.56 (NS)	<.001	30	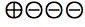
	T1D/T2D	6 months	6	741	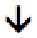 ^e^	−0.57 (−0.85 to −0.30)	.099	NS	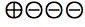
	T1D/T2D	>6 months	25	NS	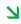 ^e,f^	−0.40 (NS)	<.001	25	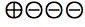
	T1D/T2D	12 months	7	3466	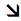 ^e^	−0.30 (−0.48 to −0.11)	.099	NS	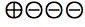
**Digital self-management (SMS)** **[75]**									
	T2D	<6 months	6	NS	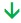 ^e,f^	−0.60 (−0.80 to −0.40)	<.001	NS	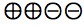
	T2D	≥6 months	4	NS	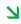 ^e,f^	−0.40 (−0.56 to −0.24)	<.001	NS	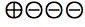
**Digital self-management (social network service)** **[78]**									
	T1D/T2D	≤3 months	13	799	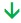 ^e,f^	−0.54 (−0.80 to −0.28)	<.001	23	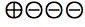
	T1D/T2D	3-12 months	11	1465	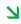 ^e,f^	−0.41 (−0.63 to −0.19)	<.001	25	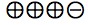
	T1D/T2D	>12 months	10	2713	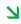 ^e,f^	−0.36 (−0.59 to −0.14)	<.002	90	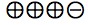

^a^MD: mean difference.

^b^HbA_1c_: glycated haemoglobin

^c^T1D: type 1 diabetes.

^d^T2D: type 2 diabetes.

^e^The direction of the arrows indicates potential clinically relevant reduction rates (see Table 2).

^f^Green arrows show statistical significance.

^g^NS: not specified—cases in which no data were provided. Missing data on statistical significance were handled as nonsignificant.

Significant and clinically relevant reductions were found for short (≤3 months), middle (4-8 months), and long (>12 months) intervention durations. Digital health education, analyzed in the meta-analysis by Angeles et al [56], on average, reduced HbA_1c_ above the predefined clinical relevance margin (HbA_1c_ ≤−0.5%; Table 2). However, only the effects of interventions with a long-term study duration (12 months) were statistically significant (−0.55%, 95% CI −0.7 to −0.39; *P*<.001). Although three meta-analyses observed a tendency for higher reduction rates in shorter intervention durations [59,75,85], no general significant differences in reduction rates among intervention durations were found.

Short-term intervention durations (≤6 months) of digital self-management showed greater mean reductions (−0.56%; *P*<.001) [60] compared with mid- and long-term durations (>6 months) [60,72]. Clinically relevant mean reductions in SNS were significant for short-term intervention durations (≤3 months) as well [78]. Using Web-based SNS for digital self-management proved to be significantly effective in the three pooled follow-up measurements. Again, the greatest mean reductions were apparent during the short-term (≤3 months) follow-up (−0.54%, 95% CI −0.80 to −0.28; *P*<.001) [78].

#### Effectiveness According to Feedback Mode, Frequency, and Intensity

Although telemedicine interventions using feedback functions significantly reduced HbA_1c_ in several studies [56,60-63,66,67,72,80], the highest reduction rates were found when no personalized feedback was provided (−0.61%, 95% CI −1.40 to 0.19; *P*=.001) [63]. No difference in HbA_1c_ change was found for the type of health care professionals providing the feedback (eg, nurses or physicians) [72].

In addition, feedback, provided either via human telephone calls (−1.13%, 95% CI −1.51 to −0.75; *P*<.05) or via the internet (−0.62%, 95% CI −0.82 to −0.42; *P*<.001), significantly reduced HbA_1c_ to a clinically relevant extent (≤−0.5 change) [68,81]. Higher frequency of provider feedback also showed greater reductions in HbA_1c_ (−1.12%, 95% CI −1.32 to −0-91; *P*<.001) when compared with mean reduction rates of interventions utilizing low frequency rates (−0.33%, 95% CI −0.59 to −0.07; *P*<.01) [82] (Table 4).

**Table 4:** Effectiveness of telemedicine on glycated hemoglobin in patients with diabetes, according to feedback mode, frequency, and intensity.

Application category and type of diabetes		Feedback characteristics	Trials, n	Patients, n	Outcome	MD^a^ (95% CI) of percent change in HbA_1c_	*P* value	I^2^ (%)	Grading of Recommendations, Assessment, Development, and Evaluation
**Telemedicine** **[70,82]**									
	T1D^b^	App based	5	336	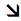 ^c^	−0.37 (−0.94 to 0.20)	.20	81.74	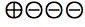
	T1D	High intensity^d^	13	NS	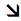 ^c^	−0.24 (−0.49 to 0.01)	NS^e^	NS	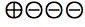
	T1D	≠ High intensity	14	NS	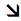 ^c^	−0.09 (−0.23 to 0.06)	NS	NS	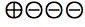
	T1D	Audit + feedback	24	NS	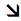 ^c^	−0.22 (−0.38 to −0.06)	NS	NS	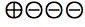
	T1D	No audit + feedback	4	NS	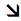 ^c^	0.01 (−0.27 to −0.30)	NS	NS	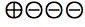
**Digital self-management** **[59,68,72,81,83]**									
	T2D^f^	Human call/telephone	5	NS	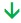 ^c,g^	−1.13 (−1.51 to −0.75)	<.05	38	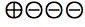
	T2D	Human call/telephone	12	NS	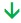 ^c,g^	−0.53 (−0.81 to −0.26)	<.001	76.35	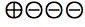
	T2D	Manual	6	1180	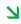 ^c,g^	−0.44 (−0.74 to −0.15)	.04	NS	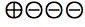
	T2D	Manual	22	NS	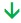 ^c,g^	−0.50 (−0.65 to −0.34)	<.001	67.2	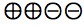
	T2D	Automated	5	NS	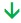 ^c,g^	−0.50 (−0.69 to −0.32)	<.001	0	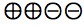
	T2D	Automated calls	2	NS	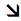 ^c^	−0.01 (−0.32 to 0.29)	.94	0	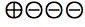
	T2D	Automated text	9	NS	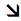 ^c^	−0.36 (−0.47 to −0.24)	NS	0	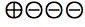
	T2D	Text message	3	380	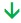 ^c,g^	−0.52 (−1.04 to 0.00)	<.05	73.5	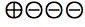
	T2D	Web-based	13	2405	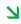 ^c,g^	−0.41 (−0.55 to −0.27)	<.05	79.6	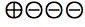
	T2D	Web-based	19	NS	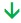 ^c,g^	−0.62 (−0.82 to −0.42)	<.001	77.57	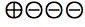
**Digital self-management (mobile health)** **[63,82,84]**									
	T2D	Low frequency	7	440	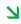 ^c,g^	−0.33 (−0.59 to −0.07)	.01	47.35	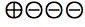
	T2D	High frequency	5	326	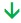 ^c,g^	−1.12 (−1.32 to −0.91)	<.001	0	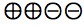
	T1D/T2D	Personalized feedback	8	NS	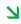 ^c,g^	−0.43 (−0.74 to −0.12)	<.001	75	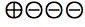
	T1D/T2D	≠ Personalized feedback	4	NS	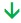 ^c,g^	−0.61 (−1.40 to 0.19)	.001	81	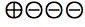
	T1D/T2D	Frequency (daily)	15	NS	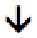 ^c^	−0.6 (−0.9 to −0.4)	.27	NS	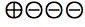
	T1D/T2D	Frequency (weekly)	3	NS	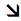 ^c^	−0.2 (−0.6 to 0.2)	.27	NS	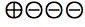
	T1D/T2D	Frequency (not specified)	4	NS	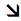 ^c^	−0.4 (−0.5 to −0.2)	.27	NS	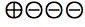

^a^MD: mean difference.

^b^T1D: type 1 diabetes.

^c^The direction of the arrows indicates potential clinically relevant reduction rates (see Table 2).

^d^Direct contact at least once a week.

^e^NS: not specified—cases in which no data were provided. Missing data on statistical significance were handled as nonsignificant.

^f^T2D: type 2 diabetes.

^g^Green arrows show statistical significance.

The meta-regression carried out by Huang et al [68] also revealed that factors we previously disregarded, such as study location, sample size, and feedback methods, were associated significantly with changes in HbA_1c_. Their combination in multivariate meta-regression analyses explained almost 100% of the variance among studies.

#### Effectiveness According to Population Characteristics

Subgroup analyses on the effectiveness of telemedicine in certain patient populations (Table 5) were carried out by 12 meta-analyses [60-62,66,68,70,72,75,79,83-85].

Although differences were not always significant, those subgroups with higher baseline HbA_1c_ (>7.5% or >8.0%) showed increased reductions rates [62,68,70,72,79,83,85]. Only for interventions categorized as digital self-management using SMS, the reduction rates were higher (−0.71%, 95% CI −0.93 to −0.48; *P*<.001) in patients with lower baseline HbA_1c_ (<8%) when compared with those with higher (≥8%) baseline HbA_1c_ (−0.38%, 95% CI −0.53 to −0.24; *P*<.001) [75]. Using meta-regression methods, Kebede et al [83] found significant reduction rates in HbA_1c_ for baseline HbA_1c_ >7.5% (beta=−.44, 95% CI −0.81 to −0.06; *P*=.031), self-monitoring of behavioral outcomes, such as diets and physical activity (beta=−1.21, 95% CI −1.95 to −0.46; *P*=.009), and for support in problem solving (beta=−1.30, 95% CI −2.05 to −0.54; *P*=.007).

Significant differences for age groups were sparse, as only three meta-analysis found significant reduction rates in patients with T2D [75] and both types combined [60,61]. The meta-analysis by Saffari et al [75] on SMS-based digital self-management found significantly greater mean reductions (*P*=.006) in HbA_1c_ for patients younger than 55 years (−0.65%, 95% CI −0.88 to −0.41; *P*<.001) when compared with the older age group (−0.42%, 95% CI −0.56 to −0.27; *P*<.001) [75]. The greatest significant mean reductions were observed for patients with diabetes aged 41 to 50 years (−1.83%, 95% CI −3.17 to −0.48; *P*<.001) and those over 50 years (−1.05%, 95% CI −1.50 to −0.60; *P*<.001) [60,61].

For digital self-management, a shorter time since diagnosis (<8.5 years) was associated with significantly greater mean reduction in HbA_1c_ (−0.83%, 95% CI −1.10 to −0.56; *P*=.007) when compared with patients being diagnosed more than 8.5 years ago (−0.22%, 95% CI −0.44 to 0.01; *P*=.007) [79]. Similarly, patients diagnosed less than 7 years ago showed higher mean reductions (−0.61%, 95% CI −0.79 to −0.42) compared with their counterparts (−0.37%, 95% CI −0.61 to −0.13; *P*=.03) after using SMS-based digital self-management [75].

**Table 5:** Effectiveness of telemedicine on glycated hemoglobin in patients with diabetes, according to population characteristics.

Category of application and type of diabetes		Population characteristics	Trials, n	Patients, n	Outcome	MD^a^ (95% CI) of percent change in HbA_1c_^b^	*P* value	I^2^ (%)	Grading of Recommendations, Assessment, Development, and Evaluation
**Telemedicine [70,85]**									
	T1D^c^	Adults	15	1256	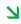 ^d,e^	−0.26 (−0.47 to −0.05)	<.01	79.7	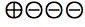
	T1D	Children and adolescents	11	796	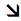	−0.12 (−0.30 to 0.05)	.70	0	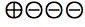
	T1D	Baseline HbA_1c_ <9.0%	16	NS	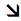	−0.06 (−0.02 to 0.09)	NS^f^	NS	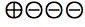
	T1D	Baseline HbA_1c_ ≥9.0%	12	NS	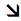	−0.34 (−0.57 to −0.11)	NS	NS	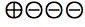
	T2D^g^	Baseline HbA_1c_ <8.0%	48	5720	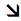	−0.22 (−0.25 to −0.19)	NS	NS	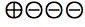
	T2D	Baseline HbA_1c_ ≥8.0%	45	8100	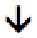	−0.60 (−0.61 to −0.60)	NS	NS	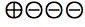
**Digital self-management [60-62,68,72,79,83]**									
	T2D	Age <55 years	7	701	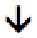	−0.67 (−1.15 to −0.20)	.52	75	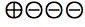
	T2D	Age ≥55 years	8	541	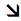	−0.41 (−0.62 to −0.21)	.52	0	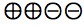
	T2D	Age undetermined	2	289	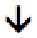	−0.72 (−1.60 to 0.16)	.52	47	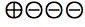
	T2D	Diagnosis^h^ <8.5 years ago	7	549	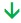	−0.83 (−1.10 to 0.56)	.007	0	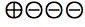
	T2D	Diagnosis^h^ ≥8.5 years ago	4	394	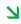	−0.22 (−0.44 to 0.01)	.007	0	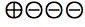
	T2D	Diagnosis time^h^ undetermined	6	588	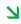	−0.43 (−0.71 to −0.30)	.007	55	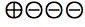
	T2D	Baseline HbA_1c_ ≤8.0%	6	590	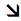	−0.49 (−0.71 to −0.27)	.69	0	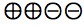
	T2D	Baseline HbA_1c_ ≤8.0%	7	NS	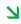	−0.33 (−0.53 to −0.13)	<.05	46	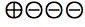
	T2D	Baseline HbA_1c_ >7.0%	11	1707	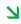	−0.33 (−0.48 to −0.18)	<.001	77.8	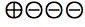
	T2D	Baseline HbA_1c_ >7.5%	10	1921	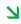	−0.45 (−0.70 to −0.21)	<.001	80.4	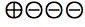
	T2D	Baseline HbA_1c_ >8.0%	11	941	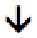	−0.57 (−0.93 to −0.22)	.69	65	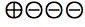
	T2D	Baseline HbA_1c_ >8.0%	11	NS	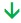	−0.70 (−1.03 to −0.36)	<.05	81	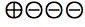
	T2D	Baseline BMI <30 kg/m^2^	5	359	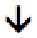	−0.64 (−0.91 to −0.36)	.49	0	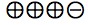
	T2D	Baseline BMI ≥30 kg/m^2^	10	966	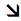	−0.43 (−0.68 to −0.17)	.49	35	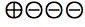
	T2D	Baseline BMI undetermined	2	206	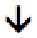	−0.96 (−2.76 to 0.85)	.49	91	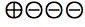
	T1D/T2D	Age <40 years	14	NS	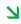	−0.32	.02	NS	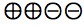
	T1D/T2D	Age <40 years	11	NS	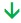	−0.85 (−1.79 to 0.10)	.07	98	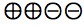
	T1D/T2D	Age ≥40 years	40	NS	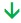	−0.53	<.001	NS	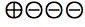
	T1D/T2D	Age 41-50 years	8	NS	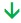	−1.83 (−3.17 to −0.48)	<.001	96.2	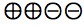
	T1D/T2D	Age >50 years	17	NS	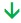	−1.05 (−1.50 to −0.60)	<.001	97	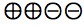
	T1D/T2D	Baseline HbA_1c_ <8.0%	6	NS	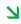	−0.26 (−0.43 to −0.10)	.03	NS	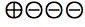
	T1D/T2D	Baseline HbA_1c_ ≥ 8.0%	8	NS	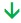	−0.64 (−0.93 to −0.35)	.03	NS	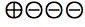
	T1D/T2D	Baseline HbA_1c_ <9.0%	NS	NS	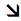	−0.35	NS	NS	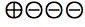
	T1D/T2D	Baseline HbA_1c_ ≥9.0%	NS	NS	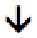	−1.22	NS	NS	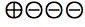
**Digital self-management (mobile health)** **[66,84]**									
	T2D	Baseline HbA_1c_ <8.0%	4	696	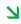	−0.33 (−0.59 to −0.06)	.02	70	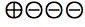
	T1D/T2D	Average age <25 years	5	NS	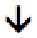	−0.5 (−0.8 to −0.1)	.54	NS	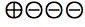
	T1D/T2D	Average age ≥25 years	17	NS	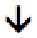	−0.5 (−0.7 to −0.3)	.54	NS	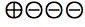
	T1D/T2D	BMI ≥25 kg/m^2^	7	NS	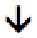	−0.8 (−1.1 to −0.5)	.93	NS	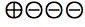
	T1D/T2D	24 kg/m^2^≤ BMI <25 kg/m^2^	3	NS	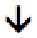	−0.8 (−1.7 to 0.1)	.93	NS	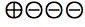
	T1D/T2D	BMI unspecified	12	NS	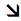	−0.3 (−0.5 to −0.1)	.93	NS	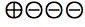
**Digital self-management (SMS)** **[75]**									
	T2D	Age <55 years	5	NS	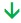	−0.65 (−0.88 to −0.41)	<.001	NS	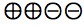
	T2D	Age ≥55 years	5	NS	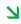	−0.42 (−0.56 to −0.27)	.006	NS	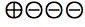
	T2D	Diagnosis^h^ <7 years ago	4	NS	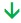	−0.61 (−0.79 to −0.42)	.001	NS	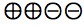
	T2D	Diagnosis^h^ ≥7 years ago	3	NS	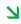	−0.37 (−0.62 to −0.13)	.031	NS	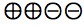
	T2D	Baseline HbA_1c_ <8.0%	5	NS	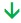	−0.71 (−0.93 to −0.48)	<.001	NS	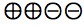
	T2D	Baseline HbA_1c_ ≥8.0%	5	NS	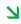	−0.38 (−0.53 to −0.24)	<.001	NS	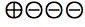

^a^MD: mean difference.

^b^HbA_1c_: glycated hemoglobin.

^c^T1D: type 1 diabetes.

^d^The direction of the arrows indicates potential clinically relevant reduction rates (see Table 2).

^e^Green arrows show statistical significance.

^f^NS: not specified—cases in which no data were provided. Missing data on statistical significance were handled as nonsignificant.

^g^T2D: type 2 diabetes.

^h^Diagnosis time: time since first diagnosis of diabetes.

#### Effect of Telemedicine on Blood Pressure in Patients With Diabetes

Mean reductions of both SBP and DBP were also found in T2D patients. Toma et al [78] found highly significant mean reductions in patients with both T1D and T2D for SBP (−3.47 mmHg, 95% CI −5.01 to −1.94; *P*<.001) and DBP (−1.84 mmHg, 95% CI −2.98 to −0.70; *P*=.002) because of Web- and mobile-based SNS interventions. Evaluating the effect of digitally supported dietary interventions in patients with chronic diseases, Kelly et al [69] also reported significant mean reductions in SBP (−5.91 mmHg, 95% CI −11.14 to −0.68; *P*=.003) in the diabetes subgroup (although showing high heterogeneity between the two studies; I²=69%). Although no information on statistical significance was provided, Lee et al [85] showed greatest mean reductions in SBP for the telemedicine subgroups focusing on tele-education (−4.05 mmHg, 95% CI −5.64 to −1.10), as well as those combining tele-education and telemonitoring (−3.91 mmHg, 95% CI −10.07 to 2.25). Analyzing the data of four studies, Cui et al [66] found nonsignificant reductions for both DBP (−1.76 mmHg, 95% CI −3.6 to 0.07; *P*=.06) and SBP (−2.62 mmHg, 95% CI −5.6 to 0.36; *P*=.08). Digitally supported dietary interventions in patients with diabetes resulted in significant mean reductions in SBP (−5.91 mmHg, 95% CI −11.14 to −0.68; *P*=.003) [69]. However, none of the presented reduction rates reached the predefined clinical relevance margin.

#### Effect of Telemedicine on Lipid Profiles in Patients With Diabetes

Only 8 of the included studies reported on lipid profiles; 4 in T1D/T2D patients [56,65,72,78], 2 in T2D patients [73,85], 1 in T1D patients [70], and 1 in several chronic diseases [69]. On the basis of these studies, evidence on the effectiveness of telemedicine in lowering LDL-c or TGC or increasing high-density lipoprotein cholesterol (HDL-c) in patients with diabetes is scarce and heterogeneous. Marcolino et al [72] found evidence that digital self-management applications for both diabetes types can reduce LDL-c levels; however, although significant, the effect was small (−6.6 mg/dL, 95% CI −8.3 to −4.9; *P*<.001; I²=24%) [72]. In addition, for both types of diabetes, Toma et al [78] found evidence for a significant improvement in TC (−5.74 mg/dL, 95% CI −9.71 to −1.78; *P*<.005; I²=53%), HDL (1.90 mg/dL, 95% CI 0.24 to 3.57; *P*=.02; I²=19%), and TGC (−11.05 mg/dL, 95% CI −20.92 to −1.18; *P*<.03; I²=0%). Reductions in LDL (−1.15 mg/dL, 95% CI −5.19 to 2.88; *P*=.58; I^2^=47%) were not significant. Again, for patients with T2D, the pooled analysis of Lee et al [85] found little and rather inconsistent effects, be it for LDL-c, HDL-c, TC, and TGC.

#### Effects of Telemedicine in Patients With Hypertension

A total of 3 of the included meta-analyses focused on patients with hypertension [57,71]. Although the two analyses of Omboni et al [57,58] focused on home BP monitoring, Liu et al [71] examined the effect of internet-based interventions. Liu et al [71] reported a significant overall mean reduction in SBP (−3.8 mmHg, 95% CI −5.63 to −2.06; *P*=.001) and DBP (−2.1 mmHg, 95% CI −3.51 to −0.65; *P*<.05). Owing to the identified heterogeneity for SBP (I²=61%), the authors carried out a subgroup analysis, revealing that mean change in SBP was greater in long-term interventions (6-12 months; −5.8 mmHg, 95% CI −4.3 to −4.1) when compared with short-term interventions (<6 months; −3.47 mmHg, 95% CI −5.2 to −1.7). However, data on statistical significance were not provided [71]. The results of Omboni et al [57] show significant mean reductions in SBP when using ambulatory measurement (−2.28 mmHg, 95% CI −4.32 to −0.24; *P*<.05). In their more recent analysis, they included studies evaluating additional features such as combined data transmission to physician, feedback, advice, and medication regulation. This time, they observed significant mean reductions (−3.48 mmHg, 95% CI −5.31 to −1.64; *P*<.001) [58].

### Grading of Evidence

The quality assessment of outcomes using the GRADE framework revealed the following levels of certainty (Multimedia Appendix 8)*.* Of the 219 HbA_1c_ outcomes assessed overall, 170 (77.63%) outcomes were rated as very low evidence and 42 (19.18%) outcomes were rated as low evidence. All of the 42 outcomes measuring SBP or DBP resulted in very low ratings of overall certainty (Table 6).

**Table 6:** Grading of Recommendations, Assessment, Development, and Evaluation assessment of certainty of glycated hemoglobin and systolic blood pressure/diastolic blood pressure outcomes.

GRADE^a^	HbA_1c_^b^, n (%)	SBP^c^/DBP^d^, n (%)
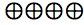	—^e^	—
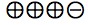	2 (0.92)	—
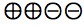	42 (19.8)	—
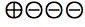	170 (77.63)	42 (100)

^a^GRADE: Grading of Recommendations, Assessment, Development, and Evaluation.

^b^HbA_1c_: glycated hemoglobin.

^c^SBP: systolic blood pressure.

^d^DBP: diastolic blood pressure.

^e^Not applicable.

The main reasons for low-quality assessment results in both outcome categories were as follows:

- Unclear or high-risk of bias: Missing allocation concealment, missing blinding of patients, study personnel and outcome assessors, high risk of selection bias and reporting bias (intention-to-treat analysis), and high or unclear losses to follow-up.
- Inconsistency: High heterogeneity in subgroup analysis, inconsistent confidence intervals crossing the mark for no effect.
- Indirectness: Differences in populations (type of diabetes, baseline HbA_1c_, age, duration of diabetes, and gender), differences in interventions (devices used, components combined, feedback intensity and frequency, and professional or professionals involved), and differences in settings (community, hospital, and primary care) in the pooled subgroups.
- Imprecision: Large confidence intervals and small effect sizes mostly because of small sample sizes.
- Publication bias: Visual and statistical or missing publication bias assessment; the reasons for the increased risk of publication bias mostly referred to the overrepresentation of smaller studies with higher effect sizes (favoring telemedicine). Furthermore, one reason is the paucity of data on mid- and long-term effects (6-12 months).
- Underreporting of relevant information: Reporting of study duration, dropouts/missing data, and follow-up time. Guidance on this matter was further complicated as some authors did not make a clear distinction between study duration and follow-up [61].

Only for two outcomes (0.92%) measuring HbA_1c_, overall certainty was judged as moderate (Tables 5 and 6). In 6 (5 in HbA_1c_ and 1 in DBP) cases, the outcomes of subgroup analyses were not assessed using GRADE, as results of only one trial were used by the authors of meta-analyses to pool data.

As the initial search did not identify records primarily targeting patients with dyslipidemia and subgroup analyses on HDL, LDL, TC, and TGC were sparse, no grading of lipid outcomes was performed.

## Discussion

### Principal Findings

High-level evidence from the 46 included meta-analyses and systematic reviews suggests that telemedicine interventions can be effective in improving clinical outcomes in patients with diabetes. Observed reduction rates are comparable with those of nonpharmacological eg, nutrition intervention [86] or increased physical activity [87]) and some pharmacological interventions (−0.5% to −1.25%) [88]. The observed reduction rates are encouraging, bearing in mind that the United Kingdom Prospective Diabetes Study (UKPDS) revealed that a 0.9% decrease in HbA_1c_ was associated with a 25% reduction in microvascular complications, a 10% decrease in diabetes-related mortality, and a 6% reduction in all-cause mortality [89].

In patients with diabetes, significant differences between telemedicine interventions and for certain population characteristics were identified. Telemedicine interventions embedded in frequent and intense patient-provider interactions and interventions with short durations (≤6 months) showed greater benefits. In addition, higher reduction rates were found for recently diagnosed patients and those with higher baseline HbA_1c_. However, quality assessment using GRADE revealed that overall and subgroup-specific certainty of evidence is low to very low. Therefore, the identified reduction rates have to be dealt with caution when translating them into evidence-based recommendations for treatment guidelines.

Telemedicine was not found to have a significant and clinically meaningful impact on BP. Assessing the certainty of SBP and DBP outcomes, GRADE only revealed very low ratings. No records primarily targeting patients with dyslipidemia were found.

According to the recent consensus report of the ADA and European Association for the Study of Diabetes, the application of telemedicine in diabetes is associated with a modest improvement in glycemic control [31]. The European Society of Cardiology/European Society of Hypertension (ESC/ESH) guidelines for the management of arterial hypertension also report that telemonitoring and mobile phone apps may lead to improved outcomes for patients with hypertension [15]. Our umbrella review updates this assessment of the effectiveness of telemedicine with special regard to intervention components, population characteristics, and it provides an in-depth assessment of the certainty of evidence. A brief summary of the study results can be found in Textbox 1.

**Brief summary of the study results. HbA_1c_: glycated hemoglobin; GRADE: Grading of Recommendations, Assessment, Development, and Evaluation.**

- Telemedicine has the potential to improve clinical outcomes in patients with diabetes. Mixed results were found for patients with hypertension, none for those with dyslipidemia.
- Specific characteristics of the intervention (eg, high frequency and intensity of feedback/interaction and short treatment duration) and the patient (age <55 years, high baseline HbA_1c_, and recent diagnosis) seem to be associated with increased benefits in patients with diabetes.
- An assessment of the overall certainty using GRADE resulted in low and very low ratings, indicating that effects have to be dealt with caution.

#### Intervention Components

Looking at the characteristics of the telemedicine applications analyzed by the included meta-analyses, those encompassing frequent and intense patient-provider communication interactions showed greater benefit in HbA_1c_ reduction. This was especially true for the combination of tele-case management with either teleconsultation (−1.20%, 95% CI −2.30 to −0.10; *P*<.001) or telemonitoring (−0.54%, 95% CI −2.44 to −0.06) in patients with T2D [85]. Similarly, analogue disease self-management education interventions are known to be more effective in terms of HbA_1c_ reduction when they offer additional support (eg, structured dietary or exercise interventions) [37,90]. On the basis of the analysis by Kastner et al [91], the combination of case management and self-management in addition to education provides potential for reduced HbA_1c_ levels when compared with education and plain care coordination. Therefore, continuous and frequent communication, either via intensive feedback [68,81] or psychological support [92], seems most promising.

With a longer duration of follow-ups, the quality of evidence steadily declines because of considerable risk of bias and heterogeneity of study populations and interventions included. As for digital self-management, the evidence base is larger yet more diverse, as SMS (1 meta-analysis), social networks (1 meta-analysis), and mHealth apps (4 meta-analysis) can be used. However, the quality of evidence for digital self-management is low to very low, irrespective of the basal technology or the type of diabetes.

In our analysis, some application types were found to reduce BP, for example, in SBP after tele-education (−4.05 mmHg, 95% CI −5.64 to −1.10), as well as strategies combining tele-education and telemonitoring (−3.91 mmHg) [85]. In patients with diabetes, Web- and mobile-based SNS interventions significantly reduced DBP (−3.47 mmHg) [78], and digitally supported dietary interventions led to significant mean reductions in SBP (−5.91 mmHg) [69]. Although these reduction rates did not reach clinical relevance of ≥10 mmHg in SBP or ≥5 mmHg in DBP, they are similar to the expected reduction rates of nonpharmacologic interventions in patients with hypertension. Our results support the identified potential of telemonitoring and mobile phone apps in home BP self-monitoring, articulated in the current ESC/ESH guideline [15] because of the additional advantages in memorizing, reviewing, and transmitting BP measurements [58,93].

On the basis of the identified potential of telemedicine to provide individual self-management support, it is likely that embedded or additional components may have an additive and/or sustained impact on clinical outcomes. As such, recent evidence identified social media [94,95], gamification [96], and machine learning models [97,98] as successful strategies to improve clinical outcomes and prevent disease-related complications.

#### Population Characteristics

According to the included meta-analyses, telemedicine interventions are more effective for patients with T2D, higher baseline HbA_1c_, and a more recent diagnosis of diabetes. The increased potential for newly diagnosed patients was also identified by systematic reviews [99,100] and landmark trials such as the UKPDS [5]. As for hypertension, the results did not allow for population-specific analyses, which might be because of the rather passive interventions studies, such as telemonitoring.

With the exception of a baseline BMI <30 kg/m^2^ (considered in one meta-analysis), all population-specific subgroup analyses were of low or very low evidence, the latter being more prevalent. This is also true for differences among age groups, for which no significant evidence was found. However, there was a tendency for higher reduction rates of HbA_1c_ in younger patient cohorts with diabetes [60,75]. Owing to the increased risk of elevated BP levels (> 130/80) and long-term risk of CV events, the current ESC/ESH guideline suggests treatment in younger adults (<50 years) [15]. In terms of age-specific BP control, ADA suggests focusing on DBP in patients under 50 years [101].

Overall, as the results concerning population characteristics are diverse and of low to very low quality, our analysis did not find enough high-level evidence to recommend telemedicine for the treatment of patients with both hypertension and diabetes.

Only reviews or meta-analyses reporting lipid outcomes in patients with diabetes were found. The extracted results on lipid outcomes are sparse and too heterogeneous to draw a conclusion on the effectiveness of telemedicine on these outcomes [41,44,46,49,53,55]. With special regard to the effects of statins, as the first-line agents used to decrease cholesterol in the management of dyslipidemia and hypertension, the extracted effects of telemedicine on lipid profiles can be considered as minor [15,32]. However, recent evidence underlines the promising potential of mobile phone-based self-monitoring interventions in patients with lipid metabolism disorders [102], because of the combination of therapy and lifestyle behavior changes.

### Limitations

Robust systematic reviewing methods were used to generate an overview of high-quality evidence on the effects of telemedicine in three prevalent chronic conditions. The protocol of this umbrella review was presented to the research community [103]. However, this study has several limitations, starting with the initial search and inclusion process. Although a comprehensive and piloted search strategy has been used, it is possible that some relevant studies were missed, if the exact search terms were used neither by the authors nor by the databases examined (Multimedia Appendix 10). The search within three different databases, complemented by a comprehensive hand search within the most important journals in the field of telemedicine, the use of MeSH terms, and a broad set of keywords, may have limited this risk of selection bias.

In addition, some full-text articles were excluded because of their definition and application of the term “telemedicine,” which did not comply with standardized definitions, such as the one provided by Sood et al [17]. Although the technology applied to deliver telemedicine has made tremendous advances during the past 10 years, our thorough application of the telemedicine definition and subgroup analyses using the GRADE assessment ensures comparability of intervention types. Intensive full-text assessment was applied to limit the bias of falsely including/excluding systematic reviews and meta-analyses because of mislabeling and inadequate delimitations of efficacy and effectiveness, as studies focusing on efficacy were excluded. As telemedicine is mostly embedded in low-risk interventions, mortality as an outcome was not considered. Although internationally recommended to be reported in addition to changes in HbA_1c_ [104], parameters such as the time below, in, or above range, the number of hypoglycemic episodes, and quality of life were only reported by a few study authors and therefore did not allow for evidence-based guidance on this matter. A reason may be the publication date (median=2011) of the primary studies (Multimedia Appendix 6), which is before these recommendations were made.

We also included different types of statistical analyses, including meta-analysis, network-meta-analysis, and meta-regression. Although the majority reported MD, there was a considerable methodological heterogeneity. This was because of the application of fixed- and random-effects models, as well as the reporting of SMD, Hedge *g*, or Cohen *d* instead of MD. Comparing the aggregated results of those statistical values without considering their weight (on the basis of the number of studies or number of patients per subgroup analysis) may have biased our analysis. However, this process was impeded by inconsistent reporting of baseline data such as the number of trials and participants in subgroup analysis. In addition, it is likely that reporting bias within the included systematic reviews and meta-analysis also affected our analysis. When studying the funnel plots, we also observed a tendency toward overrepresented smaller studies with higher effect sizes (favoring telemedicine), thereby increasing the risk of publication bias within some of the included analyses.

### Further Methodological Considerations

Owing to the multimodal and individualized nature of digital interventions, the low GRADE results, especially the increase I^2^, are not surprising. In addition, we found significant overlaps among the primary studies of the included records (Multimedia Appendix 6). The results of the subgroup analysis therefore need to be considered with exceptional care before recommending certain intervention components for certain populations. However, as GRADE is the established procedure to evaluate the certainty of evidence when developing or updating guidelines, new quality assessment tools appropriate for the tailored and hybrid design of digital interventions should be developed [105]. Along with the need for rather adaptive study designs, there is growing criticism on the suitability of RCTs for evaluating the effectiveness of digital interventions. In light of current efforts to support the clinical effectiveness, quality, and economic value of new technologies by using new assessment frameworks [106-110], our analysis underlines the challenges in this endeavor. In addition, future assessments on the clinical effectiveness should also include consolidated core outcome sets and patient-reported outcomes [111,112]. However, as stated by the included records, longer study durations and more rigorously designed studies are needed for these future research needs.

### Conclusions

The results of this umbrella review indicate that telemedicine has the potential to improve clinical outcomes in patients with diabetes. Evidence extracted from systematic reviews and meta-analyses of RCTs showed subgroup-specific effectiveness rates favoring certain intervention and population characteristics. However, as indicated by the low GRADE ratings, evidence on the effectiveness of telemedicine in the three chronic conditions can be considered as limited.

Future updates of clinical care and practice guidelines should carefully assess the methodological quality of studies and assess the overall certainty of subgroup-specific outcomes before recommending telemedicine interventions for certain patient populations.

## Appendix

Multimedia Appendix 1 Population, Intervention, Control, Outcome, and Time criteria and principles of data extraction.

Multimedia Appendix 2 Number of manuscripts per journal after title/abstract screening.

Multimedia Appendix 3 Quality assessment for study inclusion.

Multimedia Appendix 4 List of excluded studies with reasons.

Multimedia Appendix 5 Characteristics of included records.

Multimedia Appendix 6 Results of included systematic reviews.

Multimedia Appendix 7 Results of included meta-analyses.

Multimedia Appendix 8 Grading of Recommendations Assessment, Development and Evaluation of glycated haemoglobin and diastolic blood pressure/systolic blood pressure outcomes.

Multimedia Appendix 9 References of multimedia appendices.

Multimedia Appendix 10 Electronic database search strategy.

## Abbreviations

ADA: American Diabetes Association
BP: blood pressure
CV: cardiovascular
DBP: diastolic blood pressure
ESC/ESH: European Society of Cardiology/European Society of Hypertension
GRADE: Grading of Recommendations, Assessment, Development, and Evaluation
HbA_1c_: glycated hemoglobin
HDL: high-density lipoprotein
HDL-c: high-density lipoprotein cholesterol
ICT: information and communication technology
LDL: low-density lipoprotein
LDL-c: low-density lipoprotein cholesterol
MD: mean difference
mHealth: mobile health
OQAQ: Oxford Quality Assessment Questionnaire
PICOT: Population, Intervention, Control, Outcome, and Time
RCT: randomized controlled trial
SBP: systolic blood pressure
SMD: standardized mean difference
SNS: social network services
T1D: type 1 diabetes
T2D: type 2 diabetes
TC: total cholesterol
TGC: triglycerides
UKPDS: United Kingdom Prospective Diabetes Study
