# Supplementary material for: Mapping the Evidence on the Effectiveness of Telemedicine Interventions in Diabetes, Dyslipidemia, and Hypertension: An Umbrella Review of Systematic Reviews and Meta-Analyses
Source: J Med Internet Res. 2020 Mar 18;22(3):e16791. doi: 10.2196/16791 (PMC7113804; doi:10.2196/16791)
Supplement: Multimedia Appendix 1 [file jmir_v22i3e16791_app1.doc]

# Multimedia Appendix 2 - Population, Intervention, Control, Outcome, and Time criteria and principles of data extraction

**Suppl. Table 1 Population, Intervention, Control, Outcome, and Time criteria and principles of data extraction**

| **PICOT-Criteria** | | **Data extraction** |
| --- | --- | --- |
| **Population** | Patients with either one or a combination of diabetes, hypertension and / or dyslipidemia | Diagnosis / target disease, n |
| **Intervention** | Telemedicine intervention specified as (1) use of ICT, (2) covering distance and (3) involvement of health care provider delivering care to the patient | Application studied, treatment duration, Setting, component |
| Control | Usual care | Comparator |
| Outcome | Primary outcome HbA1c, SBP, DBP, HDL-c, LDL-c, TC, TGC | Changes in relevant outcome and significance (per component / sub-group); Statistical analysis, Heterogeneity analysis |
| Time | Follow-up time of at least 3 months | Follow Up/ duration |
| **Study Design** | Systematic reviews and / or meta analyses of RCTs | Study design, n of original studies, n of relevant studies with appropriate design |

PICOT criteria were used to inform the overall search, inclusion process and data extraction. PICOT-criteria for population, intervention and study design (all highlighted in blue) were used to develop an overall search string. The same criteria, complemented by information on control, outcome and time, were used for study inclusion. Data extraction was performed using the relevant categories listed in the right column.

DBP = Diastolic blood pressure; EHR = Electronic health record; HbA1c = Glycated haemoglobin; HDL = High-density lipoprotein; LDL = Low-density lipoprotein; RCT = Randomised controlled trial; SBP = Systolic blood pressure; TC = Total cholesterol; TGC = Triglycerides
