# Supplementary material for: Mapping the Evidence on the Effectiveness of Telemedicine Interventions in Diabetes, Dyslipidemia, and Hypertension: An Umbrella Review of Systematic Reviews and Meta-Analyses
Source: J Med Internet Res. 2020 Mar 18;22(3):e16791. doi: 10.2196/16791 (PMC7113804; doi:10.2196/16791)
Supplement: Multimedia Appendix 2 [file jmir_v22i3e16791_app2.doc]

# Multimedia Appendix 3 - Number of manuscripts per journal after title/abstract screening

Suppl. Table 1 Number of manuscripts per journal after title/abstract screening

| **#** | **Journal** |
| --- | --- |
| 13 | Journal of Medical Internet Research |
| 10 | Journal of Telemedicine and Telecare |
| 6 | Journal of Diabetes Science and Technology |
| 3 | Diabetes Research and Clinical Practice |
| 3 | Diabetes Technology and Therapeutics |
| 3 | Diabetic Medicine |
| 3 | International Journal of Medical Informatics |
| 3 | Plos One |
| 3 | TELEMEDICINE and e-HEALTH |
| 3 | The Cochrane Database of Systematic Reviews |
| 2 | BMC Medical Informatics and Decision Making |
| 2 | Canadian Journal of Diabetes |
| 2 | Canadian Medical Association journal |
| 2 | Current Diabetes Reports |
| 2 | Diabetes Care |
| 2 | Diabetes, Obesity and Metabolism |
| 2 | International Journal of Clinical Practice |
| 2 | Journal of Hypertension |
| 2 | JMIR mHealth and uHealth |
| 2 | PLOS Medicine |
| 1 | others (n=39) |

The table lists the journals of the 119 manuscripts considered for inclusion. The 3 most common journals (1) *Journal of Medical Internet Research*, (2) *Journal of Telemedicine and Telecare* and (3) *Journal of Diabetes Science and Technology* were used for hand search.
