# Supplementary material for: Mapping the Evidence on the Effectiveness of Telemedicine Interventions in Diabetes, Dyslipidemia, and Hypertension: An Umbrella Review of Systematic Reviews and Meta-Analyses
Source: J Med Internet Res. 2020 Mar 18;22(3):e16791. doi: 10.2196/16791 (PMC7113804; doi:10.2196/16791)
Supplement: Multimedia Appendix 3 [file jmir_v22i3e16791_app3.doc]

# Multimedia Appendix 4 - Quality assessment for study inclusion

## QUALITY ASSESSMENT TOOL FOR SYSTEMATIC REVIEWS AND META-ANALYSES

A modified version of Oxman and Guyatt OQAQ assessment tool and scale was used to assess the quality of reviews. This consists of the following nine quality interrogations each answerable as ‘yes’, ‘no’ or ‘partially/can’t tell’, carrying scores of 2, 0 and 1, respectively.

1. **Were the search methods used to find evidence on the primary question(s) stated?**
2. **Yes**, description of **databases** searched, **search strategy**, and **years** reviewed. **2 points**.
3. **Partially**, descriptions of methods not complete. **1 point**.
4. **No**, no description of search methods. **0 points**.
5. **Was the search for evidence reasonably comprehensive?**
6. **Yes**, at least one computerised database searched and also a search of unpublished or non-indexed literature. **2 points.**
7. **Can’t tell**, search strategy partially comprehensive, at least one of the strategies performed. **1 point**.
8. **No**, search not comprehensive or not described well. **0 points**.
9. **Were the criteria used for deciding which studies to include in the review reported?**
10. **Yes**, inclusion and exclusion criteria clearly defined. **2 points**.
11. **Partially**, reference to inclusion and exclusion criteria can be found but are not defined clearly enough. **1 point**.
12. **No**, no criteria defined. **0 points**
13. **Was bias in the selection of articles avoided?**
14. **Yes**, issues influencing selection bias were covered. Both of the following bias-avoiding strategies were used: (1) two or more assessors independently judged study relevance, (2) assessors selected studies using predetermined criteria. **2 points**.
15. **Can’t tell**, only one of the strategies used. **1 point**.
16. **No**, selection bias was not avoided or was not discussed. **0 points**.
17. **Were the criteria used for assessing the validity for the studies (i.e. meeting inclusion criteria) reviewed reported?**
18. **Yes**, criteria defined and used addressed the major factors influencing bias. **2 points**.
19. **Partially**, some discussion or reference to criteria. **1 point**.
20. **No**, validity or methodological quality criteria not used or not described. **0 points**.
21. **Were study quality assessment criteria used to inform the review analysis?**
22. **Yes**, criteria were used to inform the analysis, either by exclusion from the analysis if low quality or through sensitivity analysis. **2 points**.
23. **Partially**, some discussion but not clearly described application of criteria. **1 point**.
24. **No**, criteria not used or not described. **0 points**.
25. **Were the methods used to combine the findings of the relevant studies (to reach a conclusion) reported?**
26. **Yes**, qualitative and quantitative methods are acceptable. **2 points.**
27. **Partially**, partial description of methods to combine and tabulate; not sufficient to duplicate. **1 point**.
28. **No**, methods not stated or described. **0 points**.
29. **Were findings of the relevant studies combined appropriately relative to the primary question of the overview?**
30. **Yes**, combining of studies appears acceptable. **2 points**.
31. **Can’t tell**, should be marked if in doubt. **1 point**.
32. **No**, no attempt was made to combine findings, and no statement was made regarding the inappropriateness of combining findings. **0 points**.
33. **Were the conclusions made by the author(s) supported by the data and/or analysis reported in the overview?**
34. **Yes**, data were reported that support the main conclusions regarding the primary question(s) that the overview addresses. **2 points**.
35. **Partially**. **1 point**.
36. **No**, conclusions not supported or unclear. **0 points**.

A maximum score of 18 is possible. Studies with a consented Quality assessment score < 14 are considered as low quality and were excluded from the analysis.

## Quality Assessment of studies included after title/abstract screening

Suppl. Table 1 OQAQ Score per dimension

| **No.** | **Author, year** | **I** | **II** | **III** | **IV** | **V** | **VI** | **VII** | **VIII** | **IX** | **Score** |
| --- | --- | --- | --- | --- | --- | --- | --- | --- | --- | --- | --- |
| 1 | [1] | 2 | 2 | 2 | 1 | 2 | 2 | 1 | 2 | 2 | 16 |
| 2 | [2] | 2 | 2 | 2 | 2 | 2 | 2 | 2 | 1 | 1 | 16 |
| 3 | [3] | 2 | 2 | 2 | 2 | 2 | 2 | 2 | 2 | 2 | 18 |
| 4 | [4] | 2 | 2 | 2 | 2 | 2 | 0 | 1 | 1 | 1 | 16 |
| 5 | [5] | 2 | 2 | 2 | 0 | 2 | 2 | 2 | 2 | 2 | 16 |
| 6 | [6] | 1 | 2 | 2 | 2 | 2 | 1 | 2 | 2 | 2 | 16 |
| 7 | [7] | 2 | 2 | 2 | 0 | 2 | 2 | 0 | 2 | 2 | 13 |
| 8 | [8] | 2 | 1 | 2 | 2 | 2 | 1 | 2 | 2 | 2 | 16 |
| 9 | [9] | 2 | 1 | 2 | 2 | 2 | 0 | 2 | 1 | 1 | 13 |
| 10 | [10] | 2 | 2 | 2 | 2 | 2 | 2 | 2 | 2 | 2 | 18 |
| 11 | [11] | 2 | 1 | 1 | 0 | 2 | 0 | 2 | 2 | 2 | 12 |
| 12 | [12] | 2 | 2 | 2 | 2 | 2 | 2 | 2 | 2 | 2 | 18 |
| 13 | [13] | 2 | 2 | 2 | 2 | 2 | 0 | 2 | 2 | 2 | 16 |
| 14 | [14] | 2 | 2 | 2 | 1 | 2 | 2 | 2 | 2 | 2 | 17 |
| 15 | [15] | 2 | 2 | 2 | 2 | 2 | 2 | 2 | 1 | 2 | 17 |
| 16 | [16] | 2 | 2 | 2 | 0 | 2 | 2 | 1 | 2 | 2 | 15 |
| 17 | [17] | 2 | 1 | 1 | 0 | 1 | 2 | 1 | 2 | 2 | 11 |
| 18 | [18] | 2 | 1 | 2 | 2 | 0 | 0 | 1 | 2 | 1 | 11 |
| 19 | [19] | 2 | 2 | 2 | 0 | 1 | 0 | 2 | 2 | 2 | 13 |
| 20 | [20] | 2 | 1 | 2 | 2 | 1 | 0 | 2 | 1 | 2 | 13 |
| 21 | [21] | 2 | 2 | 2 | 2 | 2 | 2 | 2 | 2 | 2 | 18 |
| 22 | [22] | 1 | 2 | 0 | 2 | 1 | 0 | 2 | 2 | 2 | 12 |
| 23 | [23] | 2 | 1 | 2 | 2 | 2 | 2 | 2 | 2 | 2 | 17 |
| 24 | [24] | 1 | 2 | 2 | 2 | 2 | 2 | 2 | 2 | 2 | 17 |
| 25 | [25] | 2 | 1 | 2 | 2 | 2 | 2 | 1 | 2 | 2 | 16 |
| 26 | [26] | 2 | 2 | 2 | 2 | 2 | 2 | 2 | 2 | 2 | 18 |
| 27 | [27] | 2 | 1 | 2 | 0 | 1 | 0 | 2 | 1 | 2 | 12 |
| 28 | [28] | 2 | 2 | 2 | 1 | 2 | 2 | 2 | 2 | 1 | 16 |
| 29 | [29] | 2 | 2 | 2 | 2 | 2 | 2 | 2 | 2 | 2 | 18 |
| 30 | [30] | 2 | 2 | 2 | 2 | 2 | 2 | 2 | 2 | 1 | 17 |
| 31 | [31] | 2 | 2 | 2 | 0 | 2 | 2 | 2 | 2 | 2 | 16 |
| 32 | [32] | 2 | 2 | 2 | 2 | 2 | 2 | 2 | 2 | 2 | 18 |
| 33 | [33] | 2 | 2 | 2 | 1 | 2 | 2 | 1 | 2 | 2 | 16 |
| 34 | [34] | 2 | 2 | 2 | 2 | 2 | 0 | 2 | 2 | 2 | 16 |
| 35 | [35] | 2 | 2 | 2 | 2 | 2 | 2 | 2 | 2 | 1 | 17 |
| 36 | [36] | 2 | 2 | 2 | 2 | 0 | 2 | 1 | 1 | 1 | 13 |
| 37 | [37] | 2 | 2 | 2 | 2 | 2 | 2 | 2 | 1 | 2 | 17 |
| 38 | [38] | 2 | 1 | 1 | 2 | 2 | 2 | 1 | 2 | 1 | 14 |
| 39 | [39] | 2 | 2 | 2 | 2 | 2 | 2 | 1 | 2 | 1 | 16 |
| 40 | [40] | 2 | 2 | 2 | 2 | 2 | 2 | 2 | 2 | 1 | 17 |
| 41 | [41] | 2 | 1 | 1 | 2 | 2 | 0 | 2 | 2 | 1 | 13 |
| 42 | [42] | 2 | 2 | 2 | 2 | 1 | 2 | 2 | 2 | 1 | 16 |
| 43 | [43] | 2 | 2 | 2 | 2 | 2 | 2 | 2 | 1 | 1 | 16 |
| 44 | [44] | 2 | 2 | 2 | 2 | 2 | 2 | 2 | 2 | 2 | 18 |
| 45 | [45] | 2 | 2 | 2 | 2 | 2 | 2 | 1 | 2 | 2 | 17 |
| 46 | [46] | 2 | 2 | 2 | 2 | 2 | 2 | 2 | 2 | 1 | 17 |
| 47 | [47] | 2 | 1 | 2 | 2 | 1 | 2 | 2 | 0 | 1 | 13 |
| 48 | [48] | 2 | 1 | 2 | 2 | 1 | 2 | 2 | 2 | 2 | 16 |
| 49 | [49] | 2 | 2 | 2 | 2 | 1 | 2 | 2 | 1 | 1 | 15 |
| 50 | [50] | 2 | 1 | 2 | 2 | 2 | 1 | 2 | 2 | 1 | 15 |
| 51 | [51] | 2 | 2 | 2 | 2 | 1 | 2 | 2 | 2 | 1 | 16 |
| 52 | [52] | 2 | 1 | 1 | 1 | 0 | 0 | 0 | 1 | 1 | 7 |
| 53 | [53] | 2 | 2 | 2 | 2 | 2 | 2 | 2 | 1 | 2 | 17 |
| 54 | [54] | 2 | 2 | 2 | 2 | 2 | 2 | 2 | 1 | 2 | 17 |
| 55 | [55] | 2 | 2 | 2 | 2 | 1 | 1 | 2 | 0 | 1 | 13 |
| 56 | [56] | 2 | 0 | 2 | 1 | 2 | 2 | 1 | 1 | 2 | 13 |
| 57 | [57] | 2 | 2 | 2 | 2 | 2 | 2 | 2 | 1 | 2 | 17 |
| 58 | [58] | 2 | 2 | 2 | 2 | 2 | 2 | 2 | 2 | 2 | 18 |
| 59 | [59] | 2 | 2 | 2 | 2 | 2 | 2 | 1 | 2 | 1 | 16 |
| 60 | [60] | 2 | 2 | 2 | 2 | 1 | 2 | 1 | 2 | 2 | 16 |
| 61 | [61] | 2 | 2 | 2 | 2 | 2 | 2 | 2 | 2 | 1 | 17 |

Before conducting the qualitative synthesis of included studies, qualitative assessment of relevant records was performed. Overall results and ratings of the individual nine quality interrogations following the OQAQ questionnaire are provided. Studies with an OQAQ score < 14 were excluded (red); References can be followed up in Multimedia Appendix 10
