# Supplementary material for: Mapping the Evidence on the Effectiveness of Telemedicine Interventions in Diabetes, Dyslipidemia, and Hypertension: An Umbrella Review of Systematic Reviews and Meta-Analyses
Source: J Med Internet Res. 2020 Mar 18;22(3):e16791. doi: 10.2196/16791 (PMC7113804; doi:10.2196/16791)
Supplement: Multimedia Appendix 4 [file jmir_v22i3e16791_app4.doc]

# Multimedia Appendix 5 - List of excluded studies with reasons

Other / Special target population [*n=6*]

[62]

[63]

[64]

[65]

[66]

[67]

No target disease predetermined [*n=6*]

[68]

[69]

[70]

[71]

[72]

[73]

Intervention studied ≠ telemedicine as predetermined [*n=14*]

[74]

[75]

[76]

[77]

[78]

[79]

[80]

[81]

[82]

[83]

[84]

[85]

[86]

[87]

Primary aim of systematic review / meta-analysis ≠ effectiveness [*n=5*]

[88]

[89]

[90]

[91]

[92]

No clinical outcomes relevant to target diseases reported [*n=4*]

[93]

[94]

[95]

[96]

Study design other than systematic review of RCTs or meta-analysis [*n=3*]

[97]

[98]

[99]

Conference abstract or protocol [*n=7*]

[100]

[101]

[102]

[103]

[104]

[105]

[106]

No peer-reviewed journal [*n=1*]

[107]

No fulltext available [*n=8*]

[108]

[109]

[110]

[111]

[112]

[113]

[114]

[115]

Update accessible [*n=4*]

[116]

[117]

[118]

[119]

Quality assessment < 14 (OQAQ) [*n=15*]:

[7]

[9]

[11]

[17]

[18]

[19]

[20]

[22]

[27]

[36]

[41]

[47]

[52]

[55]

[56]
