# Supplementary material for: Mapping the Evidence on the Effectiveness of Telemedicine Interventions in Diabetes, Dyslipidemia, and Hypertension: An Umbrella Review of Systematic Reviews and Meta-Analyses
Source: J Med Internet Res. 2020 Mar 18;22(3):e16791. doi: 10.2196/16791 (PMC7113804; doi:10.2196/16791)
Supplement: Multimedia Appendix 5 [file jmir_v22i3e16791_app5.doc]

# Multimedia Appendix 6- Characteristics of included records

Suppl. Table 1 Profile of the sample

|  | | **Research design** | | |
| --- | --- | --- | --- | --- |
|  |  | Systematic Review (SR) n = 16 | Meta Analysis (MA) n = 7 | SR + MA *  n = 23 |
| Year of publication | ≤2010 | 2 | - | 1 |
|  | >2010≤2015 | 6 | 3 | 9 |
|  | >2015 | 8 | 4 | 13 |
| Target disease | T1D | 1 | - | 1 |
|  | T2D | 4 | 1 | 9 |
|  | T1D / T2D (both) | 6 | 4 | 10 |
|  | Hypertension | 2 | 2 | 2 |
|  | Lipid | - | - | - |
|  | combination | 3 | - | 1 |
| Outcomes reported (at least one) * | HbA1c | 13 | 5 | 20 |
|  | DBP/SBP | 9 | 3 | 7 |
|  | HDL/LDL/TC/TGC | 6 | 1 | 5 |
| OQAQ | ≤15 | 3 | - | 1 |
|  | 16 | 8 | 4 | 7 |
|  | 17-18 | 5 | 3 | 15 |

*T1D = type 1 diabetes; T2D = type 2 diabetes; HbA1c = Glycated haemoglobin; DBP = Diastolic Blood Pressure; SBP = Systolic Blood Pressure; HDL = High-density lipoprotein; LDL = Low-density lipoprotein; TC = Total cholesterol ; TGC = Triglycerides; *multiple outcomes possible. Profile of included systematic reviews and meta-analyses according to year of publication, studied target disease, outcome(s) reported and quality assessment (OQAQ).*

*
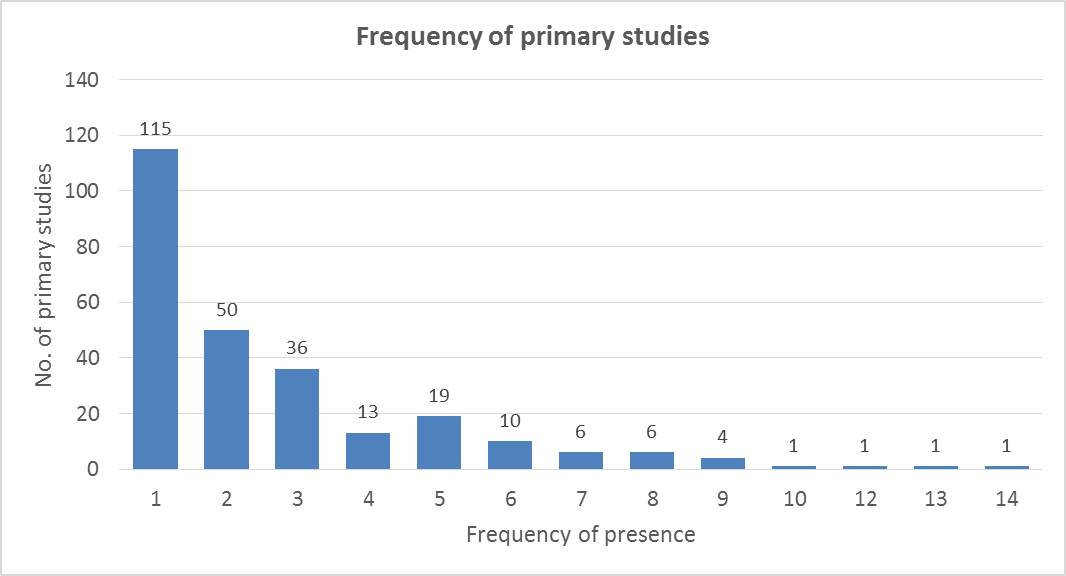
*

Suppl. Figure 1 Frequency of primary studies included in the 26 meta-analyses

*The 26 meta-analyses included 263 primary studies assessing the effectiveness of telemedicine in patients with diabetes, hypertension and / or lipid metabolism disorders. The figure shows the frequency of all primary studies within these 26 meta-analyses.*

*
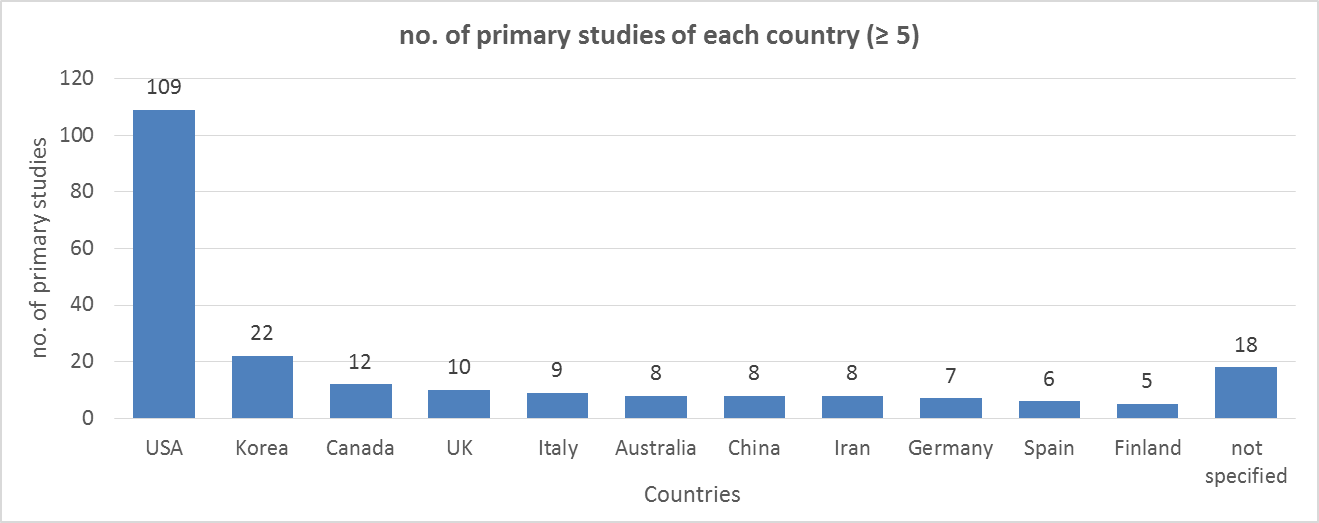
*

Suppl. Figure 2 Number of primary studies included in the 26 meta-analyses per country (≥ 5)

*The 26 meta-analyses included 263 primary studies assessing the effectiveness of telemedicine in patients with diabetes, hypertension and / or lipid metabolism disorders. Primary studies conducted their examinations in 17 different countries (plus the studies where the country was not specified). The figure shows the number of primary studies within these 26 meta-analyses conducted in at least 5 countries.*

*
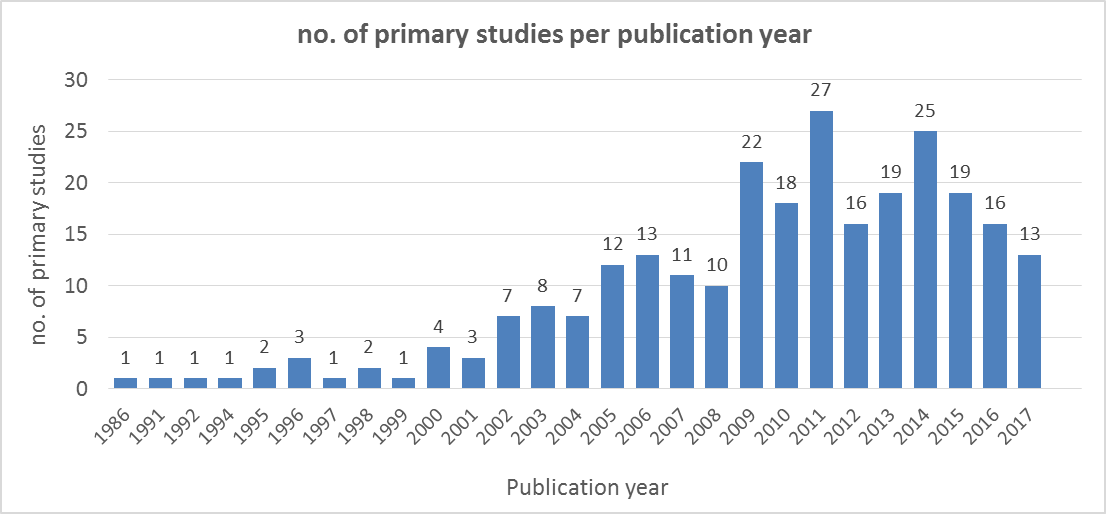
*

Suppl. Figure 3 Number of primary studies included in the 26 meta-analyses per year of publication

*The 26 meta-analyses included 263 primary studies assessing the effectiveness of telemedicine in patients with diabetes, hypertension and / or lipid metabolism disorders. The figure shows the number of primary studies per publication year published between 1986 and 2017 (see also Suppl. Fig.4).*

*
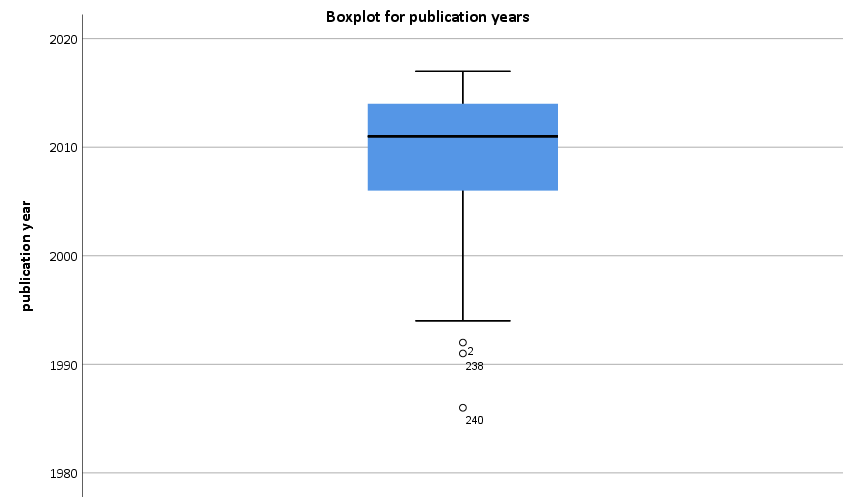
*

Suppl. Figure 4 Boxplot for publication years of primary studies included in the 26 meta-analyses

*The 26 meta-analyses included 263 primary studies assessing the effectiveness of telemedicine in patients with diabetes, hypertension and / or lipid metabolism disorders. The figure shows distribution of publication years of the 263 primary studies between 1986 and 2017 (see also Suppl. Fig.3).*
