# Supplementary material for: Mapping the Evidence on the Effectiveness of Telemedicine Interventions in Diabetes, Dyslipidemia, and Hypertension: An Umbrella Review of Systematic Reviews and Meta-Analyses
Source: J Med Internet Res. 2020 Mar 18;22(3):e16791. doi: 10.2196/16791 (PMC7113804; doi:10.2196/16791)
Supplement: Multimedia Appendix 6 [file jmir_v22i3e16791_app6.doc]

# Multimedia Appendix 7- Results of included systematic reviews

Suppl. Table 1 Description and results of included systematic reviews

| ***Study / year*** | ***Aim*** | ***Target disease*** | ***Conclusion*** |
| --- | --- | --- | --- |
| Alessa et al., 2018 [1] | Effectiveness of apps in lowering blood pressure | Hypertension | significant positive effect on BP: 6/9 studies  non-significant effect on BP: 3/9 studies |
| Aspry et al., 2013 [4] | Impact of Health Information Technologies on clinical lipid outcomes for Diabetes and CVD | T2DM  CVD | significant positive effect on LDL-c: 5/14 studies  non-significant effect on LDL-c: 3/14 studies  significant positive effect on TC: 2/14 studies  no significant effect on HDL-c: 1/14 studies |
| Baron et al., 2012 [5] | Effectiveness of mobile-based applications on HbA1c | T1DM / T2DM | significant positive effect on HbA1c: 11/12 studies (overall)  significant positive effect on HbA1c: 2/2 studies (T1DM)  significant positive effect on HbA1c: 8/9 studies (T2DM) |
| Connelly et al., 2013 [8] | Effectiveness of telemedicine interventions to promote PA on HbA1c | T2DM | significant positive effect on HbA1c: 4/8 studies (2x web-based, 2x mobile phone based) |
| de Jongh et al., 2012 [12] | Effectiveness of phone messaging applications to support DSME | Hypertension  Diabetes | significant positive effect on HbA1c: 4/4 studies  significant positive effect on TC: 1/1 study  significant positive effect on HDL-c: 1/1 study  significant positive effect on BP: 1/1 study |
| El-Gayar et al., 2013 [13] | Impact of Health Information Technologies on the self-management of diabetes | T1DM / T2DM | significant positive effect on HbA1c: 12/14 studies (T1DM)  significant positive effect on HbA1c: 22/23 studies (T2DM)  significant positive effect on HbA1c: 7/12 studies (T1DM/T2DM)  significant positive effect on FPG: 1/1 study (T1DM) |
| Farmer et al., 2016 [14] | Effectiveness of telemedicine strategies to increase adherence to medication and decrease HbA1c | T2DM | significant positive effect on HbA1c: 1/3 studies  significant positive effect on LDL-c: 1/3 studies  no significant positive effect on FPG: 1/3 studies |
| Fu et al., 2017 [16] | effectiveness of diabetes app use in T2DM | T2DM | significant positive effect on HbA1c: 6/10 studies (App only: 1x; App + web + decision support: 1x) |
| Holmen et al., 2017 [21] | Effectiveness of mobile interventions for tailored feedback by HCP in Diabetes | T1DM / T2DM | significant positive effect on HbA1c: 2/6 studies  significant positive effect on BP: 1/6 studies |
| Mushcab et al., 2015 [33] | Impact of web-based remote-monitoring solutions on HbA1c | T2DM | significant positive effect on HbA1c: 4/9 studies  significant positive effect on FPG: 3/9 studies  significant positive effect on total cholesterol: 2/9 studies  significant positive effect on LDL-c: 1/9 studies  significant positive effect on BP: 4/9 studies |
| Paré et al., 2010 [38] | Effectiveness of home telemonitoring in chronic disease | Diabetes  Asthma  Heart Failure  Hypertension | significant positive effect on SBP: 2/5 studies  significant positive effect on DBP: 2/5 studies |
| Porter et al., 2016 [40] | Clinical effects of home telemonitoring programs in patients with diabetes | T1DM / T2DM | significant positive effect on HbA1c: 0/3 studies (T1DM)  significant positive effect on HbA1c: 2/4 studies (T2DM)  significant positive effect on HbA1c: 1/2 studies (T1DM/T2DM)  significant positive effect on FPG: 2/9 studies (not specified) |
| Rush et al., 2018 [42] | Effectiveness of virtual education delivery in patients with diabetes | Diabetes | significant positive effect on HbA1c: 6/10 studies  significant positive effect on FPG: 1/10 studies  significant positive effect on post-prandial plasma glucose: 1/10 studies |
| Russell-Minda et al., 2009 [43] | Effectiveness of self-monitoring for patients with diabetes | T1DM | significant positive effect on HbA1c: 11/19 studies  significant positive effect on TC: 1/19 studies  significant positive effect on FPG: 2/19 studies  significant positive effect on Triglycerides: 1/19 studies  significant positive effect on DPB: 1/19 studies |
| Sun et al., 2018 [50] | Effectiveness of apps (with and without text messaging feature) for patients with T1DM | Diabetes | no significant positive effect on HbA1c: 5/5 studies |
| Vargas et al., 2017 [54] | Effectiveness of short messaging service (SMS) for hypertension management | Hypertension | Significant positive effect on BP: 3/6 studies  Significant positive effect on weight reduction: 2/3 studies  Significant positive effect BMI and waist circumference 2/2  Significant positive effect on medication adherence 1/2 studies  no significant positive effect on lipid levels: 1/1 study |

The table shows the characteristics (aims and target disease) and results of the 16 included systematic reviews
