# Supplementary material for: Mapping the Evidence on the Effectiveness of Telemedicine Interventions in Diabetes, Dyslipidemia, and Hypertension: An Umbrella Review of Systematic Reviews and Meta-Analyses
Source: J Med Internet Res. 2020 Mar 18;22(3):e16791. doi: 10.2196/16791 (PMC7113804; doi:10.2196/16791)
Supplement: Multimedia Appendix 7 [file jmir_v22i3e16791_app7.doc]

# Multimedia Appendix 8 - Results of included meta-analyses

Suppl. Table 1 Effectiveness of telemedicine (components) on HbA1c in patients with T1D / T2D (Table 5 in manuscript)

| **Category of application** | **Intervention characteristics** | **n of trials** | **n of patients** | **Follow-up in months** | **Outcome** | **MD (95% CI)** | **P** | **I^2^ in %** |
| --- | --- | --- | --- | --- | --- | --- | --- | --- |
| **Digital health education** [3] | *Intervention duration (3 months)* | 3 | 203 | 🡫 | | -0.71 (CI -1.00, -0.43) | = .9 | 0 |
|  | *Intervention duration (6 months)* | 2 | 562 | 🡫 | | -0.52 (CI -0.75,-0.29) | = .65 | 0 |
|  | *Intervention duration (12 months)* | 6 | 1153 | 🡫 | | -0.55 (CI -0.7,-0.39) | = .0005 | 78 |
|  | *Intervention duration (12 months) - without 2 multimodal intervention* | 4 | n.s. | 12 | 🡮 | -0.43 (CI -0.60, -0.26) | n.s. | 0 |
| **Digital self-management** [32, 39, 48, 49, 51, 59, 60] | **Overall** | 12 | 2647 | 🡮 | | -0.22 (CI -0.35, -0.08) | = .05 | 16 |
|  |  | 13 | 4207 | 🡮 | | -0.44 (CI -0.61, -0.26) | < .001 | 73 |
|  |  | 55 | 9258 | 🡮 | | -0.48 (CI -0.63, -0.33) | < .001 | n.s. |
|  |  | 42 | 6170 | 🡮 | | -0.37 (CI -0.43, -0.31) | < .001 | 98.6 |
|  |  | 16 | 6294 | 🡮 | | -0,22 (CI -0,28, -0,15) | < .001 | 46 |
|  |  | 33 | n.s. | 🡫 | | -0.57 (CI -0.71, -0.43) | ≤ .005 | 76,5 |
|  |  | 9 | 2553 | 🡮 | | -0.22 (CI -0.28, -0.15) | n.s. | 97.9 |
|  |  | 17 | n.s. | ≤ 6 | 🡫 | -0.66 (CI -1.13, -0.20) | < .001 | 97 |
|  |  | 25 | n.s. | > 6 | 🡫 | -2.25 (CI -2.76, 1.73) | < .001 | 95.7 |
|  | *Prescription through TM* | 4 | 334 | 🡫 | | -0.75 (CI -1.05, -0.43) | = .013 | n.s. |
|  | *No prescription through TM* | 7 | 3435 | 🡮 | | -0.30 (CI -0.45, -0.14) | = .013 | n.s. |
|  | *Physician intervention* | 3 | 847 | 🡫 | | -0.53 (CI -0.94, -0.13) | = .654 | n.s. |
|  | *Nurse intervention* | 9 | 3039 | 🡮 | | -0.43 (CI -0.64, -0.22) | = .654 | n.s. |
|  | *Teleconsultation component* | 18 | n.s. | 🡫 | | -0.62 | < .001 | n.s. |
|  | *Teleeducation component* | 17 | n.s. | 🡫 | | -0.64 (CI -0.85, -0.42) | < .001 | 83.05 |
|  | *Teleeducation component (mobile device)* | 21 | n.s. | 🡫 | | -0.67 (CI -0,90, -0,54) | < .001 | 80.9 |
|  | *Teleeducation component (SMS / Texting)* | 7 | n.s. | 🡫 | | -0.64 (CI -1,09, -0,19) | = .005 | 82.0 |
|  | *Teleeducation component (web-based)* | 6 | n.s. | 🡮 | | -0.48 (CI -0,65, -0,30) | < .001 | 44.15 |
|  | *Teleeducation component (other ICT)* | 8 | n.s. | 🡮 | | -0.19 (CI -0,34, -0,04) | = .0013 | 22.19 |
|  | *Intervention duration (≤ 6 months)* | 30 | n.s. | 🡫 | | -0.56 | < .001 | n.s. |
|  | *Intervention duration (6 months)* | 6 | 741 | 🡫 | | -0.57 (CI -0.85, -0.30) | = .099 | n.s. |
|  | *Intervention duration (> 6 months)* | 25 | n.s. | 🡮 | | -0.40 | < .001 | n.s. |
|  | *Intervention duration (12 months)* | 7 | 3466 | 🡮 | | -0.30 (CI -0.48, -0.11) | = .099 | n.s. |
|  | *Telephone-delivered intervention (phone calls)* | 5 | 953 | 🡮 | | - 0.38 (CI - 0.91, 0.16) | = .17 | 85 |
| **Digital self-management (mHealth)** [6, 10, 30, 57] | **Overall** | 22 | 2062 | 🡫 | | -0.51 (CI -0.69, -0.33) | n.s. | 72.6 |
|  |  | 12 | 974 | 🡮 | | -0,48 (CI -0.19, -0.78) | < .001 | 76 |
|  | *Remote access to usual care* | 7 | 680 | 🡫 | | -0.55 (CI -0.72,-0.38) | <.001 | 12 |
|  | *1 or 2 features* | 5 | 359 | 🡫 | | -0.52 (CI -0.76,-0.28) | <.001 | 41 |
|  | *3 or 4 features* | 8 | 901 | 🡮 | | -0.38 (CI -0.56,-0.2) | <.001 | 21 |
|  | *Self-management app* | 6 | 884 | 🡮 | | -0.40 (CI -0.69, -0.11) | = .007 | 77 |
|  | *Self-management app (2 studies excluded due to automated feedback)* | 4 | n.s. | 🡮 | | -0.17 (CI -0.32, -0.02) | = .03 | 2 |
|  | *General education* | 6 | n.s. | 🡫 | | -0.64 (CI -0.95, -0.33) | = .19 | 33 |
|  | *No general education* | 6 | n.s. | 🡮 | | -0.33 (CI -0.75, 0.09) | < .001 | 83 |
|  | *Complication prevention module* | 2 | n.s. | 🡫 | | -1.31 (CI -0.66, -1.96) | = .84 | 0 |
|  | *No complication prevention module* | 10 | n.s. | 🡮 | | -0.38 (CI -0.09, -0.68) | <.001 | 76 |
|  | *High risk interventions* | 3 | n.s. | 🡮 | | -0.19 (CI -0.63, 0.24) | = .004 | 82 |
|  | *Potential risk interventions* | 9 | n.s. | 🡫 | | -0.61 (CI -0.95, -0.27) | = .005 | 64 |
|  | *Personalised feedback* | 8 | n.s. | 🡮 | | -0.43 (CI -0.74, -0.12) | < .001 | 75 |
|  | *No personalised feedback* | 4 | n.s. | 🡫 | | -0.61 (CI -1.40, 0.19) | = .001 | 81 |
|  | *Frequency (daily)* | 15 | n.s. | 🡫 | | -0.6 (CI -0.9, -0.4) | = .27 | n.s. |
|  | *Frequency (weekly)* | 3 | n.s. | 🡮 | | -0.2 (CI -0.6, 0.2) | = .27 | n.s. |
|  | *Frequency (not specified)* | 4 | n.s. | 🡮 | | -0.4 (CI -0.5, -0.2) | = .27 | n.s. |
|  | *Structured display* | 8 | n.s. | 🡫 | | -0.69 (CI -0.32, -1.06) | = .008 | 63 |
|  | *No structured display* | 4 | n.s. | 🡮 | | -0.17 (CI -0.18, -0.53) | = .007 | 75 |
|  | *manual data entry* | 5 | n.s. | 🡫 | | -0.7 (CI -0.33, -1.07) | = .10 | 46 |
|  | *Education wireless data transport from device* | 6 | n.s. | 🡫 | | -0.53 (CI -0.15, -0.92) | = .001 | 86 |
|  | *Web + mobile* | 12 | n.s. | 🡫 | | -0.7 (CI -0.9, -0.4) | = .12 | n.s. |
|  | *Mobile only* | 10 | n.a. | 🡮 | | -0.4 (CI -0.6, -0.1) | = .12 | n.s. |
|  | *Intervention content (Other than SMBG or medication management)* | 9 | n.s. | 🡫 | | -0.5 (CI -07, -0.2) | = .6 | n.s. |
|  | *Medication management* | 13 | n.s. | 🡫 | | -0.6 (CI -0.8, -0.3) | = .6 | n.s. |
|  |  | 8 | n.s. | 🡫 | | -0.56 (CI -0.99, -0.13) | < .001 | 83 |
|  | *No medication management* | 4 | n.s. | 🡮 | | -0.42 (CI -0.71, -0.13) | = .23 | 30 |
|  | *Lifestyle modification management* | 11 | n.s. | 🡫 | | -0.52 (CI -0.84, -0.20) | < .001 | 78 |
|  | *No lifestyle modification management* | 1 | n.s. | 🡮 | | -0.10 (CI -0.67, 0.47) | n.s. | n.s. |
| **Digital self-management (SNS)** [53] | **Overall** | 34 | 4977 | 🡮 | | -0,45 (CI -0,6, -0,29) | < .001 | 81 |
|  | *Web-based only* | 15 | 1383 | 🡫 | | -0.51 (CI -0.68, -0.34) | < .001 | 82 |
|  | *Mobile only* | 3 | 371 | 🡮 | | -0.2 (-0.43, 0.03) | = .09 | 0 |
|  | *Web + mobile* | 16 | 3403 | 🡫 | | -0.54 (CI -0.72, -0.37) | < .001 | 71 |
|  | *Intervention duration (≤ 3 months)* | 13 | 799 | 🡫 | | -0.54 (CI -0.80, -0.28) | < .001 | 23 |
|  | *Intervention duration (3 -12 months)* | 11 | 1465 | 🡮 | | -0.41 (CI -0.63, -0.19) | < .001 | 25 |
|  | *Intervention duration (> 12 months)* | 10 | 2713 | 🡮 | | -0.36 (CI -0.59, -0.14) | < .002 | 90 |
| **Teleconsultation**  [15] | **Overall** | 39 | 3165 | ≤ 3 | 🡫 | -0.57 (CI −0.74, −0.40) | < .05 | 75 |
|  |  | 87 | 15524 | 4 – 12 | 🡮 | –0.28 (–0.37, –0.20) | < .05 | 69 |
|  |  | 5 | 1896 | > 12 | 🡮 | −0.26 (CI −0.46, –0.06) | < .05 | 58 |

n.s. = not specified by the authors of included studies

Suppl. Table 2 Effectiveness of telemedicine on HbA1c in patients with T1D / T2D according to according to population characteristics (age groups and baseline HbA1c) (Table 6 in Manuscript)

| **Category of application** | **Population characteristics** | **n of trials** | **n of patients** | **Outcome** | **MD (95% CI)** | **P** | **I^2^ in %** |
| --- | --- | --- | --- | --- | --- | --- | --- |
| **Digital self-management** [32, 48, 51, 59] | < 40 years | 14 | n.s. | 🡮 | -0.32 | = .024 | n.s. |
|  |  | 11 | n.s. | 🡫 | -0.85 (CI -1.79, 0.10) | = .07 | 98 |
|  | ≥ 40 years | 40 | n.s. | 🡫 | -0.53 | < .001 | n.s. |
|  | 41 to 50 years | 8 | n.s. | 🡫 | -1.83 (-3.17, -0.48) | < .001 | 96.2 |
|  | > 50 years | 17 | n.s. | 🡫 | -1.05 (CI -1.50, -0.60) | < .001 | 97 |
|  | Baseline HbA1c <8 % | n.s. | n.s. | 🡮 | -0.26 (CI -0.43, -0.10) | = .027 | n.s. |
|  | Baseline HbA1c ≥8 % | n.s. | n.s. | 🡫 | -0.64 (CI -0.93, -0.35) | = .027 | n.s. |
|  | Baseline HbA1c < 9.0% | n.s. | n.s. | 🡮 | -0.35 | n.s. | n.s. |
|  | Baseline HbA1c ≥ 9.0% | n.s. | n.s. | 🡫 | -1.22 | n.s. | n.s. |
| **Diabetes self-management (mHealth)** [30] | Average age < 25 years | 5 | n.s. | 🡫 | -0.5 (CI -0.8, -0.1) | = .54 | n.s. |
|  | Average age ≥ 25 years | 17 | n.s. | 🡫 | -0.5 (CI -0.7, -0.3) | = .54 | n.s. |
|  | BMI ≥ 25 | 7 | n.s. | 🡫 | -0.8 (CI -1.1, -0.5) | = .93 | n.s. |
|  | 24 ≤ BMI < 25 | 3 | n.s. | 🡫 | -0.8 (CI -1.7, 0.1) | = .93 | n.s. |
|  | BMI unspecified | 12 | n.s. | 🡮 | -0.3 (CI -0.5, -0.1) | = .93 | n.s. |

n.s. = not specified by the authors of included studies

Suppl. Table 3 Effectiveness of telemedicine (components) on HbA1c in patients with T2D (Table 7 in Manuscript)

| **Category of application** | **Other intervention characteristics** | **n of trials** | **n of patients** | **follow-up in months** | **Outcome** | **MD (95% CI)** | **P** | **I^2^ in %** |
| --- | --- | --- | --- | --- | --- | --- | --- | --- |
| **Telemedicine** [28] | **Overall** | 93 | 16,791 | 🡮 | | -0.43 (CI -0.64, -0.21) | < .001 | 99.9 |
|  | Teleeducation | 26 | 4211 | 🡮 | | -0.36 (CI -0.97, -0.07) | < .001 | 81 |
|  | Teleconsultation | 7 | 1328 | 🡫 | | -0.64 (CI -3.74, -0.02) | < .001 | 98 |
|  | Telecasemanagement | 8 | 1620 | 🡮 | | -0.28 (CI -2.87, 0.13) | < .001 | 97 |
|  | Teleeducation + telemonitoring | 8 | 1540 | 🡮 | | -0.35 (CI -2.20, -0.02) | < .001 | 72 |
|  | Telecasemanagement + telemonitoring | 9 | 1194 | 🡫 | | -0.54 (CI -2.44, -0.06) | < .001 | 84 |
|  | Teleeducation + Telecasemanagement | 9 | 1409 | 🡮 | | -0.31 (CI -2.66, -0.02) | < .001 | 96 |
|  | Telecasemanagement + teleconsultation | 1 | 40 | 🡫 | | -1.20 (CI -2.30, -0.10) | < .001 | n.s. |
|  | *Intervention duration (* *≤3 months)* | 17 | 1377 | 🡫 | | −0.67 [CI −0.93, −0.41] | n.s. | n.s. |
|  | *Intervention duration (4-6 months)* | 36 | 4538 | 🡮 | | −0.41 (CI −0.84, 0.02) | n.s. | n.s. |
|  | *Intervention duration (7-11 months)* | 4 | 659 | 🡫 | | -0.66 (CI -1.18, -0.15) | n.s. | n.s. |
|  | *Intervention duration (≥ 12 months)* | 36 | 10237 | 🡮 | | −0.26 (CI −0.40, −0.12) | n.s. | n.s. |
| **Digital self-management** [2, 24, 25, 32, 37, 45, 48, 51, 58, 61] | **Overall** | 21 | n.s. | 🡮 | | -0.48 (CI -0.63, -0.32) | n.s. | 99 |
|  |  | 35 | n.s. | 🡮 | | -0.37 (CI -0.49, -0.25) | < .001 | 75.5 |
|  |  | 21 | 3787 | 🡮 | | -0.39 (CI -0.51, -0.26) | < .05 | 80.8 |
|  |  | 17 | 2225 | 🡫 | | -0.51 (CI -0.71, -0.30) | <.001 | 47 |
|  |  | 17 | n.s. | 🡫 | | -0.5 (CI -0.67,-0.43) | < .001 | 62.1 |
|  |  | 31 | n.s. | 🡫 | | -0.63 | < .001 | n.s. |
|  |  | 18 | 3798 | 🡫 | | -0.54 (CI -0.75, -0.34) | < .05 | 76 |
|  |  | 8 | n.s. | 3 | 🡫 | -0.73 (CI-0.99, -0.47) | = .11 | 46 |
|  |  | 5 | n.s. | ≤ 6 | 🡮 | -0.3 (CI -0.6 , -0.1) | = .14 | 43 |
|  |  | 11 | 981 |  | 🡫 | -0.53 (-0.79, -0.27) | = .77 | 52 |
|  |  | 7 | n.s. | 6 | 🡫 | -0.53 (CI -0.71, -0.34) | = .11 | 17 |
|  |  | 6 | 550 | > 6 | 🡮 | -0.46 (CI -0.85, -0.07) | = .77 | 47 |
|  |  | 6 | n.s. |  | 🡮 | -0.1 (CI -0.3, 0.1) | = .02 | 61 |
|  |  | 2 | n.s. | 9 | 🡫 | -0.92 (CI -1.44, -0.40) | = .11 | 31 |
|  |  | 6 | n.s. | 12 | 🡮 | -0.29 (CI -0.56, -0.02) | = .11 | 67 |
|  |  | 1 | n.s. | 15 | 🡫 | -0.50 (CI -1.06, 0.06) | n.s. | n.s. |
|  | *Telehealth* | 5 | 670 | 🡮 | | -0.21 (CI -0.65, 0.22) | n.s. | 87.3 |
|  | Web-based education | 14 | n.s. | 🡫 | | –0.51 (CI –0.69, –0.32) | < .001 | 66.9 |
|  | No web-based education | 20 | n.s. | 🡮 | | –0.36 (CI –0.51, –0.22) | < .001 | 53.6 |
|  | *Computer-based* | 11 | 2637 | 🡮 | | -0.21 (CI -0.37, -0.05) | = .001 | 58 |
|  | *Mobile-phone based* | 8 | n.s. | 🡮 | | –0.31 (CI –0.49, –0.14) | < .001 | 27.2 |
|  |  | 3 | 280 | 🡫 | | -0.5 (CI -0.7, 0.3) | = .021 | 0 |
|  | *Web-based* | 35 | 6475 | 🡮 | | –0.43 (CI –0.54, –0.31) | < .001 | 60.1 |
|  |  | 8 | n.s. | 🡮 | | –0.48 (CI –0.71, –0.24) | < .001 | 57 |
|  | *Mobile + web-based* | 9 | n.s. | 🡫 | | –0.77 (CI –1.07, –0.47) | < .001 | 64.6 |
|  | *Homebased* | 4 | n.s. | 🡮 | | -0.3 (CI -0.5, -0.04) | = .021 | 47 |
|  | *Feedback (human call / telephone)* | 5 | n.s. | 🡫 | | -1.13 (CI -1.51, -0.75) | < .05 | 38 |
|  |  | 12 | n.s. | 🡫 | | -0.53, (CI -0.81, -0.26) | < .001 | 76.35 |
|  | *Feedback (manual)* | 6 | 1180 | 🡮 | | -0.44 (CI -0.74, -0.15) | = .039 | n.s. |
|  |  | 22 | n.s. | 🡫 | | –0.50 (CI –0.65, –0.34) | < .001 | 67.2 |
|  | *Feedback (automated)* | 5 | n.s. | 🡫 | | –0.50 (CI –0.69, –0.32) | < .001 | 0 |
|  | *Feedback (automated calls)* | 2 | n.s. | 🡮 | | -0.01 (CI -0.32, 0.29) | = .94 | 0 |
|  | *Feedback (automated text)* | 9 | n.s. | 🡮 | | -0.36 (CI -0.47, -0.24) | n.s. | 0 |
|  | *Feedback (text message)* | 3 | 380 | 🡫 | | -0.52 (CI -1.04 to 0.00) | < .05 | 73.5 |
|  | *Feedback (web-based / internet transmitted)* | 13 | 2405 | 🡮 | | -0.41 (CI -0.55, -0.27) | < .05 | 79.6 |
|  |  | 19 | n.s. | 🡫 | | -0.62 (CI -0.82, -0.42) | < .001 | 77.57 |
|  |  | 4 | n.s. | 🡮 | | -0.12 (CI -0.29, 0.06) | = .201 | 0 |
|  | *Tailoring* | 19 | n.s. | 🡮 | | -0.36 (CI -0.49, -0.22) | < .001 | 81.6 |
|  | *No tailoring* | 4 | n.s. | 🡫 | | -0.56 (CI -0.87, -0.24) | = .001 | 60.5 |
|  | *Electronic decision support system* | 7 | n.s. | 🡮 | | -0.15 (CI -0.34,-0.16) | < .001 | 89.8 |
|  | *High smartphone technology exposure* | 7 | 667 | 🡮 | | -0.36 (CI -0.58, -0.14) | = .06 | 0 |
|  | *Low smartphone technology exposure* | 4 | 365 | 🡮 | | -0.34 (CI -0.73, 0.05) | = .06 | 44 |
|  | *Intervention duration (≤ 3 months)* | 10 | n.s. | 🡫 | | –0.51 (CI –0.71, –0.31) | < .001 | 41.8 |
|  | *Intervention duration (> 3 ≤ 6months)* | 10 | n.s. | 🡮 | | –0.48 (CI –0.68, –0.28) | < .001 | 34.5 |
|  | *Intervention duration (3 -4 months)* | 11 | 1613 | 🡮 | | -0.30 (CI -0.495, -0.11) | = .000 | 89.1 |
|  | *Intervention duration (> 6 months)* | 15 | n.s. | 🡮 | | –0.35 (CI –0.53, –0.18) | < .001 | 70.5 |
|  | *Intervention duration (6 -8 months)* | 14 | 2389 | 🡫 | | -0.59 (CI -0.78, -0.39) | < .001 | 84.8 |
|  | *Intervention duration (9 -12 months)* | 7 | 1272 | 🡮 | | -0.21 (CI -0.35, -0.075) | = .131 | 39.1 |
| **Digital self-management (mHealth)** [10, 23, 30, 57] | **Overall** | 10 | n.s. | 🡫 | | -0.81 (CI -1.11, -0.50) | n.s. | 73.2 |
|  |  | 5 | n.s. | 🡫 | | -0.67 (CI -0.30, -1.03) | = .11 | 47 |
|  |  | 6 | 884 | 🡮 | | -0.40 (CI -0.69, -0.11) | = .007 | 77 |
|  | *Feedback (low frequency)* | 7 | 440 | 🡮 | | -0.33 (CI -0.59, -0.07) | = .01 | 47.35 |
|  | *Feedback (high frequency)* | 5 | 326 | 🡫 | | -1.12 (CI -1.32, -0-91) | < .001 | 0 |
| **Digital self-management (SMS)** [44] | **Overall** | 10 | 960 | 🡫 | | -0.60 (CI -0.83, -0.36) | < .001 | 67.6 |
|  | *SMS (interactive)* | 6 | n.s. | 🡫 | | -0.60 (CI -0.76, -0.44) | < .001 | n.s. |
|  | *SMS (unidirectional)* | 4 | n.s. | 🡮 | | -0.31 (CI -0.51, -0.12) | < .001 | n.s. |
|  | *Communication tool: SMS only* | 6 | n.s. | 🡮 | | -0.44 (CI -0.68, -0.29) | = .01 | n.s. |
|  | *Communication tool: both SMS + web* | 4 | n.s. | 🡫 | | -0.87 (CI -1.44, -0.20) | = .003 | n.s. |
|  | *Intervention duration < 6 months* | 6 | n.s. | 🡫 | | -0.604 (CI -0.80, -0.40) | < .001 | n.s. |
|  | *Intervention duration ≥ 6months* | 4 | n.s. | 🡮 | | -0.40 (CI -0.56, -0.24) | < .001 | n.s. |
| **Digital self-management (SNS)** [46, 53] | **Overall** | 21 | 2410 | 🡫 | | -0.55 (CI -0.68, -0.42) | <.001 | 64 |
|  |  | 9 | 1081 | 3 | 🡮 | -0.25 (CI -0.40, -0.11) | = .001 | 12 |
|  |  | 11 | 1504 | 6 | 🡮 | -0.24 (CI -0.52, 0.03) | = 0.08 | 83 |
|  |  | 3 | 674 | > 7 | 🡮 | -0.10 (CI -0.84, 0.64) | = 0.8 | 99 |

n.s. = not specified by the authors of included studies

Suppl. Table 4 Effectiveness of telemedicine on HbA1c in patients with T2D according to population characteristics (Table 8 in Manuscript)

| **Category of application** | **Population characteristics** | **n of trials** | **n of patients** | **Outcome** | **MD (95% CI)** | **P** | **I^2^ in %** |
| --- | --- | --- | --- | --- | --- | --- | --- |
| **Telemedicine** [28] | Baseline HbA1c < 8.0 % | 48 | 5720 | 🡮 | -0.22 (CI -0.25 to -0.19) | n.s. | n.s. |
|  | Baseline HbA1c ≥ 8.0% | 45 | 8100 | 🡫 | -0.60 (CI -0.61 to -0.60) | n.s. | n.s. |
| **Digital self-management** [24, 25, 51, 58] | < 55 years | 7 | 701 | 🡫 | -0.67 (CI -1.15, -0.20) | = .52 | 75 |
|  | ≥55 years | 8 | 541 | 🡮 | -0.41 ( -0.62, -0.21) | = .52 | 0 |
|  | Age undetermined | 2 | 289 | 🡫 | -0.72 (CI -1.60, 0.16) | = .52 | 47 |
|  | Diagnosis < 8.5 years ago | 7 | 549 | 🡫 | -0.83 (CI -1.10, 0.56) | = .007 | 0 |
|  | Diagnosis ≥8.5 years ago | 4 | 394 | 🡮 | -0.22 (CI -0.44, 0.01) | = .007 | 0 |
|  | Diagnosis time undetermined | 6 | 588 | 🡮 | -0.43 (-0.71, -0.30) | = .007 | 55 |
|  | Baseline HbA1c ≤ 8.0 % | 6 | 590 | 🡮 | -0.49 (CI -0.71, -0.27) | = .69 | 0 |
|  |  | 7 | n.s. | 🡮 | -0.33 (CI -0.53, -0.13) | < .05 | 46 |
|  | Baseline HbA1c > 7.0 % | 11 | 1707 | 🡮 | -0.33 (CI -0.48, -0.18) | < .001 | 77.8 |
|  | Baseline HbA1c > 7.5% | 10 | 1921 | 🡮 | -0.45 {CI -0.70, -0.21) | < .001 | 80.4 |
|  | Baseline HbA1c > 8.0% | 11 | 941 | 🡫 | -0.57 (CI -0.93, -0.22) | = .69 | 65 |
|  |  | 11 | n.s. | 🡫 | -0.70 (CI -1.03, -0.36) | < .05 | 81 |
|  | Baseline BMI < 30 | 5 | 359 | 🡫 | -0.64 (CI -0.91, -0.36) | = .49 | 0 |
|  | Baseline BMI ≥ 30 | 10 | 966 | 🡮 | -0.43 (CI -0.68, -0.17) | = .49 | 35 |
|  | Baseline BMI undetermined | 2 | 206 | 🡫 | -0.96 (CI -2.76, 0.85) | = .49 | 91 |
| **Digital self-management (mHealth)**  [10] | Baseline HbA1c < 8% | 4 | 696 | 🡮 | -0.33 (CI -0.59, -0.06) | = .02 | 70 |
| **Digital self-management (SMS)** [44] | < 55 years | 5 | n.s. | 🡫 | -0.65 (CI -0.88, -0.41) | < .001 | n.s. |
|  | ≥55 years | 5 | n.s. | 🡮 | -0.42 (CI -0.56, -0.27) | = .006 | n.s. |
|  | Diagnosis < 7 years ago | 4 | n.s. | 🡫 | -0.61 (CI -0.79, -0.42) | < .001 | n.s. |
|  | Diagnosis ≥ 7 years ago | 3 | n.s. | 🡮 | -0.37 (CI -0.62, -0.13) | = .031 | n.s. |
|  | Baseline HbA1c < 8.0 % | 5 | n.s. | 🡫 | -0.71 (CI -0.93, -0.48) | < .001 | n.s. |
|  | Baseline HbA1c ≥ 8.0% | 5 | n.s. | 🡫 | -0.38 (CI -0.533, -0.24) | < .001 | n.s. |

n.s. = not specified by the authors of included studies

Suppl. Table 5 Effect of telemedicine on HbA1c in patients with T1D (Table 9 in Manuscript)

| **Category of application** | **Intervention characteristics** | **n of trials** | **n of patients** | **Follow up in months** | **Outcome** | **MD (95% CI)** | **P** | **I^2^ in %** |
| --- | --- | --- | --- | --- | --- | --- | --- | --- |
| **Telemedicine** [23, 29, 30, 48, 51, 53, 57] | **Overall** | 9 | n.s. |  | 🡮 | -0.27 (CI-0.54, -0.01) | n.s. | 67.5 |
|  |  | 12 | n.s. |  | 🡮 | -0.26 (CI -0.49, -0.04) | n.s. | 98.9 |
|  |  | 7 | 498 |  | 🡮 | -0.12 (CI -0.32, 0.08) | = .26 | 0 |
|  |  | 15 | n.s. |  | 🡮 | -0.27 | = .027 | n.s. |
|  |  | 28 | 2099 |  | 🡮 | -0.18 (CI -0.33, -0.04) | = .01 | 66.1 |
|  |  | 5 | n.s. |  | 🡮 | -0.37 (CI -0.12, -0.86) | <.001 | 86 |
|  |  | 2 | 145 |  | 🡫 | -0.86 (CI -1.12, -0.59) | = .039 | n.s. |
|  |  | 2 | 143 | 3 | 🡮 | -0.50 (CI -1.89, 0.89) | = .48 | 89 |
|  |  | 2 | 85 | 6 | 🡭 | 0.15 (CI -0.54, 0.84) | = .67 | 84 |
|  | Teleconsultation | 2 | n.s. | 🡭 | | 0.16 (CI -0.30, 0.62) | n.s. | 21.8 |
|  | Telecasemanagement | 1 | n.s. | 🡮 | | -0.48 (CI -1.09, 0.13) | n.s. | n.s. |
|  | Teleeducation | 4 | n.s. | 🡮 | | -0.23 (CI -0.58, 0.13) | n.s. | 84.2 |
|  | Teleeducation + teleconsultation | 1 | n.s. | 🡫 | | -0.91 (CI -1.21, -0.61) | n.s. | n.s. |
|  | App-based feedback | 5 | 336 | 🡮 | | -0.37 (CI -0.94, 0.20) | = .20 | 81.74 |
|  | High intensity (direct contact at least once a week) | 13 | n.s. | 🡮 | | -0.24 (CI -0.49, 0.01) | n.s. | n.s. |
|  | No high intensity | 14 | n.s. | 🡮 | | -0.09 (-0.23, 0.06) | n.s. | n.s. |
|  | Intervention duration ≥ 6 months | 21 | n.s. | 🡮 | | -0.24 (CI -0.41, -0.07) | n.s. | n.s. |
|  | Intervention duration < 6 months | 7 | n.s. | 🡭 | | 0.07 (CI -0.16, 0.31) | n.s. | n.s. |
|  | Multi component (≥ 2 components) | 12 | n.s. | 🡮 | | -0.32 (CI -0.55, -0.08) | n.s. | n.s. |
|  | No multi-component intervention (< 3 components) | 16 | n.s. | 🡮 | | -0.05 (C)I -0.24, 0.15) | n.s. | n.s.. |
|  | Individualised assessment included | 13 | n.s. | 🡮 | | -0.27 (CI -0.49, -0.04) | n.s. | n.s. |
|  | No individualised assessment | 15 | n.s. | 🡮 | | -0.10 (CI -0.31, -0.11) | n.s. | n.s. |
|  | Audit & feedback | 24 | n.s. | 🡮 | | -0.22 (CI -0.38, -0.06) | n.s. | n.s. |
|  | No audit & feedback | 4 | n.s | 🡮 | | 0.01 (CI -0.27, -0.30) | n.s. | n.s. |
|  | **Population characteristics** | **n of trials** | **n of patients** | **Outcome** | | **MD (95% CI)** | **P** | **I^2^ in %** |
|  | Adults | 15 | 1256 | 🡮 | | -0.26 (CI -0.47 to -0.05) | < .01 | 79.7 |
|  | Children and adolescents | 11 | 796 | 🡮 | | -0.12 (CI -0.30, 0.05) | = .70 | 0 |
|  | Median baseline < 9.0% | 16 | n.s. | 🡮 | | -0.06 (CI -0.02, 0.09) | n.s. | n.s. |
|  | Median baseline ≥ 9.0% | 12 | n.s. | 🡮 | | -0.34 (CI -0.57, -0.11) | n.s. | n.s. |
