# Supplementary material for: Mapping the Evidence on the Effectiveness of Telemedicine Interventions in Diabetes, Dyslipidemia, and Hypertension: An Umbrella Review of Systematic Reviews and Meta-Analyses
Source: J Med Internet Res. 2020 Mar 18;22(3):e16791. doi: 10.2196/16791 (PMC7113804; doi:10.2196/16791)
Supplement: Multimedia Appendix 8 [file jmir_v22i3e16791_app8.doc]

# Multimedia Appendix 9 - Grading of Recommendations Assessment, Development and Evaluation of glycated haemoglobin and diastolic blood pressure/systolic blood pressure outcomes

Suppl. Table 1 Assessment of subgroup-specific certainty of outcomes (HbA1c) using GRADE

| **Author, Year** | **Subgroups** | | **Number of included Studies** | **Certainty Assessment** | | | | | **Clinical relevance** | **Certainty** |
| --- | --- | --- | --- | --- | --- | --- | --- | --- | --- | --- |
|  |  |  |  | **Risk of Bias** | **Inconsistency** | **Indirectness** | **Imprecision** | **Publication bias** |  |  |
| [2] | Intervention type | Electronic self-management system | 17 | -1  Moderate to high quality score | -2  High heterogeneity | -2  Differences in populations (T1 or T2, age per eligibility), intervention (education / or communication with clinician, tool used), setting | -2  Large CIs | -2  Not available, Only US studies | 1 | + |
|  |  | Electronic decision support system | 7 | -1  Moderate to high quality score | -2  High heterogeneity | -2  Differences in populations (T1 or T2, age per eligibility), intervention (education / or communication with clinician, tool used | 0 | -2  Not available | 0 | + |
| [3] | Intervention duration | 3 months | 3 | -2  Unknown Risk of AC, high risk of unblinding | 0 | 0 | -1 | 0 | 1 | +++ |
|  |  | 6 months | 2 | -2  Unknown Risk of AC, high risk of unblinding | 0 | -1  Differences in interventions (computer-based vs. cell-phone-based) | -1 | 0 | 1 | ++ |
|  |  | 12 months | 6 | -2  Unknown Risk of AC, high risk of unblinding | -2  High heterogeneity, CIs inconsistant | -2  Differences in populations (T1 or T2, age per eligibility), intervention (computer-based vs. cell-phone-based) | -1 | 0 | 1 | + |
| [6] | overall | | 13 | -1  High risk of Unblinding | -1  CIs overlap | -2 | -1 | -2  Not available | 0 | + |
|  | Intervention type | remote access to usual care | 7 | -1  High risk of Unblinding | -2  CIs overlap | -2  Differences in population (age, diabetes type), Varying intervention duration, varying intervention features | -1 | -2  Not available | 1 | + |
|  |  | 1 or 2 features | 5 | -1  High risk of Unblinding | -1  CIs overlap | -2  Differences in population (age, diabetes type), Varying intervention duration, varying intervention features | -1 | -2  Not available | 1 | + |
|  |  | 3 or 4 features | 8 | -1  High risk of Unblinding | -2  CIs overlap | -2  Differences in population (age, diabetes type), Varying intervention duration, varying intervention features | -2 | -2  Not available | 0 | + |
| [10] | Intervention type | Self-management app | 6 | -2  unclear risk of Unblinding and reporting bias | -2  High heterogeneity, CIs overlap | -1  Differences in populations (baseline HbA1c) interventions (components, feedback delivery), intervention duration | -1 | -2  Not available | 0 | ++ |
|  |  | Self-management app (2 studies excluded due to automated feedback) | 4 | -2  unclear risk of Unblinding and reporting bias | -1  CIs overlap | 0 | -1 | -2  Not available | 0 | ++ |
|  | Population characteristics | Baseline HbA1c < 8% | 4 | -2  unclear risk of Unblinding and reporting bias | -2  High heterogeneity, CIs overlap | -1  Differences in interventions (components, feedback delivery), intervention duration | -1 | -2  Not available | 0 | + |
| [15] | Follow-up in months | ≤ 3 | 39 | -2  Unclear risk of AC and Blinding, high risk of Selec. Bias and ITT | -2  High heterogeneity, CIs overlap | -2  Not available (overall: differences in populations (diabetes type, age, duration of diabetes), interventions (form of communication, frequency of feedback, provider involved) | -2  Not available | -1  3 outliers | 1 | + |
|  |  | 4 – 12 | 87 | -2  Unclear risk of AC and Blinding, high risk of Selec. Bias and ITT | -2  High heterogeneity, CIs overlap | -2  differences in populations (diabetes type, age, duration of diabetes), interventions (form of communication, frequency of feedback, provider involved) | -2  Large CIs | -1  3 outliers | 0 | + |
|  |  | > 12 | 5 | -2  Unclear risk of AC and Blinding, high risk of Selec. Bias and ITT | -1  moderate heterogeneity, CIs overlap | -2  Not available (overall: differences in populations (diabetes type, age, duration of diabetes), interventions (form of communication, frequency of feedback, provider involved) | -2  Not available | -1  3 outliers | 0 | + |
| [23] | Intervention characteristics | App-based feedback; T1D | 5 | -2  Unclear risk of AC, high risk of Unblinding, Detect, attrition bias and reporting bias | -2  High heterogeneity | -2  Differences in interventions (components, feedback delivery), intervention duration | -1 | -2  No stat. test applied | 0 | + |
|  |  | Feedback (low frequency); T2D | 7 | -2  Unclear risk of AC, high risk of Unblinding, Detect, attrition bias and reporting bias | -1  moderate heterogeneity, CIs overlap | -2  Differences in interventions (components, feedback delivery), intervention duration | -1 | -2  No stat. test applied | 0 | + |
|  |  | Feedback (high frequency); T2D | 5 | -2  Unclear risk of AC, high risk of Unblinding, Detect, attrition bias and reporting bias | 0 | -1  Differences in interventions (components, feedback delivery), intervention duration | 0 | -2  No stat. test applied | 1 | ++ |
| [24] | Overall | | 18 | -2  (LoAC, LoB, LTFU) | -2  High heterogeneity, CIs overlap | -2  Differences in interventions (telecare method, control group) and study location, Baseline HbA1c and age unclear | -1 | -2 | 1 | + |
|  | Follow-up | 3 | 8 | -2  (LoAC, LoB, LTFU) | -1  moderate heterogeneity, CIs inconsistant | -2  Differences in interventions (telecare method, control group) and study location, Baseline HbA1c and age unclear | -1 | -2 | 0 | + |
|  |  | 6 | 7 | -2  (LoAC, LoB, LTFU) | 0 | -2  Differences in interventions (telecare method, control group) and study location, Baseline HbA1c and age unclear | -2  Not available | -2 | 0 | + |
|  |  | 9 | 2 | -2  (LoAC, LoB, LTFU) | 0 | -2  Not available | -2  Not available | -2 | 0 | + |
|  |  | 12 | 6 | -1  (LoAC, LoB, LTFU) | -1  moderate heterogeneity, CIs overlap | -2  Differences in interventions (telecare method, control group) and study location, Baseline HbA1c and age unclear | 0 | -2 | 0 | + |
|  |  | 15 | 1 | - | - | - | - | - | - | Only 1 trial |
|  | Population characteristics | Baseline HbA1c ≤ 8.0 % | 7 | -2  Not available | -1  Cis unknown | -2  Not available | -2  Not available | -2 | 0 | + |
|  |  | Baseline HbA1c > 8.0% | 11 | -2  Not available | -2  High heterogeneity | -2  Not available | -2  Not available | -2 | 1 | + |
|  | Intervention characteristics (Feedback ways) | Human calls | 5 | -2  Not available | -1  Cis unknown | -2  Not available | -2  Not available | -2 | 1 | + |
|  |  | Automated calls | 2 | -2  Not available | -1  Cis unknown | -2  Not available | -2  Not available | -2 | 0 | + |
|  |  | Automated text | 9 | -2  Not available | -1  Cis unknown | -2  Not available | -2  Not available | -2 | 0 | + |
| [25] | Overall (T2D) | | 21 | -2  (LoAC, LoB, LTFU) | -2  High heterogeneity, CIs overlap | -2  Differences in intervention (telemonitoring/ videoconferencing/ online disease management, involvement of professional, content either on healthy eating or medication monitoring), populations (age, diabetes duration and Baseline HbA1c unclear) | -2  Large CIs | -1 | 0 | + |
|  | Intervention characteristics | Feedback (text message) | 3 | -1  (LoAC, LoB, LTFU) | -2  High heterogeneity, CIs overlap | -1  Slight differences in intervention (tailoring), populations (underserved, Hispanics; age and Baseline HbA1c unclear) | -2  Large CIs | -1 | 1 | + |
|  |  | Feedback (web-based) | 13 | -2  (LoAC, LoB, LTFU) | -2  High heterogeneity, CIs overlap | -2  Slight differences in intervention (involvement of professional, content either on healthy eating or medication monitoring), populations (underserved, Hispanics; age and Baseline HbA1c unclear) | -1  Large CIs | -1 | 0 | + |
|  |  | Telehealth | 5 | -2  (LoAC, LoB, LTFU) | -2  High heterogeneity, CIs overlap | -1  Slight differences in intervention (involvement of professional, content either on healthy eating or medication monitoring), populations (underserved, Hispanics; age and Baseline HbA1c unclear) | -2  Large CIs | -1 | 0 | + |
|  |  | Tailoring | 19 | -2  (LoAC, LoB, LTFU) | -2  High heterogeneity | -2  Slight differences in intervention (telemonitoring/ videoconferencing/ online disease management, involvement of professional, content either on healthy eating or medication monitoring), populations (age, diabetes duration and Baseline HbA1c unclear) | -2  Large CIs | -1 | 0 | + |
|  |  | No tailoring | 4 | -2  (LoAC, LoB, LTFU) | -2  High heterogeneity | -2  Slight differences in intervention (telemonitoring/ videoconferencing/ online disease management, involvement of professional, content either on healthy eating or medication monitoring), populations (age, diabetes duration and Baseline HbA1c unclear) | -2  Large Cis | -1 | 1 | + |
|  |  | Use of theory to guide intervention designs | 10 | -2  (LoAC, LoB, LTFU) | -1  Cis overlap | -2  Slight differences in intervention (telemonitoring/ videoconferencing/ online disease management, involvement of professional, content either on healthy eating or medication monitoring), populations (age, diabetes duration and Baseline HbA1c unclear) | -1  Large Cis | -1 | 0 | ++ |
|  |  | No use of theory to guide intervention designs | 13 | -2  (LoAC, LoB, LTFU) | -2  High heterogeneity | -2  Slight differences in intervention (telemonitoring/ videoconferencing/ online disease management, involvement of professional, content either on healthy eating or medication monitoring), populations (age, diabetes duration and Baseline HbA1c unclear) | -2  Large Cis | -1 | 1 | + |
|  | Intervention duration | 3-4 months | 11 | -2  (LoAC, LoB, LTFU) | -2  High heterogeneity, CIs overlap | -2  Slight differences in intervention (telemonitoring/ videoconferencing/ online disease management, involvement of professional, content either on healthy eating or medication monitoring, tailoring), populations (age, diabetes duration and Baseline HbA1c unclear) | -2  Large CIs | -1 | 0 | + |
|  |  | 6-8 months | 14 | -2  (LoAC, LoB, LTFU) | -2  High heterogeneity, CIs overlap | -2  Slight differences in intervention (telemonitoring/ videoconferencing/ online disease management, involvement of professional, content either on healthy eating or medication monitoring, tailoring), populations (age, diabetes duration and Baseline HbA1c unclear) | -2  Large CIs | -1 | 0 | + |
|  |  | 9-12 months | 7 | -2  (LoAC, LoB, LTFU) | -1  Moderate heterogeneity, CIs overlap | -2  Slight differences in intervention (telemonitoring/ videoconferencing/ online disease management, involvement of professional, content either on healthy eating or medication monitoring, tailoring), populations (age, diabetes duration and Baseline HbA1c unclear) | -1  Large CIs | -1 | 0 | ++ |
|  | Population characteristics | Baseline HbA1c > 7.0 % | 11 | -2  (LoAC, LoB, LTFU) | -2  High heterogeneity, CIs overlap | -2  Slight differences in intervention (telemonitoring/ videoconferencing/ online disease management, involvement of professional, content either on healthy eating or medication monitoring, tailoring), populations (age, diabetes duration unclear) | -1  Large CIs | -1 | 0 | + |
|  |  | Baseline HbA1c > 7.5 % | 10 | -2  (LoAC, LoB, LTFU) | -2  High heterogeneity, CIs overlap | -2  Slight differences in intervention (telemonitoring/ videoconferencing/ online disease management, involvement of professional, content either on healthy eating or medication monitoring, tailoring), populations (age, diabetes duration unclear) | -1  Large CIs | -1 | 0 | + |
| [28] | Overall (T2) | | 93 | -2 | -2  High Heterogeneity | -2  Differences in intervention (components, educational contents, Frequency of intervention duration, professional involved) | -2  Not available | -1 | 0 | + |
|  | Population characteristics | Baseline HbA1c < 8.0 % | 48 | -2 | -2  Not available | -2  Not available | -2  Not available | -1 | 0 | + |
|  |  | Baseline HbA1c ≥ 8.0 % | 45 | -2 | -2  Not available | -2  Not available | -2  Not available | -1 | 0 | + |
|  | Intervention duration | ≤ 3 months | 17 | -2 | -2  Not available | -2  Differences in intervention (components, educational contents, Frequency of intervention duration, professional involved) | -2  Not available | -1 | 1 | + |
|  |  | 4 - 6 months | 36 | -2 | -2  Not available | -2  Differences in intervention (components, educational contents, Frequency of intervention duration, professional involved) | -2  Not available | -1 | 0 | + |
|  |  | 7-11 months | 4 | -2 | -2  Not available | -2  Differences in intervention (components, educational contents, Frequency of intervention duration, professional involved) | -2  Not available | -1 | 1 | + |
|  |  | ≥ 12 months | 36 | -2 | -2  Not available | -2  Differences in intervention (components, educational contents, Frequency of intervention duration, professional involved) | -2  Not available | -1 | 0 | + |
|  | Intervention characteristics | Teleeducation | 26 | -2 | -2  High Heterogeneity | -2  Differences in intervention (components, educational contents, Frequency of intervention duration, professional involved) | -2  Not available | -1 | 0 | + |
|  |  | Teleconsultation | 7 | -2 | -2  High Heterogeneity | -2  Differences in intervention components, frequency and duration | -2  Not available | -1 | 1 | + |
|  |  | Telecasemanagement | 8 | -2 | -2  High Heterogeneity | -2  Differences in intervention components, frequency and duration | -2  Not available | -1 | 0 | + |
|  |  | Teleeducation + telemonitoring | 8 | -2 | -2  High Heterogeneity | -2  Differences in intervention (monitoring per week & SMBG schedule) | -2  Not available | -1 | 0 | + |
|  |  | Telecasemanagement + telemonitoring | 9 | -2 | -2  High Heterogeneity | -2  Differences in intervention (frequency of contacts & SMBG schedule) | -2  Not available | -1 | 1 | + |
|  |  | Teleeducation + Telecasemanagement | 9 | -2 | -2  High Heterogeneity | -2  Differences in intervention (components, educational contents, professional involved, frequency of contact, duration) | -2  Not available | -1 | 0 | + |
|  |  | Telecasemanagement + teleconsultation | 1 | - | - | - | - | - | - | Only 1 trial |
| [29] | overall | | 28 | -2  (LoAC, LoB, LTFU) | -2  Moderate Heterogeneity, CI overlaps | -2  Surrogate marker,  Differences in interventions, medium used and professional involved | -2  Large CIs | -1 | 0 | + |
|  | Population characteristics | adults | 15 | -2  (LoAC, LoB, LTFU) | -2  High Heterogeneity, CI overlaps | -2  Differences in interventions, medium used and professional involved | -2  Large CIs | -1 | 0 | + |
|  |  | Children and adolescents | 11 | -2  (LoAC, LoB, LTFU) | -1  CI overlaps | -2  Differences in interventions, medium used and professional involved | -2  Large CIs | -1 | 0 | + |
|  |  | Median baseline < 9.0% | 16 | -2  (LoAC, LoB, LTFU) | -2  Not available | -2  Not available | -2  Not available | -1 | 0 | + |
|  |  | Median baseline ≥ 9.0% | 12 | -2  (LoAC, LoB, LTFU) | -2  Not available | -2  Not available | -2  Not available | -1 | 0 | + |
|  | Follow-up | 3 months | 2 | -2  (LoAC, LoB, LTFU) | -2  High Heterogeneity, CI overlaps | -2  Differences in interventions, medium used and professional involved | -2  Large CIs | -1 | 0 | + |
|  |  | 6 months | 2 | -2  (LoAC, LoB, LTFU) | -2  High Heterogeneity, CI overlaps | -2  Differences in interventions, medium used and professional involved | -2  Large CIs | -1 | 0 | + |
|  | Intervention characteristics | Teleconsultation | 2 | -2  (LoAC, LoB, LTFU) | -1  CIs overlap | -1  Varying interventions (medium used and professionals involved) | -2  Large CIs | -1 | 0 | + |
|  |  | Telecase-management | 1 | - | - | - | - | - | - | Only 1 trial |
|  |  | Teleeducation | 4 | -2  (LoAC, LoB, LTFU) | -2  High Heterogeneity, CI overlaps | -1  Varying interventions (medium used and professionals involved) | -1  Large CIs | -1 | 0 | + |
|  |  | Teleeducation + teleconsultation | 1 |  |  |  |  |  |  | Only 1 trial |
|  |  | High intensity (direct contact at least once a week) | 13 | -2  (LoAC, LoB, LTFU) | -2  Not available | -2  Not available | -2  Not available | -1 | 0 | + |
|  |  | No high intensity | 14 | -2  (LoAC, LoB, LTFU) | -2  Not available | -2  Not available | -2  Not available | -1 | 0 | + |
|  |  | Intervention duration ≥ 6 months | 21 | -2  (LoAC, LoB, LTFU) | -2  Not available | -2  Not available | -2  Not available | -1 | 0 | + |
|  |  | Intervention duration < 6 months | 7 | -2  (LoAC, LoB, LTFU) | -2  Not available | -2  Not available | -2  Not available | -1 | 0 | + |
|  |  | Multi component (≥ 2 components) | 12 | -2  (LoAC, LoB, LTFU) | -2  Not available | -2  Not available | -2  Not available | -1 | 0 | + |
|  |  | No multi-component intervention (< 3 components) | 16 | -2  (LoAC, LoB, LTFU) | -2  Not available | -2  Not available | -2  Not available | -1 | 0 | + |
|  |  | Individualised assessment included | 13 | -2  (LoAC, LoB, LTFU) | -2  Not available | -2  Not available | -2  Not available | -1 | 0 | + |
|  |  | No individualised assessment | 15 | -2  (LoAC, LoB, LTFU) | -2  Not available | -2  Not available | -2  Not available | -1 | 0 | + |
|  |  | Audit & feedback | 24 | -2  (LoAC, LoB, LTFU) | -2  Not available | -2  Not available | -2  Not available | -1 | 0 | + |
|  |  | No audit & feedback | 4 | -2  (LoAC, LoB, LTFU) | -2  Not available | -2  Not available | -2  Not available | -1 | 0 | + |
| [30] | Overall (T1 + T2) | | 22 | -2  Not available) | -2  High Heterogeneity | -2  Varying populations, Varying interventions and durations, differences in settings (tertiary clinic and community health) | -2  Large Cis | -2  Not available | 1 | + |
|  | Overall T1 | | 9 | -2  Not available | -1  moderate heterogeneity | -2  Varying treatment durations, no baseline HbA1c reported | -1  Large Cis | -2  Not available | 0 | + |
|  | Overall T2 | | 10 | -2  Not available | -2  High Heterogeneity | -2  Varying interventions and durations, differences in settings (tertiary clinic and community health) | -1  Large Cis | -2  Not available | 1 | + |
|  | Unspecified diabetes type | | 3 | -2  Not available | 0 | -2  Varying interventions and durations, differences in settings (tertiary clinic and community health) | -1  Large Cis | -2  Not available | 0 | + |
|  | Population characteristics | age ≤ 25 years | 5 | -2  Not available | -2  Heterogeneity unclear | -2  Not available | -2  Not available | -2  Not available | 0 | + |
|  |  | age > 25 years | 17 | -2  Not available | -2  Heterogeneity unclear | -2  Not available | -2  Not available | -2  Not available | 0 | + |
|  |  | BMI ≥ 25 | 7 | -2  Not available | -2  Heterogeneity unclear | -2  Not available | -2  Not available | -2  Not available | 0 | + |
|  |  | 24 ≤ BMI < 25 | 3 | -2  Not available | -2  Heterogeneity unclear | -2  Not available | -2  Not available | -2  Not available | 0 | + |
|  |  | BMI unspecified | 12 | -2  Not available | -2  Heterogeneity unclear | -2  Not available | -2  Not available | -2  Not available | 0 | + |
|  | Intervention type | Web + mobile | 12 | -2  Not available | -2  Heterogeneity unclear | -2  Varying intervention durations, differences in settings (tertiary clinic and community health), difference in type of diabetes | -1  Large Cis | -2  Not available | 0 | + |
|  |  | mobile | 10 | -2  Not available | -2  Heterogeneity unclear | -2  Varying intervention durations, differences in settings (tertiary clinic and community health), difference in type of diabetes | -1  Large Cis | -2  Not available | 0 | + |
|  |  | intervention content (Both medication adjustment  and SMBG) | 13 | -2  Not available | -2  Heterogeneity unclear | -2  Varying intervention durations, differences in settings (tertiary clinic and community health), difference in type of diabetes | -2  Not available | -2  Not available | 0 | + |
|  | Intervention frequency | daily | 15 | -2  Not available | -2  Heterogeneity unclear | -2  Not available | -2  Not available | -2  Not available | 0 | + |
|  |  | Weekly | 3 | -2  Not available | -2  Heterogeneity unclear | -2  Not available | -2  Not available | -2  Not available | 0 | + |
|  |  | Not specified | 4 | -2  Not available | -2  Heterogeneity unclear | -2  Not available | -2  Not available | -2  Not available | 0 | + |
| [32] | Overall (T1 + T2) | | 13 | -1 | -2  High Heterogeneity | -2  Differences in populations (age, gender, baseline HbA1c, target group), intervention (device/medium used, professional involved, frequency of interaction with professional) | -1  Large Cis | -1 | 0 | + |
|  | Overall (T1) | | 2 | -2 | -2  Heterogeneity unclear | 0 | 0 | -1 | 1 | ++ |
|  | Overall (T2) | | 6 | -2 | -2  Heterogeneity unclear | -1  Differences in populations (gender) | -1  Large Cis | -1 | 0 | + |
|  | Population characteristics | baseline HbA1c < 8.0 % | 6 | -1 | -2  Heterogeneity unclear, Cis inconsistant | -2  Differences in intervention (device/medium used, professional involved, frequency of interaction with professional) | 0 | -1 | 0 | + |
|  |  | baseline HbA1c ≥ 8.0 % | 8 | -1 | -2  Heterogeneity unclear | -2  Differences in intervention (device/medium used, professional involved, frequency of interaction with professional) | -1  Large Cis | -1 | 1 | + |
|  | Intervention characteristics | prescription through TM | 4 | -2 | -2  Heterogeneity unclear | -2  unknown | -2  unknown | -1 | 1 | + |
|  |  | no prescription through TM | 7 | -2 | -2  Heterogeneity unclear | -2  unknown | -2  unknown | -1 | 0 | + |
|  | Intervention duration | 6 months | 6 | -1 | -2  Heterogeneity unclear | -2  Differences in populations (type of disease, age, gender, baseline HbA1c, target group), intervention (device/medium used, professional involved, frequency of interaction with professional) | 0 | -1 | 0 | + |
|  |  | 12 months | 7 | -1 | -2  Heterogeneity unclear | -2  Differences in populations (age, gender, baseline HbA1c, target group), intervention (device/medium used, professional involved, frequency of interaction with professional) | -1  Large Cis | -1 | 0 | + |
|  | Therapist involved | physician intervention | 3 | -2 | -2  Heterogeneity unclear | -2  Differences in populations (age, gender, type of disease, baseline HbA1c, target group), intervention (device/medium used, frequency of interaction with professional) | -1  Large Ci | -1 | 0 | + |
|  |  | nurse intervention | 9 | -1 | -2  Heterogeneity unclear | -2  Differences in populations (age, gender, type of disease, baseline HbA1c, target group), intervention (device/medium used, frequency of interaction with professional) | -1  Large Cis | -1 | 0 | + |
| [37] | Follow-up | < 6 months | 5 | -2  High risk of unblinding, unclear risk of detection bias, AC & selective reporting | -1  Cis overlap | -2  Difference in interventions (type, intensity, frequency, and BCT techniques used), varying settings) | -1 | -2  Not available | 0 | + |
|  |  | ≥ 6 months | 6 | -2  High risk of unblinding, unclear risk of detection bias, AC & selective reporting | -1  moderate heterogeneity | -2  Difference in interventions (type, intensity, frequency, and BCT techniques used), varying settings) | -1 | -2  Not available | 0 | + |
|  | Intervention characteristic | Computer-based | 11 | -2  High risk of unblinding, unclear risk of detection bias, AC & selective reporting | -2  moderate heterogeneity, CIs overlap | -2  Difference in interventions (type, duration, intensity, frequency, and BCT techniques used), varying follow-up durations, varying settings) | -1 | -2  Not available | 0 | + |
|  |  | Mobile phone based | 3 | -2  High risk of unblinding, unclear risk of detection bias, AC & selective reporting | 0 | -2  Difference in interventions (type, duration, intensity, frequency, and BCT techniques used), varying follow-up durations, varying settings) | -1 | -2  Not available | 1 | ++ |
|  |  | Home-based | 4 | -1  unclear risk of unblinding, unclear risk of detection bias, AC & selective reporting | 0 | -2  Difference in interventions (type, duration, intensity, frequency, and BCT techniques used), varying follow-up durations, varying settings) | -1 | -2  Not available | 0 | + |
| [39] | overall | | 12 | -2 | -1  Cis overlap | -2  Differences in interventions (experts involved), varying settings/ countries, inhomogeneous populations | -2  Large CIs | -2  Not available | 0 | + |
| [44] | overall | | 10 | -1  High risk of unblinding, unclear risk of detection bias & AC | -1  moderate heterogeneity | -2  Difference in interventions (SMS, monitoring), varying intervention durations, varying settings, varying populations) | -1 | -2 | 1 | ++ |
|  | Population characteristics | < 55 years | 5 | -1  High risk of unblinding, unclear risk of detection bias & AC | -1  SE moderate | -2  Difference in interventions (SMS, monitoring), varying intervention durations, varying settings) | -1 | -2 | 1 | ++ |
|  |  | ≥55 years | 5 | -1  High risk of unblinding, unclear risk of detection bias & AC | -1  SE moderate | -2  Difference in interventions (SMS, monitoring), varying intervention durations, varying settings) | -1 | -2 | 0 | + |
|  |  | Diagnosis < 7 years ago | 4 | -1  High risk of unblinding, unclear risk of detection bias & AC | -1  SE moderate | -2  Difference in interventions (SMS, monitoring), varying intervention durations, varying settings) | -1 | -2 | 1 | ++ |
|  |  | Diagnosis ≥ 7 years ago | 3 | 0 | -1  SE moderate | -2  Difference in interventions (SMS, monitoring), varying intervention durations, varying settings) | -1 | -2 | 0 | ++ |
|  |  | Baseline HbA1c < 8% | 5 | -1  High risk of unblinding, unclear risk of detection bias & AC | -1  SE moderate | -2  Difference in interventions (SMS, monitoring), varying intervention durations, varying settings) | -1 | -2 | 1 | ++ |
|  |  | Baseline HbA1c ≥ 8% | 5 | -2  High risk of unblinding, unclear risk of detection bias & AC | -1  SE moderate | -2  Difference in interventions (SMS, monitoring), varying intervention durations, varying settings) | -1 | -2 | 0 | + |
|  | Intervention characteristics | SMS (interactive) | 6 | -1  High risk of unblinding, unclear risk of detection bias & AC | -1  SE moderate | -2  varying intervention durations, varying settings, varying populations | -1 | -2 | 1 | ++ |
|  |  | SMS (unidirectional) | 4 | -2  High risk of unblinding, unclear risk of detection bias & AC | -1  SE moderate | -2  varying intervention durations, varying settings, varying populations | -1 | -2 | 0 | + |
|  |  | Communication tool: SMS only | 6 | -1  High risk of unblinding, unclear risk of detection bias & AC | -1  SE moderate | -2  varying intervention durations, varying settings, varying populations | -1 | -2 | 0 | ++ |
|  |  | Communication tool: both SMS + web | 4 | -1  High risk of unblinding, unclear risk of detection bias & AC | -2  SE high | -2  varying intervention durations, varying settings, varying populations | -1 | -2 | 1 | + |
|  | Intervention duration | < 6 months | 6 | -1  High risk of unblinding, unclear risk of detection bias & AC | -1  SE moderate | -2  Difference in interventions (SMS, monitoring), varying settings, varying populations | -1 | -2 | 1 | ++ |
|  |  | ≥ 6months | 4 | -2  High risk of unblinding, unclear risk of detection bias & AC | -1  SE moderate | -2  Difference in interventions (SMS, monitoring), varying settings, varying populations | -1 | -2 | 0 | + |
| [45] |  | overall | 35 | -2  High risk of unblinding, unclear risk of detection bias & AC | -1  moderate heterogeneity | -2  Difference in interventions (and devices used), varying countries, differences in populations (age range) | -1 | 0 | 0 | ++ |
|  | Intervention characteristics | ≤ 3 months | 10 | -2  High risk of unblinding, unclear risk of detection bias & AC | 0 | -2  Difference in interventions (and devices used), varying countries, differences in populations (age range) | -1 | 0 | 1 | ++ |
|  |  | 4 – 6 months | 10 | -2  High risk of unblinding, unclear risk of detection bias & AC | 0 | -2  Difference in interventions (and devices used), varying countries, differences in populations (age range) | -1 | 0 | 0 | + |
|  |  | > 6 months | 15 | -1  High risk of unblinding, unclear risk of detection bias & AC | -2  High heterogeneity | -2  Difference in interventions (and devices used), varying countries, differences in populations (age range) | 0 | 0 | 0 | ++ |
|  |  | Only web-based | 8 | -1  High risk of unblinding, unclear risk of detection bias & AC | -1  moderate heterogeneity | -2  Difference in interventions (and devices used), varying countries, differences in populations (age range) | 0 | 0 | 0 | ++ |
|  |  | Mobile-phone based | 8 | -2  High risk of unblinding, unclear risk of detection bias & AC | 0 | -2  Difference in interventions, varying countries, differences in populations (age range) | 0 | 0 | 0 | ++ |
|  |  | Mobile + web-based | 9 | -2  High risk of unblinding, unclear risk of detection bias & AC | -1  moderate heterogeneity | -2  Difference in interventions, varying countries, differences in populations (age range) | -1 | 0 | 1 | ++ |
|  |  | Web-based education | 14 | -2  High risk of unblinding, unclear risk of detection bias & AC | -1  moderate heterogeneity | -2  Difference in interventions, varying countries, differences in populations (age range) | -1 | 0 | 1 | ++ |
|  |  | No web-based education | 20 | -2  High risk of unblinding, unclear risk of detection bias & AC | -1  moderate heterogeneity | -2  Difference in interventions, varying countries, differences in populations (age range) | -1 | 0 | 0 | + |
|  |  | others | 10 | -2  High risk of unblinding, unclear risk of detection bias & AC | 0 | -2  Difference in interventions (and devices used), varying countries, differences in populations (age range) | 0 | 0 | 0 | ++ |
|  |  | Feedback (manual) | 22 | -2  High risk of unblinding, unclear risk of detection bias & AC | -1  moderate heterogeneity | -2  Difference in interventions (and devices used), varying countries, differences in populations (age range) | -1 | 0 | 1 | ++ |
|  |  | Feedback (Automated) | 5 | -2  High risk of unblinding, unclear risk of detection bias & AC | 0 | -2  Difference in interventions (and devices used), varying countries, differences in populations (age range) | 0 | 0 | 1 | ++ |
|  |  | Feedback (unclear) | 8 | -2  High risk of unblinding, unclear risk of detection bias & AC | 0 | -2  Difference in interventions (and devices used), varying countries, differences in populations (age range) | 0 | 0 | 0 | ++ |
| [46] | Follow-up | 3 months | 9 | -2  High risk of unblinding, unknown risk of AC, attrition bias | -1  CIs overlap | -1  Difference in interventions (staff involved), varying follow-up durations, varying settings) | -1  Small sample sizes, large CIs | -2  Not available | 0 | + |
|  |  | 6 months | 11 | -2  High risk of unblinding, unknown risk of AC, attrition bias | -2  High heterogeneity | -1  Difference in interventions (staff involved), varying follow-up durations, varying settings) | -1  Small sample sizes, large CIs | -2  Not available | 0 | + |
|  |  | > 7 months | 3 | -1  High risk of unblinding, unknown risk of AC, attrition bias | -2  High heterogeneity | -1  Difference in interventions (staff involved), varying follow-up durations, varying settings) | -2  Small sample sizes, large CIs | -2  Not available | 0 | + |
| [48] | Overall | | 55 | -2  Not available | -2  High heterogeneity | -2  Difference in interventions (low/high level), varying follow-up durations, varying settings) | 0 | -1 | 0 | + |
|  | (T1 + T2) | | 9 | -2  Not available | -1  CIs inconsitent | -2  Difference in interventions (low/high level), varying follow-up durations, varying settings) | 0 | -1 | 0 | + |
|  | Overall (T1) | | 15 | -2  Not available | -2  High heterogeneity | -1  Difference in interventions (low/high level), varying follow-up durations) | 0 | -1 | 0 | + |
|  | Overall (T2) | | 31 | -2  Not available | --1  I² not available | -2  Difference in interventions (low/high level), varying follow-up durations, varying settings) | 0 | -1 | 1 | ++ |
|  | Population characteristics | ≥ 40 years | 40 | -2  Not available | --1  I² not available | -2  Difference in interventions (low/high level), varying follow-up durations, varying settings) | -1 | -1 | 1 | + |
|  |  | < 40 years | 14 | -2  Not available | --1  I² not available | -1  Difference in interventions (low/high level), varying follow-up durations) | 0 | -1 | 0 | ++ |
|  | Intervention duration | ≤ 6months | 30 | -2  Not available | --1  I² not available | -2  Difference in interventions (low/high level), varying follow-up durations, varying settings) | -1 | -1 | 1 | + |
|  |  | > 6months | 25 | -2  Not available | --1  I² not available | -2  Difference in interventions (low/high level), varying follow-up durations, varying settings) | 0 | -1 | 0 | + |
|  | Intervention characteristic | Teleconsultation component | 18 | -2  Not available | --1  I² not available | -2  Difference in interventions (low/high level), varying follow-up durations, varying settings) | 0 | -1 | 1 | + |
| [49] | Intervention characteristics | Telephone-delivered intervention (phone calls) | 5 | -1  High risk of unblinding (prf.Bias), ITT | -2  High heterogeneity | -2  Difference in interventions (with and without personal contact, frequency of calls), varying intervention durations | -1 | 0 | 0 | + |
| [51] | Overall | | 42 | -1  High risk of unblinding (prf.Bias), ITT | -2  High heterogeneity | -2  Difference in populations (type of disease, average age), Difference in interventions, varying intervention durations, varying countries | 0 | -1  Visual PB but non-sign. | 0 | ++ |
|  | (T1 + T2) | | 9 | -1  High risk of unblinding (prf.Bias), ITT | -2  High heterogeneity | -2  Difference in populations (type of disease, average age), Difference in interventions, varying intervention durations, varying countries | -1 | -1  Visual PB but non-sign. | 0 | + |
|  | Overall (T1) | | 12 | -1  High risk of unblinding (prf.Bias), ITT | -2  High heterogeneity | -1 | 0 | -1  Visual PB but non-sign. | 0 | ++ |
|  | Overall (T2) | | 21 | -1  High risk of unblinding (prf.Bias),ITT | -2  High heterogeneity | -2  Difference in populations (average age), Difference in interventions and settings, varying intervention durations | -1 | -1  Visual PB but non-sign. | 0 | + |
|  | Follow-up | ≤ 6 months | 25 | -1  High risk of unblinding (prf.Bias),ITT | -2  High heterogeneity | -2  Difference in populations (type of disease, average age), Difference in interventions, varying intervention durations, varying countries | -1 | -1  Visual PB but non-sign. | 1 | ++ |
|  |  | > 6 months | 17 | -1  High risk of unblinding (prf.Bias),ITT | -2  High heterogeneity | -2  Difference in populations (type of disease, average age), Difference in interventions, varying intervention durations, varying countries | -1 | -1  Visual PB but non-sign. | 1 | ++ |
|  | Population characteristics | < 40 years | 11 | -1  High risk of unblinding (prf.Bias),ITT | -2  High heterogeneity | -2  Difference in populations (type of disease), Difference in interventions, varying intervention durations, varying countries | -1 | -1  Visual PB but non-sign. | 1 | ++ |
|  |  | 41 to 50 years | 8 | -1  High risk of unblinding (prf.Bias),ITT | -2  High heterogeneity | -2  Difference in populations (type of disease, average age), Difference in interventions, varying intervention durations, varying countries | -1 | -1  Visual PB but non-sign. | 1 | ++ |
|  |  | > 50 years | 17 | -1  High risk of unblinding (prf.Bias),ITT | -2  High heterogeneity | -2  Difference in populations (type of disease, average age), Difference in interventions, varying intervention durations, varying countries | 0 | -1  Visual PB but non-sign. | 2 | ++ |
| [53] | Overall (T1 + T2) | | 34 | -2  Unclear risk of SB, AC, Perf.B, Det.B., ITT, high risk of Rep. bias in 25% | -2  High heterogeneity, CIs overlap | -2  Difference in populations (type of disease, average age, diabetes duration), Difference in interventions, varying intervention durations, frequency of feedback | -2  Large CIs | -2 | 0 | + |
|  | Overall (T1) | | 7 | -2  Risk of SB, Perf.B, Detec.B, Rep.B. | 0 | -2  Difference in populations ( average age, diabetes duration), Difference in interventions, varying intervention durations, frequency of feedback | -2  Large CIs | -2 | 0 | + |
|  | Overall (T2) | | 21 | -2  Risk of SB, Perf.B, Detec.B, Rep.B. | -1  moderate heterogeneity | -2  Difference in populations (average age, diabetes duration), Difference in interventions, varying intervention durations, frequency of feedback | -1  Large CIs | -2 | 0 | + |
|  | (T1 + T2) | | 6 | -2  Risk of SB, Perf.B, Detec.B, Rep.B. | -1 | -2  Difference in populations (average age, diabetes duration), Difference in interventions, varying intervention durations, frequency of feedback | 0 | -2 | 0 | + |
|  | Intervention characteristics | Web-based only | 15 | -2  Risk of SB, Perf.B, Detec.B, Rep.B. | -2  High heterogeneity | -2  Difference in populations (type of disease, average age, diabetes duration), Difference in interventions, varying intervention durations, frequency of feedback | -2  Large CIs | -2 | 1 | + |
|  |  | Mobile only | 3 | -2  Risk of SB, Perf.B, Detec.B, Rep.B. | 0 | -2  Difference in populations (type of disease, average age, diabetes duration), Difference in interventions, varying intervention durations, frequency of feedback | 0 | -2 | 0 | ++ |
|  |  | Web + mobile | 16 | -2  Risk of SB, Perf.B, Detec.B, Rep.B. | -2  High heterogeneity | -2  Difference in populations (type of disease, average age, diabetes duration), Difference in interventions, varying intervention durations, frequency of feedback | -2  Large CIs | -2 | 1 | + |
|  | Intervention duration | ≤ 3 months | 13 | -2  Risk of SB, Perf.B, Detec.B, Rep.B. | 0 | -2  Difference in populations (type of disease, average age, diabetes duration), Difference in interventions, varying intervention durations, frequency of feedback | -2  Large CIs | -2 | 1 | + |
|  |  | 3 -12 months | 11 | -2  Risk of SB, Perf.B, Detec.B, Rep.B. | 0 | -2  Difference in populations (type of disease, average age, diabetes duration), Difference in interventions, varying intervention durations, frequency of feedback | -2  Large CIs | -2 | 0 | + |
|  |  | > 12 months | 10 | -1  Risk of SB, Perf.B, Detec.B, Rep.B. | -2  High heterogeneity | -2  Difference in populations (type of disease, average age, diabetes duration), Difference in interventions, varying intervention durations, frequency of feedback | -2  Large CIs | -2 | 0 | + |
| [59] | overall | | 16 | -1  Unclear/high RoBlinding | -1  CIs inconsitent | -1  Heterogeneous populations (T1 and T2DM), devices (intervention) differing, | -1 | -2  Not available | 0 | + |
|  | Population characteristics | Baseline HbA1c < 9.0% | n.s. | -1  Unclear to high RoBlinding | -2  Not available | -1  Varying populations (gender), varying devices | -1 | -2  Not available | 0 | + |
|  |  | Baseline HbA1c ≥ 9.0% | n.s. | -1  Unclear RoBlinding | -2  Not available | -1  Varying populations (gender), varying devices | 0 | -2  Not available | 0 | + |
| [58] | overall | | 17 | -1  Unclear or high risk of blinding, incomplete outcome data | -1 | -2  Difference in populations unknown (baseline HbA1c), Difference in interventions (components used), varying intervention durations, frequency of feedback, professionals involved | -1 | -1 | 1 | + |
|  | Follow-up | ≤ 6 months | 11 | -2  Risk of SB, Perf.B, Detec.B, Rep.B. | -2  Moderate heterogeneiety, Cis inconsistant | -1  Difference in populations unknown (baseline HbA1c), Difference in interventions (components used), varying intervention durations, frequency of feedback, professionals involved | 0 | -1 | 0 | + |
|  |  | > 6 months | 6 | -2  Risk of SB, Perf.B, Detec.B, Rep.B. | -1  Cis inconsistant | -2  Difference in populations unknown (baseline HbA1c), Difference in interventions (components used), varying intervention durations, frequency of feedback, professionals involved | -1 | -1 | 0 | + |
|  | Population characteristics | Diagnosis < 8.5 years ago | 7 | -2  Risk of SB, Perf.B, Detec.B, Rep.B. | -1  CIs inconsitent | -2  Difference in populations, Difference in interventions (components used), varying intervention durations, frequency of feedback, professionals involved | 0 | -1 | 1 | + |
|  |  | Diagnosis ≥8.5 years ago | 4 | -1  Risk of SB, Perf.B, Detec.B, Rep.B. | -1  CIs inconsitent | -2  Difference in populations, Difference in interventions (components used), varying intervention durations, frequency of feedback, professionals involved | -1 | -1 | 0 | + |
|  |  | Diagnosis time undetermined | 6 | -2  Risk of SB, Perf.B, Detec.B, Rep.B. | -1  Moderate heterogeneity | -2  Difference in populations, Difference in interventions (components used), varying intervention durations, frequency of feedback, professionals involved | 0 | -1 | 0 | + |
|  |  | patient age <55 years | 7 | -1  Risk of SB, Perf.B, Detec.B, Rep.B. | -2  High heterogeneity | -2  Difference in populations, Difference in interventions (components used), varying intervention durations, frequency of feedback, professionals involved | -1 | -1 | 0 | + |
|  |  | patient age ≥55 years | 8 | -1  Risk of SB, Perf.B, Detec.B, Rep.B. | 0 | -2  Difference in populations, Difference in interventions (components used), varying intervention durations, frequency of feedback, professionals involved | 0 | -1 | 0 | ++ |
|  |  | patient age undetermined | 2 | -2  Risk of SB, Perf.B, Detec.B, Rep.B. | -1  CIs inconsitent | -2  Not available | -2  Not available | -1 | 0 | + |
|  |  | baseline HbA1c ≤ 8.0 % | 6 | -1  Risk of SB, Perf.B, Detec.B, Rep.B. | 0 | -2  Difference in populations, Difference in interventions (components used), varying intervention durations, frequency of feedback, professionals involved | 0 | -1 | 0 | ++ |
|  |  | baseline HbA1c> 8.0 % | 11 | -2  Risk of SB, Perf.B, Detec.B, Rep.B. | -1  Moderate heterogeneity | -2  Difference in populations, Difference in interventions (components used), varying intervention durations, frequency of feedback, professionals involved | -2 | -1 | 0 | + |
|  |  | baseline BMI < 30 | 5 | -1  Risk of SB, Perf.B, Detec.B, Rep.B. | -1  CIs inconsitent | 0 | 0 | -1 | 0 | +++ |
|  |  | baseline BMI ≥ 30 | 10 | -1  Risk of SB, Perf.B, Detec.B, Rep.B. | -1  CIs inconsitent | -2  Difference in populations, Difference in interventions (components used), varying intervention durations, frequency of feedback, professionals involved | -2 | -1 | 0 | + |
|  |  | baseline BMI undetermined | 2 | -1  Risk of SB, Perf.B, Detec.B, Rep.B. | -2  High heterogeneity | -1  Difference in populations unknown (baseline HbA1c), Difference in interventions (components used), varying intervention durations, frequency of feedback, professionals involved | -1 | -1 | 0 | + |
|  | Intervention characteristics | High smartphone technology exposure | 7 | -1  Risk of SB, Perf.B, Detec.B, Rep.B. | -1  CIs inconsitent | -1  Difference in populations unknown (baseline HbA1c), Difference in interventions (components used), varying intervention durations, frequency of feedback, professionals involved | -1 | -1 | 0 | + |
|  |  | Low smartphone technology exposure | 4 | -1  Risk of SB, Perf.B, Detec.B, Rep.B. | -1  CIs inconsitent | -2  Difference in populations unknown (baseline HbA1c), Difference in interventions (components used), varying intervention durations, frequency of feedback, professionals involved | -1 | -1 | 0 | + |
|  |  | unclear smartphone technology exposure | 6 | -1  Risk of SB, Perf.B, Detec.B, Rep.B. | -1  Moderate heterogeneity, CIs inconsitent | -2  Difference in populations unknown (baseline HbA1c), Difference in interventions (components used), varying intervention durations, frequency of feedback, professionals involved | -2 | -1 | 0 | + |
|  |  | HCP contact time equal to control | 8 | -1  Risk of SB, Perf.B, Detec.B, Rep.B. | -1  CIs inconsitent | -2  Difference in populations unknown (baseline HbA1c), Difference in interventions (components used), varying intervention durations, frequency of feedback, professionals involved | -1 | -1 | 0 | + |
|  |  | HCP contact time more often than in control | 1 | - | - | - | - | - | - | Only 1 trial |
|  |  | HCP contact undetermined | 8 | -2  Risk of SB, Perf.B, Detec.B, Rep.B. | -1  Moderate heterogeneity | -2  Difference in populations unknown (baseline HbA1c), Difference in interventions (components used), varying intervention durations, frequency of feedback, professionals involved | -1 | -1 | 0 | + |
| [57] | Overall (T1 + T2) | | 12 | -2  (LoAC, high RoB, high risk of rep. bias) | -2  High heterogeneity | -2  Difference in populations, Difference in interventions, varying intervention durations, frequency of feedback, professionals involved | -2  Large CIs | -1 | 0 | + |
|  | Overall (T1) | | 5 | -2  (attrition bias, high RoBlinding, high risk of rep. bias) | -2  High heterogeneity, CIs inconsistent | -1  Difference in interventions, varying intervention durations, frequency of feedback, professionals involved | -1  Large CIs | -1 | 0 | + |
|  | Overall (T2) | | 5 | -2  (attrition bias, high RoBlinding, high risk of rep. bias) | -1  moderate heterogeneity | -2  Difference in interventions, varying intervention durations, frequency of feedback, professionals involved | -2  Large CIs | -1 | 1 | + |
|  | Intervention characteristics | Complication prevention module | 2 | -2  (attrition bias, high RoBlinding, high risk of rep. bias) | 0 | -1 | -1  Large CIs | -1 | 1 | ++ |
|  |  | No complication prevention module | 10 | -2  (attrition bias, high RoBlinding, high risk of rep. bias) | -2  High heterogeneity | -1 | -1 | -1 | 0 | + |
|  |  | High risk interventions | 3 | -2  (attrition bias, high RoBlinding, high risk of rep. bias) | -2  High heterogeneity | -1 | -2 | -1 | 0 | + |
|  |  | Potential risk interventions | 9 | -2  (attrition bias, high RoBlinding, high risk of rep. bias) | -1  moderate heterogeneity | -1 | -1 | -1 | 0 | + |
|  |  | Personalised feedback | 8 | -2  (attrition bias, high RoBlinding, high risk of rep. bias) | -2  High heterogeneity | -1 | -1 | -1 | 0 | + |
|  |  | No personalised feedback | 4 | -2  (attrition bias, high RoBlinding, high risk of rep. bias) | -2  High heterogeneity | -1 | -2  Large CIs | -1 | 0 | + |
|  |  | manual data entry | 5 | -2  (attrition bias, high RoBlinding, high risk of rep. bias) | -2  high heterogeneity | -1 | -1 | -1 | 0 | + |
|  |  | Education wireless data transport from device | 6 | -2  (attrition bias, high RoBlinding, high risk of rep. bias) | -1  moderate heterogeneity | -1 | -1 | -1 | 0 | + |
|  |  | Structured display | 8 | -2  (attrition bias, high RoBlinding, high risk of rep. bias) | -1  Moderate heterogeneity | -1 | -2 | -1 | 1 | + |
|  |  | No structured display | 4 | -2  (attrition bias, high RoBlinding, high risk of rep. bias) | -2  High heterogeneity | -1 | 0 | -1 | 0 | + |
|  |  | Medication management | 8 | -2  (attrition bias, high RoBlinding, high risk of rep. bias) | -2  High heterogeneity | -1 | -1 | -1 | 0 | + |
|  |  | No medication management | 4 | -2  (attrition bias, high RoBlinding, high risk of rep. bias) | 0 | -1 | -1 | -1 | 0 | + |
|  |  | Lifestyle modification management | 11 | -2  (LoAC, high RoB, high risk of rep. bias) | -2  High heterogeneity | -1 | -2 | -1 | 0 | + |
|  |  | No lifestyle modification management | 1 | - | - | - | - | - | - | Only 1 trial |
|  |  | General education | 6 | -2  (attrition bias, high RoBlinding, high risk of rep. bias) | 0 | -1 | -1 | -1 | 0 | + |
|  |  | No *General education* | 6 | -2  (attrition bias, high RoBlinding, high risk of rep. bias) | -2  High heterogeneity | -1 | -1 | -1 | 0 | + |
| Yoshida et al., 2018 | overall | | 33 | -1  unclear allocate. Concealment, 80% RoB high/unclear | -2  High heterogeneity | -2 | -1  3 Ausreißer, aber alle favourn treatment | -1 | 1 | + |
|  | Intervention characteristics | HIT + Standard Care | 17 | -2  Not available | -2 | -1  varying intervention durations | 0 | -1 | 1 | + |
|  |  | Teleeducation component (mobile device) | 21 | -2  Not available | -2 | -2  11 included hybrid intervention, varying durations spread of countries, diff. in intervention durations | 0 | -1 | 1 | + |
|  |  | Teleeducation component (SMS / Texting) | 7 | -2  Not available | -2 | -1  spread of countries, varying basal HbA1c in IG and CG, diff. in intervention durations | -1  Large CI | -1 | 1 | + |
|  |  | Teleeducation component (web-based.) | 6 | -2  Not available | -1 | -1  diff. in intervention durations | 0 | -1 | 0 | ++ |
|  |  | Teleeducation component (other ICT) | 8 | -2  Not available | 0 | -2  Difference in interventions, varying intervention durations | -2 | -1 | 0 | + |
| Zhai, Zhu, Cai, Sun, & Zhao, 2014 | Overall (T2) | | 35 | -1  (LoAC, Blinding unclear or not present, LTFU) | -2 | -2  Potential differences in interventions (e.g. tailoring) and populations, time differences in outcomes | -2 | -2 | 0 | + |
|  | Intervention characteristics | Feedback (telephone) | 12 | -1  (LoAC, LoB, LTFU) | -2 | -1  Potential differences in interventions (e.g. tailoring) and populations, time differences in outcomes | -1 | -2 | 1 | + |
|  |  | Feedback (web-based) | 19 | -1  (LoAC, LoB, LTFU) | -2 | -2  Potential differences in interventions (e.g. tailoring) and populations, time differences in outcomes | -2 | -2 | 1 | + |
|  |  | Feedback (internet-transmitted) | 4 | 0 | 0 | -1  Potential differences in interventions (e.g. tailoring) and populations, time differences in outcomes | -1 | -2 | 0 | ++ |

Suppl. Table 2 Assessment of subgroup-specific certainty of outcomes (SBP and DBP) using GRADE

| **Author, Year** | **Subgroups** | | | | **Number of included Studies** | **Certainty Assessment** | | | | | **Clinical relevance** | **Certainty** |
| --- | --- | --- | --- | --- | --- | --- | --- | --- | --- | --- | --- | --- |
|  |  |  |  |  |  | **Risk of Bias** | **Inconsistency** | **Indirectness** | **Imprecision** | **Publication bias** |  |  |
| **Combination of target diseases** | | | | | | | | | | | | |
| Kelly et al., 2016 | Overall | | | | 12 (SBP) | -2 | -2  High heterogeneity, Cis inconsistant | -2 | -2 | -2 | 0 | + |
|  |  |  |  |  | 10 (DBP) | -2 | -2  High heterogeneity, Cis inconsistant | -2 | -2  Not available | -2 | 0 | + |
|  | Diabetes patients | | | | 2 (SBP) | -2 | -2  Moderate heterogeneity, Cis inconsistant | -2 | -1 | -2 | 0 | + |
| **Patients with hypertension** | | | | | | | | | | | | |
| Liu et al., 2013 | internet-based lifestyle interventions (SBP) | | | | 13 | -2  Not available | -2  Moderate heterogeneity, Cis inconsistant | -2  Differences in intervention (components and mode of delivery), intervention duration, times of data collection, population characteristics | -1 | -1 | 0 | + |
|  | internet-based lifestyle interventions (DBP) | | | | 13 | -2  Not available | -2  Moderate heterogeneity, Cis inconsistant | -2  Differences in intervention (components and mode of delivery), intervention duration, times of data collection, population characteristics | -1 | -2 | 0 | + |
|  | intervention duration | | <6 months (SBP) | | 8 | -2  Not available | -2 | -2  Differences in intervention (components and mode of delivery), intervention duration, times of data collection, population characteristics | -2 | -1 | 0 | + |
|  |  |  | 6-12 months (SBP) | | 5 | -2  Not available | -2 | -2  Differences in intervention (components and mode of delivery), intervention duration, times of data collection, population characteristics | -1 | -1 | 0 | + |
|  | proactive method of delivery (DBP) | | | | 10 | -2  Not available | -2 | -2 | -2 | -2 | 0 | + |
|  | reactive method of delivery (DBP) | | | | 3 | -2  Not available | -2 | -1  Difference in populations and intervention (diet and exercise) | -1 | -2 | 0 | + |
| Omboni et al., 2011 | Home Blood Pressure Telemonitoring | office measurement (SBP) | | | 11 | -2 | -1  Moderate heterogeneity, Cis inconsistant | -2  Differences in intervention, intervention duration, times of data collection, population characteristics unknown | -1 | -1 | 0 | + |
|  |  | office measurement (DBP) | | | 11 | -2 | -1  Moderate heterogeneity, Cis inconsistant | -2  Differences in intervention, intervention duration, times of data collection, population characteristics unknown | -1 | -1 | 0 | + |
|  |  | ambulatory measurement (SBP) | | | 3 | -2  Not available | -2  Not available | -2  Not available | -2  Not available | -2  Not available | 0 | + |
|  |  | ambulatory measurement (DBP) | | | 3 | -2  Not available | -2  Not available | -2  Not available | -2  Not available | -2  Not available | 0 | + |
| Omboni et al., 2013 | Home Blood Pressure Telemonitoring | Office measurement (SBP) | | | 17 | -2  Not available | -2  Moderate heterogeneity, Cis inconsistant | -2  Differences in populations, interventions and settings | -1 | -1 | 0 | + |
|  |  | Office measurement (DBP) | | | 15 | -2  Not available | -1  CIs inconsistant | 2  Differences in populations, interventions and settings | -1 | -1 | 0 | + |
|  |  | Ambulatory measurement (SBP) | | | 5 | -2  Not available | -1  CIs inconsistant | -2  Differences in populations, interventions and settings | -1 | -1 | 0 | + |
|  |  | Ambulatory measurement (DBP) | | | 5 | -2  Not available | -1  CIs inconsistant | -2  Differences in populations, interventions and settings | -1 | -1 | 0 | + |
| **Patients with diabetes** | | | | | | | | | | | | |
| Bonoto et al., 2017 | Overall (SBP) | | | | 4 | -2 | -2  Not available | -2  Differences in populations, interventions and settings | -2  Not available | -2  Not available | 0 | + |
|  | Overall (DBP) | | | | 4 | -2 | -2  Not available | -2  Differences in populations, interventions and settings | -2  Not available | -2  Not available | 0 | + |
| Cui et al., 2016 | Diabetes-selfmanagement (mHealth) (T2D) (SBP) | | | | 4 | -2 | -1  Cis inconsistant | -2  Differences in populations, interventions and settings | -1  Large CIs | -2  Not available | 0 | + |
|  | Diabetes-selfmanagement (mHealth) (T2D) (DBP) | | | | 4 | -2 | -1  Cis inconsistant | -2  Differences in populations, interventions and settings | -1  Large CIs | -2  Not available | 0 | + |
| Marcolino et al., 2013 | overall | | SBP | | 8 | -1 | -2  High heterogeneity, CIs inconsistent | -2  Differences in populations (diabetes duration, mean age), interventions (web, videoconferencing, change of medication) | -1  Large CIs | -1 | 0 | + |
|  |  |  | DBP | | 7 | -1 | -2  High heterogeneity, CIs inconsistent | -2  Differences in populations (diabetes duration, mean age), interventions (web, videoconferencing, change of medication) | -1  Large CIs | -1 | 0 | + |
| Toma et al., 2014 | SNS-based interventions (overall) (SBP) | | | | 5 | -2 | -1  CIs not available | -2  Not available | -2  Not available | -2 | 0 | + |
|  | SNS-based interventions (overall) (DBP) | | | | 5 | -2 | -1  CIs not available | -2  Not available | -2  Not available | -2 | 0 | + |
| Lee et al., 2017b | Overall (T1) (SBP) | | | | 2 | -2 | -1  Moderate heterogeneity, CI inconsistent | 0 | 0 | -1 | 0 | + |
|  | Overall (T1) (DBP) | | | | 2 | -2 | -1  Moderate heterogeneity, CI inconsistent | 0 | 0 | -1 | 0 | + |
| Lee et al., 2017a | Intervention characteristics | | Tele-educat. | SBP | 7 | -2  Not available | -2  Not available | -2  Not available | -2  Not available | -1 | 0 | + |
|  |  |  |  | DBP | 6 | -2  Not available | -1  Moderate heterogeneity, CI inconsistent | -2  Not available | -2  Not available | -1 | 0 | + |
|  |  |  | Tele- CM | SBP | 3 | -2  Not available | -2  Not available | -2  Not available | -2  Not available | -1 | 0 | + |
|  |  |  |  | DBP | 3 | -2  Not available | -2  Not available | -2  Not available | -2  Not available | -1 | 0 | + |
|  |  |  | Tele-Consult. | SBP | 3 | -2  Not available | -2  High heterogeneity, CIs inconsistent | -2  Not available | -2  Not available | -1 | 0 | + |
|  |  |  |  | DBP | 2 | -2  Not available | -2  High heterogeneity, CIs inconsistent | -2  Not available | -2  Not available | -1 | 0 | + |
|  |  |  | Tele-Mentoring | SBP | 5 | -2  Not available | -2  High heterogeneity, CIs inconsistent | -2  Not available | -2  Not available | -1 | 0 | + |
|  |  |  |  | DBP | 5 | -2  Not available | -2  High heterogeneity, CIs inconsistent | -2  Not available | -2  Not available | -1 | 0 | + |
|  |  |  | Tele-Educat. + Tele-Mon. | SBP | 4 | -2  Not available | -2  High heterogeneity, CIs inconsistent | -2  Not available | -2  Not available | -1 | 0 | + |
|  |  |  |  | DBP | 3 | -2  Not available | -2  High heterogeneity, CIs inconsistent | -2  Not available | -2  Not available | -1 | 0 | + |
|  |  |  | Tele-CM + Tele-Mon. | SBP | 5 | -2  Not available | -2  Not available | -2  Not available | -2  Not available | -1 | 0 | + |
|  |  |  |  | DBP | 5 | -2  Not available | -2  Not available | -2  Not available | -2  Not available | -1 | 0 | + |
|  |  |  | Tele-Educat.+ Tele-CM | SBP | 2 | -2  Not available | -2  High heterogeneity, CIs inconsistent | -2  Not available | -2  Not available | -1 | 0 | + |
|  |  |  |  | DBP | 1 | - | - | - | - | - | 0 | Only 1 trial |
| Wu et al., 2018a | Overall (SBP) | | | | 8 | -1  High risk of unblinding | -1  Moderate heterogeneity, CI inconsistent | -1  Differences in interventions and follow-up duration | -1 | -2  Not available | 0 | + |
|  | Overall (DBP) | | | | 8 | -1  High risk of unblinding | -2  High heterogeneity, CIs inconsistent | -1  Differences in interventions and follow-up duration | -1 | -2  Not available | 0 | + |
