# Supplementary material for: Mapping the Evidence on the Effectiveness of Telemedicine Interventions in Diabetes, Dyslipidemia, and Hypertension: An Umbrella Review of Systematic Reviews and Meta-Analyses
Source: J Med Internet Res. 2020 Mar 18;22(3):e16791. doi: 10.2196/16791 (PMC7113804; doi:10.2196/16791)
Supplement: Multimedia Appendix 9 [file jmir_v22i3e16791_app9.doc]

# Multimedia Appendix 10 – References of multimedia appendices

1. Alessa T, Abdi S, Hawley MS, de Witte L. Mobile Apps to Support the Self-Management of Hypertension: Systematic Review of Effectiveness, Usability, and User Satisfaction. JMIR mHealth and uHealth. 2018;6(7):e10723. PMID: 30037787. doi: 10.2196/10723.

2. Alharbi NS, Alsubki N, Jones S, Khunti K, Munro N, de Lusignan S. Impact of Information Technology-Based Interventions for Type 2 Diabetes Mellitus on Glycemic Control: A Systematic Review and Meta-Analysis. Journal of medical Internet research. 2016;18(11):e310. PMID: 27888169. doi: 10.2196/jmir.5778.

3. Angeles RN, Howard MI, Dolovich L. The effectiveness of web-based tools for improving blood glucose control in patients with diabetes Mellitus: A meta-analysis. Canadian Journal of Diabetes. 2011;35(4):344-52. PMID: 12011006780. doi: 10.1016/S1499-2671(11)54011-0.

4. Aspry KE, Furman R, Karalis DG, Jacobson TA, Zhang AM, Liptak GS, et al. Effect of health information technology interventions on lipid management in clinical practice: a systematic review of randomized controlled trials. Journal of clinical lipidology. 2013;7(6):546-60. PMID: 24314354. doi: 10.1016/j.jacl.2013.10.004.

5. Baron J, McBain H, Newman S. The impact of mobile monitoring technologies on glycosylated hemoglobin in diabetes: a systematic review. Journal of Diabetes Science and Technology. 2012;6(5):1185-96. PMID: 23063046. doi: 10.1177/193229681200600524.

6. Bonoto BC, de Araujo VE, Godoi IP, de Lemos LL, Godman B, Bennie M, et al. Efficacy of Mobile Apps to Support the Care of Patients With Diabetes Mellitus: A Systematic Review and Meta-Analysis of Randomized Controlled Trials. JMIR mHealth and uHealth. 2017;5(3):e4. PMID: 28249834. doi: 10.2196/mhealth.6309.

7. Cassimatis M, Kavanagh DJ. Effects of type 2 diabetes behavioural telehealth interventions on glycaemic control and adherence: a systematic review. J Telemed Telecare. 2012 Dec;18(8):447-50. PMID: 23209266. doi: 10.1258/jtt.2012.GTH105.

8. Connelly J, Kirk A, Masthoff J, MacRury S. The use of technology to promote physical activity in Type 2 diabetes management: a systematic review. Diabet Med. 2013;30(12):1420-32. PMID: 23870009. doi: 10.1111/dme.12289.

9. Cotter AP, Durant N, Agne AA, Cherrington AL. Internet interventions to support lifestyle modification for diabetes management: a systematic review of the evidence. J Diabetes Complications. 2014 Mar-Apr;28(2):243-51. PMID: 24332469. doi: 10.1016/j.jdiacomp.2013.07.003.

10. Cui M, Wu X, Mao J, Wang X, Nie M. T2DM Self-Management via Smartphone Applications: A Systematic Review and Meta-Analysis. PLoS One. 2016;11(11):e0166718. PMID: 27861583. doi: 10.1371/journal.pone.0166718.

11. David SK, Rafiullah MRM. Innovative health informatics as an effective modern strategy in diabetes management: A critical review. Int J Clin Pract. 2016;70(6):434-49. PMID: 610608283. doi: 10.1111/ijcp.12816.

12. de Jongh T, Gurol-Urganci I, Vodopivec-Jamsek V, Car J, Atun R. Mobile phone messaging for facilitating self-management of long-term illnesses. Cochrane Database Syst Rev. 2012;12:Cd007459. PMID: 23235644. doi: 10.1002/14651858.CD007459.pub2.

13. El-Gayar O, Timsina P, Nawar N, Eid W. A systematic review of IT for diabetes self-management: are we there yet? Int J Med Inform. 2013;82(8):637-52. PMID: 23792137. doi: 10.1016/j.ijmedinf.2013.05.006.

14. Farmer AJ, McSharry J, Rowbotham S, McGowan L, Ricci-Cabello I, French DP. Effects of interventions promoting monitoring of medication use and brief messaging on medication adherence for people with Type 2 diabetes: a systematic review of randomized trials. Diabet Med. 2016;33(5):565-79. PMID: 26470750. doi: 10.1111/dme.12987.

15. Faruque LI, Wiebe N, Ehteshami-Afshar A, Liu Y, Dianati-Maleki N, Hemmelgarn BR, et al. Effect of telemedicine on glycated hemoglobin in diabetes: a systematic review and meta-analysis of randomized trials. CMAJ: Canadian Medical Association journal = journal de l'Association medicale canadienne. 2017;189(9):E341-E64. PMID: 27799615. doi: 10.1503/cmaj.150885.

16. Fu H, McMahon SK, Gross CR, Adam TJ, Wyman JF. Usability and clinical efficacy of diabetes mobile applications for adults with type 2 diabetes: A systematic review. Diabetes Res Clin Pract. 2017;131:70-81. PMID: 28692830. doi: 10.1016/j.diabres.2017.06.016.

17. Garabedian LF, Ross-Degnan D, Wharam JF. Mobile Phone and Smartphone Technologies for Diabetes Care and Self-Management. Current Diabetes Reports. 2015;15(12):1-9. PMID: 26458380. doi: 10.1007/s11892-015-0680-8.

18. Garcia-Lizana F, Sarria-Santamera A. New technologies for chronic disease management and control: a systematic review. J Telemed Telecare. 2007;13(2):62-8. PMID: 17359568. doi: 10.1258/135763307780096140.

19. Greenwood DA, Young HM, Quinn CC. Telehealth Remote Monitoring Systematic Review: Structured Self-monitoring of Blood Glucose and Impact on A1C. J Diabetes Sci Technol. 2014;8(2):378-89. PMID: 24876591. doi: 10.1177/1932296813519311.

20. Hamine S, Gerth-Guyette E, Faulx D, Green BB, Ginsburg AS. Impact of mHealth chronic disease management on treatment adherence and patient outcomes: a systematic review. Journal of medical Internet research. 2015;17(2):e52-e. PMID: 25803266. doi: 10.2196/jmir.3951.

21. Holmen H, Wahl AK, Cvancarova Smastuen M, Ribu L. Tailored Communication Within Mobile Apps for Diabetes Self-Management: A Systematic Review. Journal of medical Internet research. 2017;19(6):e227. PMID: 28645890. doi: 10.2196/jmir.7045.

22. Holtz B, Lauckner C. Diabetes Management via Mobile Phones: A Systematic Review. Telemedicine and e-Health. 2012;18(3):175-84. PMID: 22356525. doi: 10.1089/tmj.2011.0119.

23. Hou C, Xu Q, Diao S, Hewitt J, Li J, Carter B. Mobile phone applications and self-management of diabetes: A systematic review with meta-analysis, meta-regression of 21 randomized trials and GRADE. Diabetes, obesity & metabolism. 2018;20(8):2009-13. PMID: 29582538. doi: 10.1111/dom.13307.

24. Huang Z, Tao H, Meng Q, Jing L. Effects of telecare intervention on glycemic control in type 2 diabetes: A systematic review and meta-analysis of randomized controlled trials. European Journal of Endocrinology. 2015;172(3):R93-R101. PMID: 25227131. doi: 10.1530/EJE-14-0441.

25. Kebede MM, Zeeb H, Peters M, Heise TL, Pischke CR. Effectiveness of Digital Interventions for Improving Glycemic Control in Persons with Poorly Controlled Type 2 Diabetes: A Systematic Review, Meta-analysis, and Meta-regression Analysis. Diabetes Technol Ther. 2018;20(11):767-82. PMID: 30257102. doi: 10.1089/dia.2018.0216.

26. Kelly JT, Reidlinger DP, Hoffmann TC, Campbell KL. Telehealth methods to deliver dietary interventions in adults with chronic disease: a systematic review and meta-analysis. The American journal of clinical nutrition. 2016;104(6):1693-702. PMID: 27935523. doi: 10.3945/ajcn.116.136333.

27. Krishna S, Boren SA, Balas EA. Healthcare via Cell Phones: A Systematic Review. Telemedicine and e-Health. 2009;15(3):231-40. PMID: 19382860. doi: 10.1089/tmj.2008.0099.

28. Lee SWH, Chan CKY, Chua SS, Chaiyakunapruk N. Comparative effectiveness of telemedicine strategies on type 2 diabetes management: A systematic review and network meta-analysis. Scientific reports. 2017;7(1):12680. PMID: 28978949. doi: 10.1038/s41598-017-12987-z.

29. Lee SWH, Ooi L, Lai YK. Telemedicine for the Management of Glycemic Control and Clinical Outcomes of Type 1 Diabetes Mellitus: A Systematic Review and Meta-Analysis of Randomized Controlled Studies. Frontiers in pharmacology. 2017;8:330. PMID: 28611672. doi: 10.3389/fphar.2017.00330.

30. Liang X, Wang Q, Yang X, Cao J, Chen J, Mo X, et al. Effect of mobile phone intervention for diabetes on glycaemic control: a meta-analysis. Diabet Med. 2011;28(4):455-63. PMID: 21392066. doi: 10.1111/j.1464-5491.2010.03180.x.

31. Liu S, Dunford SD, Leung YW, Brooks D, Thomas SG, Eysenbach G, et al. Reducing blood pressure with Internet-based interventions: a meta-analysis. The Canadian Journal of Cardiology. 2013;29(5):613-21. PMID: 23618507. doi: 10.1016/j.cjca.2013.02.007.

32. Marcolino MS, Maia JX, Alkmim MBM, Boersma E, Ribeiro AL. Telemedicine application in the care of diabetes patients: systematic review and meta-analysis. PloS One. 2013;8(11):e79246. PMID: 24250826. doi: 10.1371/journal.pone.0079246.

33. Mushcab H, Kernohan WG, Wallace J, Martin S. Web-Based Remote Monitoring Systems for Self-Managing Type 2 Diabetes: A Systematic Review. Diabetes Technol Ther. 2015;17(7):498-509. PMID: 25830528. doi: 10.1089/dia.2014.0296.

34. Omboni S, Guarda A. Impact of home blood pressure telemonitoring and blood pressure control: a meta-analysis of randomized controlled studies. Am J Hypertens. 2011;24(9):989-98. PMID: 21654858. doi: 10.1038/ajh.2011.100.

35. Omboni S, Gazzola T, Carabelli G, Parati G. Clinical usefulness and cost effectiveness of home blood pressure telemonitoring: meta-analysis of randomized controlled studies. Journal of Hypertension. 2013;31(3):455-67; discussion 67-68. PMID: 23299557. doi: 10.1097/HJH.0b013e32835ca8dd.

36. Or CK, Tao D. Does the use of consumer health information technology improve outcomes in the patient self-management of diabetes? A meta-analysis and narrative review of randomized controlled trials. Int J Med Inform. 2014 May;83(5):320-9. PMID: 24534118. doi: 10.1016/j.ijmedinf.2014.01.009.

37. Pal K, Eastwood SV, Michie S, Farmer A, Barnard ML, Peacock R, et al. Computer-based interventions to improve self-management in adults with type 2 diabetes: a systematic review and meta-analysis. Diabetes Care. 2014;37(6):1759-66. PMID: 24855158. doi: 10.2337/dc13-1386.

38. Paré G, Moqadem K, Pineau G, St-Hilaire C. Clinical effects of home telemonitoring in the context of diabetes, asthma, heart failure and hypertension: a systematic review. Journal of medical Internet research. 2010;12(2):e21. PMID: 20554500 doi: 10.2196/jmir.1357.

39. Polisena J, Tran K, Cimon K, Hutton B, McGill S, Palmer K. Home telehealth for diabetes management: A systematic review and meta-analysis. Diabetes, Obesity and Metabolism. 2009;11(10):913-30. PMID: 355391218. doi: 10.1111/j.1463-1326.2009.01057.x.

40. Porter J, Huggins CE, Truby H, Collins J. The Effect of Using Mobile Technology-Based Methods That Record Food or Nutrient Intake on Diabetes Control and Nutrition Outcomes: A Systematic Review. Nutrients. 2016;8(12). PMID: 27999302. doi: 10.3390/nu8120815.

41. Riazi H, Larijani B, Langarizadeh M, Shahmoradi L. Managing diabetes mellitus using information technology: A systematic review. Journal of diabetes and metabolic disorders. 2015;14 (1) (no pagination)(49). PMID: 605592921. doi: 10.1186/s40200-015-0174-x.

42. Rush KL, Hatt L, Janke R, Burton L, Ferrier M, Tetrault M. The efficacy of telehealth delivered educational approaches for patients with chronic diseases: A systematic review. Patient Education and Counseling. 2018;101(8):1310-21. PMID: 29486994. doi: 10.1016/j.pec.2018.02.006.

43. Russell-Minda E, Jutai J, Speechley M, Bradley K, Chudyk A, Petrella R. Health technologies for monitoring and managing diabetes: a systematic review. Journal of Diabetes Science and Technology. 2009;3(6):1460-71. PMID: 20144402. doi: 10.1177/193229680900300628.

44. Saffari M, Ghanizadeh G, Koenig HG. Health education via mobile text messaging for glycemic control in adults with type 2 diabetes: a systematic review and meta-analysis. Primary Care Diabetes. 2014;8(4):275-85. PMID: 24793589. doi: 10.1016/j.pcd.2014.03.004.

45. Shen Y, Wang F, Zhang X, Zhu X, Sun Q, Fisher E, et al. Effectiveness of Internet-Based Interventions on Glycemic Control in Patients With Type 2 Diabetes: Meta-Analysis of Randomized Controlled Trials. Journal of medical Internet research. 2018;20(5):e172. PMID: 29735475. doi: 10.2196/jmir.9133.

46. Spencer-Bonilla G, Ponce OJ, Rodriguez-Gutierrez R, Alvarez-Villalobos N, Erwin PJ, Larrea-Mantilla L, et al. A systematic review and meta-analysis of trials of social network interventions in type 2 diabetes. BMJ open. 2017;7(8):e016506. PMID: 28827256. doi: 10.1136/bmjopen-2017-016506.

47. Su D, McBride C, Zhou J, Kelley MS. Does nutritional counseling in telemedicine improve treatment outcomes for diabetes? A systematic review and meta-analysis of results from 92 studies. Journal of Telemedicine and Telecare. 2016;22(6):333-47. PMID: 26442959. doi: 10.1177/1357633X15608297.

48. Su D, Zhou J, Kelley MS, Michaud TL, Siahpush M, Kim J, et al. Does telemedicine improve treatment outcomes for diabetes? A meta-analysis of results from 55 randomized controlled trials. Diabetes Research and Clinical Practice. 2016;116:136-48. PMID: 27321329. doi: 10.1016/j.diabres.2016.04.019.

49. Suksomboon N, Poolsup N, Nge YL. Impact of phone call intervention on glycemic control in diabetes patients: a systematic review and meta-analysis of randomized, controlled trials. PloS One. 2014;9(2):e89207. PMID: 24586596. doi: 10.1371/journal.pone.0089207.

50. Sun C, Malcolm JC, Wong B, Shorr R, Doyle M-A. Improving Glycemic Control in Adults and Children With Type 1 Diabetes With the Use of Smartphone-Based Mobile Applications: A Systematic Review. Canadian Journal of Diabetes. 2018;43:51-8. PMID: 30026048. doi: 10.1016/j.jcjd.2018.03.010.

51. Tchero H, Kangambega P, Briatte C, Brunet-Houdard S, Retali G-R, Rusch E. Clinical Effectiveness of Telemedicine in Diabetes Mellitus: A Meta-Analysis of 42 Randomized Controlled Trials. Telemedicine Journal and E-Health: The Official Journal of the American Telemedicine Association. 2018;7:569-83. PMID: 30124394. doi: 10.1089/tmj.2018.0128.

52. Tildesley HD, Po MD, Ross SA. Internet Blood Glucose Monitoring Systems Provide Lasting Glycemic Benefit in Type 1 and 2 Diabetes: A Systematic Review. Medical Clinics of North America. 2015 01 Jan;99(1):17-33. PMID: 600667398. doi: 10.1016/j.mcna.2014.08.019.

53. Toma T, Athanasiou T, Harling L, Darzi A, Ashrafian H. Online social networking services in the management of patients with diabetes mellitus: systematic review and meta-analysis of randomised controlled trials. Diabetes Research and Clinical Practice. 2014;106(2):200-11. PMID: 25043399. doi: 10.1016/j.diabres.2014.06.008.

54. Vargas G, Cajita MI, Whitehouse E, Han H-R. Use of Short Messaging Service for Hypertension Management: A Systematic Review. The Journal of Cardiovascular Nursing. 2017;32(3):260-70. PMID: 27111819. doi: 10.1097/JCN.0000000000000336.

55. Verhoeven F, Tanja-Dijkstra K, Nijland N, Eysenbach G, van Gemert-Pijnen L. Asynchronous and synchronous teleconsultation for diabetes care: a systematic literature review. Journal of Diabetes Science and Technology. 2010;4(3):666-84. PMID: 20513335. doi: 10.1177/193229681000400323.

56. Wang Y, Xue H, Huang Y, Huang L, Zhang D. A Systematic Review of Application and Effectiveness of mHealth Interventions for Obesity and Diabetes Treatment and Self-Management. Advances in nutrition (Bethesda, Md). 2017;8(3):449-62. PMID: 28507010. doi: 10.3945/an.116.014100.

57. Wu Y, Yao X, Vespasiani G, Nicolucci A, Dong Y, Kwong J, et al. Mobile App-Based Interventions to Support Diabetes Self-Management: A Systematic Review of Randomized Controlled Trials to Identify Functions Associated with Glycemic Efficacy. JMIR mHealth and uHealth. 2017;5(3):e35. PMID: 28292740. doi: 10.2196/mhealth.6522.

58. Wu IXY, Kee JCY, Threapleton DE, Ma R, Lam VCK, Lee EKP, et al. Effectiveness of smartphone technologies on glycaemic control in patients with type 2 diabetes: systematic review with meta-analysis of 17 trials. Obes Rev. 2018. PMID: 29345109. doi: 10.1111/obr.12669.

59. Wu C, Wu Z, Yang L, Zhu W, Zhang M, Zhu Q, et al. Evaluation of the clinical outcomes of telehealth for managing diabetes: A PRISMA-compliant meta-analysis. Medicine. 2018;97(43):e12962. PMID: 30412116. doi: 10.1097/md.0000000000012962.

60. Yoshida Y, Boren SA, Soares J, Popescu M, Nielson SD, Simoes EJ. Effect of Health Information Technologies on Glycemic Control Among Patients with Type 2 Diabetes. Current diabetes reports. 2018;18(12):130-. PMID: 30338403. doi: 10.1007/s11892-018-1105-2.

61. Zhai Y-k, Zhu W-j, Cai Y-l, Sun D-x, Zhao J. Clinical- and cost-effectiveness of telemedicine in type 2 diabetes mellitus: a systematic review and meta-analysis. Medicine. 2014;93(28):e312. PMID: 25526482. doi: 10.1097/MD.0000000000000312.

62. Flodgren G, Rachas A, Farmer AJ, Inzitari M, Shepperd S. Interactive telemedicine: effects on professional practice and health care outcomes. Cochrane Database Syst Rev. 2015 (9):Cd002098. PMID: 26343551. doi: 10.1002/14651858.CD002098.pub2.

63. Heitkemper EM, Mamykina L, Travers J, Smaldone A. Do health information technology self-management interventions improve glycemic control in medically underserved adults with diabetes? A systematic review and meta-analysis. Journal of the American Medical Informatics Association : JAMIA. 2017;24(5):1024-35. PMID: 28379397. doi: 10.1093/jamia/ocx025.

64. Kim Y, Park J-E, Lee B-W, Jung C-H, Park D-A. Comparative effectiveness of telemonitoring versus usual care for type 2 diabetes: A systematic review and meta-analysis. Journal of Telemedicine and Telecare. 2018:1357633X18782599. PMID: 30012042. doi: 10.1177/1357633X18782599.

65. Lepard MG, Joseph AL, Agne AA, Cherrington AL. Diabetes self-management interventions for adults with type 2 diabetes living in rural areas: a systematic literature review. Current Diabetes Reports. 2015;15(6):608. PMID: 25948497. doi: 10.1007/s11892-015-0608-3.

66. Lieber BA, Taylor B, Appelboom G, Prasad K, Bruce S, Yang A, et al. Meta-analysis of telemonitoring to improve HbA1c levels: promise for stroke survivors. Journal of Clinical Neuroscience: Official Journal of the Neurosurgical Society of Australasia. 2015;22(5):807-11. PMID: 25791996. doi: 10.1016/j.jocn.2014.11.009.

67. Viana LV, Gomes MB, Zajdenverg L, Pavin EJ, Azevedo MJ, Brazilian Type 1 Diabetes Study G. Interventions to improve patients' compliance with therapies aimed at lowering glycated hemoglobin (HbA1c) in type 1 diabetes: systematic review and meta-analyses of randomized controlled clinical trials of psychological, telecare, and educational interventions. Trials. 2016;17:94. PMID: 26888087. doi: 10.1186/s13063-016-1207-6.

68. de Jong CC, Ros WJ, Schrijvers G. The effects on health behavior and health outcomes of Internet-based asynchronous communication between health providers and patients with a chronic condition: a systematic review. Journal of medical Internet research. 2014;16(1):e19. PMID: 24434570. doi: 10.2196/jmir.3000.

69. Dellifraine JL, Dansky KH. Home-based telehealth: a review and meta-analysis. Journal of Telemedicine and Telecare. 2008;14(2):62-6. PMID: 18348749. doi: 10.1258/jtt.2007.070709.

70. Hersh WR, Helfand M, Wallace J, Kraemer D, Patterson P, Shapiro S, et al. Clinical outcomes resulting from telemedicine interventions: a systematic review. BMC medical informatics and decision making. 2001;1:5. PMID: 11737882. doi: 10.1186/1472-6947-1-5.

71. Samoocha D, Bruinvels DJ, Elbers NA, Anema JR, van der Beek AJ. Effectiveness of web-based interventions on patient empowerment: a systematic review and meta-analysis. Journal of medical Internet research. 2010 2010/06/24/;12(2):e23. PMID: 20581001. doi: 10.2196/jmir.1286.

72. van den Berg N, Schumann M, Kraft K, Hoffmann W. Telemedicine and telecare for older patients--a systematic review. Maturitas. 2012;73(2):94-114. PMID: 22809497. doi: 10.1016/j.maturitas.2012.06.010.

73. Yasmin F, Banu B, Zakir SM, Sauerborn R, Ali L, Souares A. Positive influence of short message service and voice call interventions on adherence and health outcomes in case of chronic disease care: a systematic review. BMC Med Inform Decis Mak. 2016;16:46. PMID: 27106263. doi: 10.1186/s12911-016-0286-3.

74. Agarwal R, Bills JE, Hecht TJW, Light RP. Role of home blood pressure monitoring in overcoming therapeutic inertia and improving hypertension control: A systematic review and meta-analysis. Hypertension. 2011 January;57(1):29-38. PMID: 21115879 doi: 10.1161/HYPERTENSIONAHA.110.160911.

75. Anglada-Martinez H, Riu-Viladoms G, Martin-Conde M, Rovira-Illamola M, Sotoca-Momblona JM, Codina-Jane C. Does mHealth increase adherence to medication? Results of a systematic review. Int J Clin Pract. 2015;69(1):9-32. PMID: 25472682. doi: 10.1111/ijcp.12582.

76. Bassi N, Karagodin I, Wang S, Vassallo P, Priyanath A, Massaro E, et al. Lifestyle modification for metabolic syndrome: a systematic review. Am J Med. 2014;127(12):1242.e1-10. PMID: 25004456. doi: 10.1016/j.amjmed.2014.06.035.

77. Breaux-Shropshire TL, Judd E, Vucovich LA, Shropshire TS, Singh S. Does home blood pressure monitoring improve patient outcomes? A systematic review comparing home and ambulatory blood pressure monitoring on blood pressure control and patient outcomes. Integrated Blood Pressure Control. 2015 03 Jul;8:43-9. PMID: 611684137. doi: 10.2147/IBPC.S49205.

78. Cappuccio FP, Kerry SM, Forbes L, Donald A. Blood pressure control by home monitoring: meta-analysis of randomised trials. BMJ (Clinical research ed). 2004;329(7458):145. PMID: 15194600. doi: 10.1136/bmj.38121.684410.AE.

79. Christensen Jan, Valentiner Laura Staun, Petersen Rikke Juelsgaard, Henning L. The Effect of Game-Based Interventions in Rehabilitation of Diabetics: A Systematic Review and Meta-Analysis Telemedicine and e-Health. 2016;22(10):9. PMID: 27042966. doi: 10.1089/tmj.2015.0165.

80. Jaana M, Pare G. Home telemonitoring of patients with diabetes: a systematic assessment of observed effects. Journal of evaluation in clinical practice. 2007;13(2):242-53. PMID: 17378871. doi: 10.1111/j.1365-2753.2006.00686.x.

81. Kastner M, Cardoso R, Lai Y, Treister V, Hamid JS, Hayden L, et al. effectiveness of interventions for managing multiple high-burden chronic diseases in older adults: A systematic review and meta-analysis. Cmaj. 2018;190(34):E1004-E12. PMID: 623657353. doi: 10.1503/cmaj.171391.

82. Kuo A, Dang S. Secure Messaging in Electronic Health Records and Its Impact on Diabetes Clinical Outcomes: A Systematic Review. Telemed J E Health. 2016;22(9):769-77. PMID: 27027337. doi: 10.1089/tmj.2015.0207.

83. McLean G, Band R, Saunderson K, Hanlon P, Murray E, Little P, et al. Digital interventions to promote self-management in adults with hypertension systematic review and meta-analysis. Journal of Hypertension. 2016;34(4):600-12. PMID: 608230147. doi: 10.1097/HJH.0000000000000859.

84. Montani S, Bellazzi R, Quaglini S, d'Annunzio G. Meta-analysis of the effect of the use of computer-based systems on the metabolic control of patients with diabetes mellitus. Diabetes Technol Ther. 2001;3(3):347-56. PMID: 11762513. doi: 10.1089/15209150152607123.

85. Tao D, Or CK. Effects of self-management health information technology on glycaemic control for patients with diabetes: a meta-analysis of randomized controlled trials. J Telemed Telecare. 2013;19(3):133-43. PMID: 23563018. doi: 10.1177/1357633x13479701.

86. Tucker KL, Sheppard JP, Stevens R, Bosworth HB, Bove A, Bray EP, et al. Self-monitoring of blood pressure in hypertension: A systematic review and individual patient data meta-analysis. PLoS medicine. 2017;14(9):e1002389. PMID: 28926573. doi: 10.1371/journal.pmed.1002389.

87. Xiong S, Berkhouse H, Schooler M, Pu W, Sun A, Gong E, et al. Effectiveness of mHealth Interventions in Improving Medication Adherence Among People with Hypertension: a Systematic Review. Current hypertension reports. 2018;20(10):86. doi: 10.1007/s11906-018-0886-7.

88. Adu MD, Malabu UH, Callander EJ, Malau-Aduli AE, Malau-Aduli BS. Considerations for the Development of Mobile Phone Apps to Support Diabetes Self-Management: Systematic Review. JMIR mHealth and uHealth. 2018;6(6):e10115. PMID: 29929949. doi: 10.2196/10115.

89. Alvarado MM, Kum HC, Gonzalez Coronado K, Foster MJ, Ortega P, Lawley MA. Barriers to Remote Health Interventions for Type 2 Diabetes: A Systematic Review and Proposed Classification Scheme. Journal of medical Internet research. 2017;19(2):e28. PMID: 28193598. doi: 10.2196/jmir.6382.

90. Barlow J, Singh D, Bayer S, Curry R. A systematic review of the benefits of home telecare for frail elderly people and those with long-term conditions. Journal of Telemedicine and Telecare. 2007 2007;13(4):172-9. PMID: 17565772 doi: 10.1258/135763307780908058.

91. Ramadas A, Quek KF, Chan CKY, Oldenburg B. Web-based interventions for the management of type 2 diabetes mellitus: a systematic review of recent evidence. International Journal of Medical Informatics. 2011;80(6):389-405. doi: 10.1016/j.ijmedinf.2011.02.002.

92. van Vugt M, de Wit M, Cleijne WH, Snoek FJ. Use of behavioral change techniques in web-based self-management programs for type 2 diabetes patients: systematic review. Journal of medical Internet research. 2013;15(12):e279. PMID: 24334230. doi: 10.2196/jmir.2800.

93. Hadjiconstantinou M, Byrne J, Bodicoat DH, Robertson N, Eborall H, Khunti K, et al. Do Web-Based Interventions Improve Well-Being in Type 2 Diabetes? A Systematic Review and Meta-Analysis. Journal of medical Internet research. 2016;18(10):e270. PMID: 27769955. doi: 10.2196/jmir.5991.

94. Jalil S, Myers T, Atkinson I. A meta-synthesis of behavioral outcomes from telemedicine clinical trials for type 2 diabetes and the Clinical User-Experience Evaluation (CUE). J Med Syst. 2015;39(3):28. PMID: 25677954. doi: 10.1007/s10916-015-0191-9.

95. Joiner KL, Nam S, Whittemore R. Lifestyle interventions based on the diabetes prevention program delivered via eHealth: A systematic review and meta-analysis. Prev Med. 2017;100:194-207. PMID: 28456513. doi: 10.1016/j.ypmed.2017.04.033.

96. Pereira K, Phillips B, Johnson C, Vorderstrasse A. Internet Delivered Diabetes Self-Management Education: A Review. Diabetes Technol Ther. 2015;17(1):55-63. PMID: 25238257. doi: 10.1089/dia.2014.0155.

97. Hanlon P. Telehealth Interventions to Support Self-Management of Long-Term Conditions: A Systematic Metareview of Diabetes, Heart Failure, Asthma, Chronic Obstructive Pulmonary Disease, and Cancer. Environmental health : a global access science source. 2017;19(5):e172. PMID: 28526671. doi: 10.1186/s12940-017-0254-0 10.2196/jmir.6688.

98. McMillan KA, Kirk A, Hewitt A, Macrury S. A Systematic and Integrated Review of Mobile-Based Technology to Promote Active Lifestyles in People with Type 2 Diabetes. Journal of Diabetes Science and Technology. 2017;11(2):299-307. PMID: 614716290. doi: 10.1177/1932296816656018.

99. Peterson A. Improving type 1 diabetes management with mobile tools: a systematic review. Journal of Diabetes Science and Technology. 2014;8(4):859-64. PMID: 24876414. doi: 10.1177/1932296814529885.

100. Bossen D, Veenhof C, Dekker J, De Bakker D. The effectiveness of self-guided web-based physical activity interventions among patients with a chronic disease: A systematic review. Journal of Science and Medicine in Sport. 2012 December;15 (SUPPL.1):S202. PMID: 70968591. doi: 10.1016/j.jsams.2012.11.493.

101. Chowdhury FM, Ayala C, Dalmat D, Shantharam S, Chang T, Russell Z, et al. Effectiveness of telehealth on hypertension management among disparate populations: A systematic review. Circulation: Cardiovascular Quality and Outcomes Conference: American Heart Association's Quality of Care and Outcomes Research. 2017;10(Supplement 3). PMID: 621102153.

102. Kebede M, Christianson L, Khan Z, Heise TL, Pischke CR. Effectiveness of behavioral change techniques employed in eHealth interventions designed to improve glycemic control in persons with poorly controlled type 2 diabetes: a systematic review and meta-analysis protocol. Systematic reviews. 2017;6(1):211. PMID: 29065911. doi: 10.1186/s13643-017-0609-1.

103. Nangrani N, Malabu U, Vangaveti V. Outcomes of telehealth in the management of type 2 diabetes-a systematic review and meta-analysis of randomised controlled trials. Diabetes. 2018;67 (Supplement 1):A348.

104. Park DA, Kim J, Park JE. Clinical and comparative effectiveness of telemonitoring intervention versus usual care for hypertension: A systematic review and meta-analysis. Value in Health. 2016 November;19 (7):A637. PMID: 613236549.

105. Park DA, Kim YJ, Park JE. Clinical and comparative effectiveness of telemonitoring intervention versus usual care for type 2 diabetes mellitus: A systematic review and meta-analysis. Value in Health. 2016;19 (7):A605. PMID: 613237004.

106. Tucker K, Sheppard JP, Stevens R, Bosworth HB, Bove A, Bray EP, et al. Individual patient data meta-analysis of self-monitoring of blood pressure (BP-smart). Journal of Hypertension. 2016 September;34 (Supplement 2):e69-e70. PMID: 617794384. doi: 10.1097/01.hjh.0000491517.19347.e5.

107. Medical Advisory Secretariat. Home telemonitoring for type 2 diabetes: an evidence-based analysis. Ontario health technology assessment series. 2009;9(24):1-38. PMID: 23074529.

108. Chandak A, Joshi A. Self-management of hypertension using technology enabled interventions in primary care settings. Technology and Health Care: Official Journal of the European Society for Engineering and Medicine. 2015;23(2):119-28. PMID: 25515051. doi: 10.3233/THC-140886.

109. Duan Y, Xie Z, Dong F, Wu Z, Lin Z, Sun N, et al. Effectiveness of home blood pressure telemonitoring: a systematic review and meta-analysis of randomised controlled studies. Journal of Human Hypertension. 2017;31(7):427-37. PMID: 28332506. doi: 10.1038/jhh.2016.99.

110. Hu Y, Wen X, Wang F, Yang D, Liu S, Li P, et al. Effect of telemedicine intervention on hypoglycaemia in diabetes patients: A systematic review and meta-analysis of randomised controlled trials. Journal of Telemedicine and Telecare. 2018 2018/01/01/:1357633X18776823. PMID: 29909748. doi: 10.1177/1357633X18776823.

111. Kongstad MB, Valentiner LS, Ried-Larsen M, Walker KC, Juhl CB, Langberg H. Effectiveness of remote feedback on physical activity in persons with type 2 diabetes: A systematic review and meta-analysis of randomized controlled trials. J Telemed Telecare. 2017;25(1):26–34. PMID: 28958212. doi: 10.1177/1357633x17733772.

112. Mignerat M, Lapointe L, Vedel I. Using telecare for diabetic patients: A mixed systematic review. Health Policy and Technology. 2014;3(2):90-112. PMID: 53003104. doi: 10.1016/j.hlpt.2014.01.004.

113. So CF, Chung JW. Telehealth for diabetes self-management in primary healthcare: A systematic review and meta-analysis. Journal of Telemedicine and Telecare. 2018;24(5):356-64. PMID: 28463033. doi: 10.1177/1357633X17700552.

114. Uhlig K, Patel K, Ip S, Kitsios GD, Balk EM. Self-measured blood pressure monitoring in the management of hypertension: A systematic review and meta-analysis. Annals of Internal Medicine. 2013;159(3):185-94. PMID: 369507439. doi: 10.7326/0003-4819-159-3-201308060-00008.

115. Verberk WJ, Kessels AGH, Thien T. Telecare is a valuable tool for hypertension management, a systematic review and meta-analysis. Blood Pressure Monitoring. 2011;16(3):149-55. PMID: 21527847. doi: 10.1097/MBP.0b013e328346e092.

116. Hou C, Carter B, Hewitt J, Francisa T, Mayor S. Do Mobile Phone Applications Improve Glycemic Control (HbA1c) in the Self-management of Diabetes? A Systematic Review, Meta-analysis, and GRADE of 14 Randomized Trials. Diabetes Care. 2016;39(11):2089-95. PMID: 27926892. doi: 10.2337/dc16-0346 10.2337/dc16-0346.

117. Krishna S, Boren SA. Diabetes self-management care via cell phone: a systematic review. J Diabetes Sci Technol. 2008;2(3):509-17. PMID: 19885219. doi: 10.1177/193229680800200324.

118. Pal K, Eastwood SV, Michie S, Farmer AJ, Barnard ML, Peacock R, et al. Computer-based diabetes self-management interventions for adults with type 2 diabetes mellitus. The Cochrane Database of Systematic Reviews. 2013 (3):CD008776. PMID: 23543567. doi: 10.1002/14651858.CD008776.pub2.

119. Verhoeven F, van Gemert-Pijnen L, Dijkstra K, Nijland N, Seydel E, Steehouder M. The contribution of teleconsultation and videoconferencing to diabetes care: a systematic literature review. Journal of medical Internet research. 2007;9(5):e37. PMID: 18093904. doi: 10.2196/jmir.9.5.e37.
