# Supplementary material for: Mapping the Evidence on the Effectiveness of Telemedicine Interventions in Diabetes, Dyslipidemia, and Hypertension: An Umbrella Review of Systematic Reviews and Meta-Analyses
Source: J Med Internet Res. 2020 Mar 18;22(3):e16791. doi: 10.2196/16791 (PMC7113804; doi:10.2196/16791)
Supplement: Multimedia Appendix 10 [file jmir_v22i3e16791_app10.doc]

# Multimedia Appendix 1 - Electronic database search strategy

The search was conducted on October 8^th^ 2018.

## Pubmed

Simply enter all these in the search bar:

(((((((("Multimorbidity"[Mesh] OR "Comorbidity"[Mesh] OR multimorbid* OR comorbid* OR co-morbid* OR multi-morbid*))) OR ((“lipid metabolism”[Mesh] OR “lipid metabolism” OR “lipid metabolisms” OR Lipid OR lipids OR metabolism OR "Lipids"[Mesh] OR hyperlipidemia OR "Lipid Metabolism Disorders"[Mesh] OR “lipid metabolism disorder” OR “lipid metabolism disorders” OR “lipid deposition” OR “lipid depositions” OR cholesterolemia OR "Cholesterol"[Mesh] OR "Metabolic Syndrome"[Mesh] OR “metabolic syndrome” OR “metabolic syndromes” OR "Hypercholesterolemia"[Mesh] OR "Dyslipidemias"[Mesh] OR Triglycerides OR “Triglycerides"[Mesh] OR LDL OR lipoprotein OR HDL))) OR ((“Hypertension”[Mesh] OR Hypertension OR HT OR HTN OR “blood pressure” OR “high blood pressure” OR HBP OR SBP OR “systolic blood pressure” OR DSP OR “diastolic blood pressure” OR "Arterial Pressure"[Mesh] OR “arterial pressure”))) OR (("Diabetes mellitus type 2" OR "Diabetes mellitus type II" OR "Diabetes mellitus type two" OR "type II Diabetes" OR "type two Diabetes" OR "Type 2 Diabetes" OR "Diabetes type 2" OR "Diabetes type II" OR "Diabetes type two" OR "Diabetes Mellitus, Type 2"[Mesh] OR T2D OR T2DM OR "Blood Glucose"[Mesh] OR "Blood Glucose Self-Monitoring"[Mesh] OR "Hyperglycemia"[Mesh] OR Hyperglycem* OR "Hypoglycemia"[Mesh] OR Hypoglycem* OR "Glycated Hemoglobin A"[Mesh] OR “Glucose Intolerance"[Mesh] OR IGT OR “Impaired Glucose Tolerance” OR IFG OR “Impaired Fasting Glucose” OR “blood glucose”)))) AND ((Telemedicine OR telemed* OR telehealth OR Telematic* OR ehealth OR e-health OR E-Health OR “electronic health” OR mhealth OR m-health OR “mobile health” OR emedicine* OR e-medicine* OR “e medicine“ OR “electronic medicine” OR “e therapy“ OR “e therapies” OR etherap* OR e-therap* OR teletherap* OR “digital health” OR “remote consultation” OR econsult* OR e-consult* OR teleconsult* OR videoconferenc* OR video-conferenc* OR teleconferenc* OR telehome OR telemanag* OR telenurs* OR telereha* OR tele-reha* OR telemonitor* OR tele-monitor* OR “home monitoring“ OR home-monitor* OR e-mail OR “electronic mail” OR “short message” OR “short messaging” OR “electronic messaging” OR SMS OR app OR “mobile application” OR “mobile applications” OR phone OR “social network“ OR e-learning OR „electronic learning” OR web-based OR “web based” OR telemedicine[Mesh]))) AND ((“systematic review“ OR meta-analysis OR metaanalysis OR “meta analysis“ OR "Review Literature as Topic"[Mesh] OR "Network Meta-Analysis"[Mesh] OR "Meta-Analysis as Topic"[Mesh] OR "Meta-Analysis" [Publication Type] OR “Systematic Literature Review”))

🡪 724

## Embase

Simply enter the single terms in the “Freitextsuche” bar and adjust for the scope of the subject headings if necessary.

**ID Search Hits**

#1 "Diabetes mellitus type 2" or "Diabetes mellitus type II" or 571.871

"Diabetes mellitus type two" or "type II Diabetes" or "type two Diabetes"

or "Type 2 Diabetes" or "Diabetes type 2" or "Diabetes type II" or

"Diabetes type two").af. or "non insulin dependent diabetes mellitus".sh.

or (T2D or T2DM).af. or "glucose blood level".sh. or

"blood glucose monitoring".sh. or hyperglycemia.sh. or Hyperglycem*.af. or hypoglycaemia.sh. or Hypoglycem*.af. or "glycosylated haemoglobin".sh. or

"glucose intolerance".sh. or (IGT or "Impaired Glucose Tolerance" or IFG or

"Impaired Fasting Glucose" or "blood glucose").af.

#2 hypertension.sh. or (Hypertension or HT or HTN or "blood pressure" or 1.266.424

"high blood pressure" or HBP or SBP or "systolic blood pressure" or DSP

or "diastolic blood pressure").af. or "arterial pressure".sh. or

"arterial pressure".af.

#3 lipid metabolism.sh. or ("lipid metabolism" or "lipid metabolisms" or Lipid or 2.737.740

lipids or metabolism).af. or lipid.sh. or hyperlipidemia.af. or "disorders of lipid

and lipoprotein metabolism".sh. or ("lipid metabolism disorder" or

"lipid metabolism disordersOR lipid deposition" or "lipid depositions"

or cholesterolemia).af. or cholesterol.sh. or "metabolic syndrome X".sh.

or ("metabolic syndrome" or "metabolic syndromes").af. or

hypercholesterolemia.sh. or dyslipidemia.sh. or Triglycerides.af. or

triacylglycerol.sh. or (LDL or lipoprotein or HDL).af.

#4 ("multiple chronic conditions" or comorbidity).sh. or (multimorbid* or 359.250

comorbid* or co-morbid* or multi-morbid*).af.

#5 (Telemedicine or telemed* or telehealth or Telematic* or ehealth or e-health 2.172.522

or E-Health or "electronic health" or mhealth or m-health or "mobile health"

or emedicine* or e-medicine* or "e medicine" or "electronic medicine" or

"e therapy" or "e therapies" or etherap* or e-therap* or teletherap* or

"digital health" or "remote consultation" or econsult* or e-consult* or

teleconsult* or videoconferenc* or video-conferenc* or teleconferenc*

or telehome or telemanag* or telenurs* or telereha* or tele-reha* or

telemonitor* or tele-monitor* or "home monitoring" or home-monitor* or

"electronic mail" or "short message" or "short messaging" or

"electronic messaging" or SMS or app or "mobile application" or

"mobile applications" or phone or "social network" or e-learning or

"electronic learning" or web-based or "web based").af. or telemedicine.sh.

#6 "systematic review" or meta-analysis or metaanalysis or "meta analysis".af. 370.755

or "network meta-analysis".sh. or "meta analysis".sh. or "Meta-Analysis".pt.

or "Systematic Literature Review".af.

#7 (#1 OR #2 OR ‘3 OR #4) AND #5 AND #6 779

## Cochrane Library

Simply enter all the single terms in the advanced search bar and adjust for the scope of the MESH terms if necessary:

**ID Search Hits**

#1 MeSH descriptor: [Diabetes Mellitus] explode all trees 25903

#2 "Diabetes mellitus type II" 64

#3 "Diabetes mellitus type 2" 14851

#4 "Diabetes mellitus type two" 3

#5 "type II Diabetes" 1036

#6 "type two Diabetes" 29

#7 "Type 2 Diabetes" 21723

#8 "Diabetes type 2" 169

#9 "Diabetes type two" 1

#10 "Diabetes type II" 46

#11 T2D 1820

#12 T2DM 4260

#13 MeSH descriptor: [Diabetes Mellitus, Type 2] explode all trees 14608

#14 MeSH descriptor: [Hemoglobin A] explode all trees 5200

#15 MeSH descriptor: [Diabetes Mellitus, Type 2] explode all trees 14608

#16 MeSH descriptor: [Blood Glucose] explode all trees 13939

#17 MeSH descriptor: [Blood Glucose Self-Monitoring] explode all trees 628

#18 MeSH descriptor: [Hyperglycemia] explode all trees 2477

#19 MeSH descriptor: [Hypoglycemia] explode all trees 1872

#20 Hyperglycem* 6187

#21 Hypoglycem* 13114

#22 MeSH descriptor: [Glycated Hemoglobin A] explode all trees 5104

#23 MeSH descriptor: [Glucose Intolerance] explode all trees 896

#24 "Impaired Glucose Tolerance" 2153

#25 "Impaired Fasting Glucose" 513

#26 IGT 761

#27 IFG 400

#28 "blood glucose" 22457

**#29** #1 or #2 or #3 or #4 or #5 or #6 or #7 or #11 or #12 or #13 or #14 or #15 or #16 or #17 or #18 or #19 or #22 or #23 or #24 or #25 or #26 or #27 or #28 50233

#30 MeSH descriptor: [Hypertension] explode all trees 16451

#31 Hypertension 49614

#32 HT 5565

#33 HTN 28379

#34 "blood pressure" 68540

#35 "high blood pressure" 1776

#36 HBP 188

#37 SBP 5814

#38 "systolic blood pressure" 16555

#39 DSP 132

#40 "diastolic blood pressure" 13693

#41 "Arterial Pressure" 10568

#42 MeSH descriptor: [Arterial Pressure] explode all trees 330

**#43** #30 or #31 or #32 or #33 or #34 or #35 or #36 or #37 or #38 or #39 or #40 or #41 or #42 105572

#44 "lipid metabolism" 3667

#45 "lipid metabolisms" 14

#46 MeSH descriptor: [Lipid Metabolism] explode all trees 1629

#47 lipid 27591

#48 lipids 14156

#49 metabolism 88801

#50 MeSH descriptor: [Lipids] explode all trees 44911

#51 hyperlipidemia 3616

#52 MeSH descriptor: [Lipid Metabolism Disorders] explode all trees 7002

#53 "Lipid Metabolism Disorder" 15

#54 "Lipid Metabolism Disorders" 49

#55 "lipid deposition" 56

#56 "lipid depositions" 1

#57 cholesterolemia 71

#58 MeSH descriptor: [Cholesterol] explode all trees 9703

#59 MeSH descriptor: [Metabolic Syndrome] explode all trees 1487

#60 "Metabolic Syndrome" 5113

#61 "Metabolic Syndromes" 114

#62 MeSH descriptor: [Hypercholesterolemia] explode all trees 3075

#63 MeSH descriptor: [Dyslipidemias] explode all trees 6599

#64 Triglycerides 13113

#65 MeSH descriptor: [Triglycerides] explode all trees 6035

#66 LDL 14943

#67 HDL 11935

#68 lipoprotein 19242

#69 #44 or #46 or #47 or #48 or #49 or #50 or #51 or #52 or #53 or #54 or #55 or #57 or #58 or #59 or #60 or #62 or #63 or #64 or #65 or #66 or #67 or #68 142960

#70 MeSH descriptor: [Multimorbidity] explode all trees 15

#71 MeSH descriptor: [Comorbidity] explode all trees 3388

#72 comorbid* 19118

#73 co-morbid* 19117

#74 multi-morbid* 284

**#75** #70 or #71 or #72 or #73 or #74 19306

#76 #29 or #43 or #69 #75 146287

#77 Telemedicine 2844

#78 telemed* 2918

#79 telehealth 973

#80 telematic* 67

#81 ehealth 792

#82 e-health 5217

#83 E-Health 5217

#84 “electronic health” 1105

#85 m-health 4374

#86 “mobile health” 601

#87 emedicine* 8

#88 e-medicine* 8

#89 "e medicine" 3308

#90 “electronic medicine” 5

#91 “e therapy” 4215

#92 "e therapies" 1115

#93 etherap* 35

#94 e-therap* 35

#95 teletherap* 227

#96 "digital health" 104

#97 “remote consultation” 373

#98 econsult* 24

#99 teleconsult* 514

#100 videoconferenc* 552

#101 video-conferenc* 551

#102 teleconferenc* 206

#103 telehome 18

#104 telemanag* 36

#105 telenurs* 72

#106 telereha* 378

#107 tele-reha* 378

#108 telemonitor* 768

#109 tele-monitor* 768

#110 “home monitoring” 470

#111 home-monitor* 46

#112 “electronic mail” 400

#113 “short message” 459

#114 “short messaging” 64

#115 “electronic messaging” 26

#116 SMS 1588

#117 app 1733

#118 "mobile application" 654

#119 "mobile applications" 414

#120 phone 7452

#121 "social network" 682

#122 e-learning 914

#123 "electronic learning" 29

#124 web-based 5314

#125 “web based” 5314

#126 MeSH descriptor: [Telemedicine] explode all trees 1948

**#127** #77 or #78 or #79 or #80 or #81 or #82 or #83 or #84 or #85 or #86 or #87 or #88 or #89 or #90 or #91 or #92 or #93 or #94 or #95 or #96 or #97 or #98 or #99 or #100 or #101 or #102 or #103 or #104 or #105 or #106 or #107 or #108 or #109 or #110 or #111 #112 or #113 or #114 or #115 or #116 or #117 or #118 or #119 or #120 or #121 or #122 or #123 or #124 or #125 or #126 27108

**#128** #76 and #127 5137

#129 “systematic review” 12099

#130 meta-analysis 16398

#131 metaanalysis 13879

#132 “meta analysis” 16398

#133 “Systematic Literature Review” 299

#134 MeSH descriptor: [Review Literature as Topic] explode all trees 93

#135 MeSH descriptor: [Network Meta-Analysis] explode all trees 19

#136 MeSH descriptor: [Meta-Analysis as Topic] explode all trees 282

**#137** #129 or #130 or #131 or #132 or #134 or #135 or #136 19801

**#138** #76 and #127 and #137 2207
